# An Immune-Related Gene Panel for Preoperative Lymph Node Status Evaluation in Advanced Gastric Cancer

**DOI:** 10.1155/2020/8450656

**Published:** 2020-12-07

**Authors:** Yuan Yang, Ya Zheng, Hongling Zhang, Yandong Miao, Guozhi Wu, Lingshan Zhou, Haoying Wang, Rui Ji, Qinghong Guo, Zhaofeng Chen, Jiangtao Wang, Yuping Wang, Yongning Zhou

**Affiliations:** ^1^The First Clinical Medical School, Lanzhou University, Lanzhou 730000, China; ^2^Department of Gastroenterology, The First Hospital of Lanzhou University, Lanzhou 730000, China; ^3^Key Laboratory for Gastrointestinal Diseases of Gansu Province, Lanzhou University, Lanzhou 730000, China

## Abstract

*Background and Aim*: Gastric cancer (GC) is the common leading cause of cancer-related death worldwide. Immune-related genes (IRGs) may potentially predict lymph node metastasis (LNM). We aimed to develop a preoperative model to predict LNM based on these IRGs. *Methods*: In this paper, we compared and evaluated three machine learning models to predict LNM based on publicly available gene expression data from TCGA-STAD. The Pearson correlation coefficient (PCC) method was utilized to feature selection according to its relationships with LN status. The performance of the model was assessed using the area under the curve (AUC) and F1 score. *Results*: The Naive Bayesian model showed better performance and was constructed based on 26 selected gene features, with AUCs of 0.741 in the training set and 0.688 in the test set. The F1 score in the training set and test set was 0.652 and 0.597, respectively. Furthermore, Naive Bayesian model based on 26 IRGs is the first diagnostic tool for the identification of LNM in advanced GC. *Conclusion*: These results indicate that our new methods have the value of auxiliary diagnosis with promising clinical potential.

## 1. Instruction

Gastric cancer (GC) is one of the most common gastrointestinal malignancies worldwide, accounting for 1,033,701 new cases and 782,685 deaths in 2018 [1]. Although various new diagnoses and treatments have been achieved for the management of GC, the prognosis remains unsatisfactory due to recurrence and metastasis [2]. Lymph node metastasis (LNM) is one of the most crucial indicators which influence prognosis and treatment planning in GC patients [3, 4]. Accurate preoperative identification of LN status is considered critical for treatment strategy decisions in different stages of GC patients. Unfortunately, a majority of histopathologic findings identified as efficient predictors of LNM cannot be observed preoperatively. Traditional strategies to predict the LN status was developed based on radiomics or histopathologic findings. However, these predictors based on two basic strategies were available empirically or postoperatively.

Early studies demonstrate that imaging techniques to assess the LN size is not a reliable indicator in the detection of LNM [5, 6]. The prediction accuracy of the LN status evaluation approach is often unsatisfactory due to the high false-negative rate [7]. Positron emission tomography (PET) exhibits excellent specificity for detecting LNM in GC. However, the clinical utility of PET scan is limited due to its high cost [8]. Besides, a common strategy based on histopathologic findings was usually available postoperatively, and subjectivity may exist in determination to identify the LN status. Therefore, more accurate markers for the preoperative identification of LNM are urgently needed.

Various immune-related molecules have been proven as key factors during cancer initiation and progression [9–12]. Recent immunotherapy by targeting the specific immune checkpoints has demonstrated remarkable efficacy in the clinical treatment of GC [13]. Moreover, the prognostic and adjuvant treatment value of the immune-related molecules in GC has been shown in several studies [10]. Therefore, an immune-based LN signature for GC will supplement preoperative prediction and remain to be comprehensively explored regarding postoperative treatment in GC.

Machine learning algorithms are promising approaches for disease risk prediction and diagnosis based on high-dimensional genomics data sets. They provide variable predictive measures to target classification in accordance with their predictive power. Here, we perform a systematic comparative study of three machine learning methods using public TCGA data. Evaluating prediction performance to determine LN status is suitable for approaches based on the mRNA expression data of IRGs. More specifically, a novel 26-immune-gene panel based on a Naive Bayesian classifier is used for the identification of LNM in advanced GC. An immune-related gene model based on a machine learning method can provide an individual preoperative assessment of the risk of LNM in advanced GC patients.

## 2. Methods

### 2.1. Workflow

**Figure 1:**
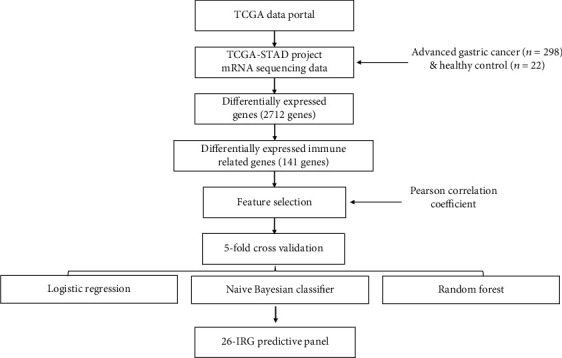
A flow chart of the study design and analysis.

The overall workflow of this study includes the following parts: (1) differentially immune-related gene analysis, (2) feature selection, (3) IRG model construction, and (4) model performance evaluation. The resulting statistically significant IRGs were subsequently subjected to the machine learning algorithm to construct an LNM prediction model (as shown in Figure 1).

### 2.2. Data Collection and Preprocessing

This study used the publicly available data from the TCGA database (https://cancergenome.nih.gov/) and the ImmPort database (https://www.immport.org/home) to do a comprehensive analysis [14]. The normalized mRNA expression profiles (HTSeq—FPKM) and corresponding clinical data of 375 tumors and 32 tumor-adjacent healthy controls were extracted from the TCGA-STAD database with the closing date of 9 December 2019. The 1811 IRGs were downloaded from the ImmPort database. The TCGA public platform was used to measure 1811 IRGs from the ImmPort database. All data were processed with R software (https://www.r-project.org/). The exclusion criteria were as follows: (1) transcriptomic data are missing or not matched; (2) the status of LNM was missing or unknown; (3) the distant metastasis has occurred, or the status of distant metastasis was unknown; and (4) diagnosed as gastric cancer but not in advanced stage (as shown in Table 1).

### 2.3. Identification of Differentially Immune-Related Genes (DEG-IRGs)

The limma package (https://www.bioconductor.org/packages/release/bioc/html/limma.html) was used to identify DEG-IRGs [15]. The Wilcoxon test was applied to estimate the gene expression changes. The DEG-IRGs were defined as genes with a false discovery rate (FDR) of less than 0.05 and with an absolute of fold change greater than 1.5 (as shown in Table S1 & S2).

### 2.4. Feature Selection and Cross-Validation

The Pearson correlation coefficient based on the filtering feature has proven to be a dimensional reduction technique [16, 17]. After data preprocessing, 298 available samples including 89 non-LNM and 209 LNM were identified and randomized into the training set and validation set based on a 5-fold random sampling of approximately equal size. This method was performed on the training set to measure the importance of feature sets based on a given measure [18]. Afterward, the machine learning algorithm is trained on the fourfold subsamples, and the rest onefold subsamples are retained as the validation set for testing the selected algorithm. The process is then repeated until the selected algorithm is validated on all the folds. Finally, the results from 5-folds would be averaged together to produce a predictive value.

### 2.5. Performance Evaluation of Classification Model

In terms of model evaluation, we used a comprehensive list of metrics that include AUC, accuracy, precision, recall, and F1 score to measure the discriminative capability. The F1 score is defined based on weighted average means of precision and recall. True positive (TP), false positive (FP), true negative (TN), and false negative (FN) were widely used for the binary classification problem. The confusion matrix is shown in Table 2. Accuracy, precision, tecall, and F1 score were applied to assess the performance of the model using the following equations:

(1) $$\begin{array}{c} \text{ Accuracy}=\frac{\text{TP}+\text{TN}}{\text{TP}+\text{FP}+\text{TN}+\text{FN}}*100,\\ \text{Precision}=\frac{\text{TP}}{\text{TP}+\text{EP}}*100,\\ \text{Recall}=\frac{\text{TP}}{\text{TP}+\text{FN}}*100,\\ \text{F}1-\text{score}=\frac{2*\text{Precision}*\text{Recall}}{\text{Precision}+\text{Recall}}*100. \end{array}$$

### 2.6. Statistical Analysis, Software, and Hardware

The data mining and relative statistical analyses were performed using R version 3.6. An adjusted *P* value of less than 0.05 was considered statistically significant. The machine learning algorithms were achieved using packages scikit-learn 0.21.1 in Python 3.7 [19]. All of the computation was conducted in a computer with a 64-bit Windows 10 operation system, Intel® Core i5-8265U CPU 1.80 GHz, and 8.0 GB installed random access memory.

## 3. Results

### 3.1. Identification of an IRG Expression Signature

**Figure 2:**
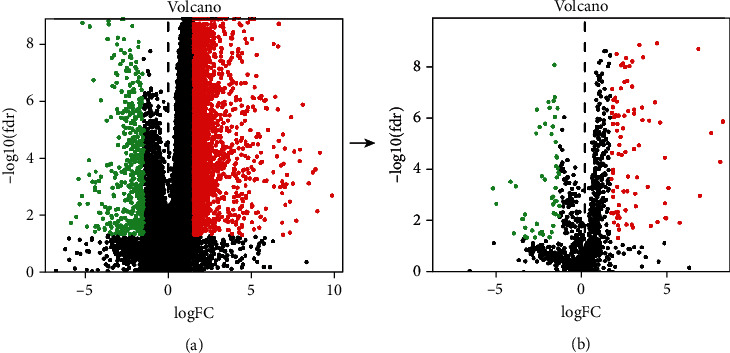
Volcano plot of all DEGs (a) and DEG-IRGs (b) in advanced gastric cancers and normal tissues. The red dots represent high-expression genes, while the green dots represent low-expression genes.

To characterize the expression pattern of immune genes, we used the limma package to analyze the TCGA FPKM data of gastric cancer and nongastric cancer samples. We identified genes as differentially expressed in GC. Afterward, we downloaded the list of IRGs from the ImmPort database. The differential expression analysis was subsequentially carried out using limma, and we obtained 141 DEGs, including 88 upregulated genes and 53 downregulated genes. A total of 141 IRGs were considered to the implication in GC (as shown in Figure 2).

### 3.2. Development of the IRG Panel for Gastric Cancer Lymph Node Metastasis

With these 141 DEGs, we further utilized feature selection, Pearson correlation coefficient, to select the best combination of immune gene signature with predictive power to classify GCs in accordance with their status of LNM. The ROC curve and F1 score were performed to determine the predictive performance of the model.

**Figure 3:**
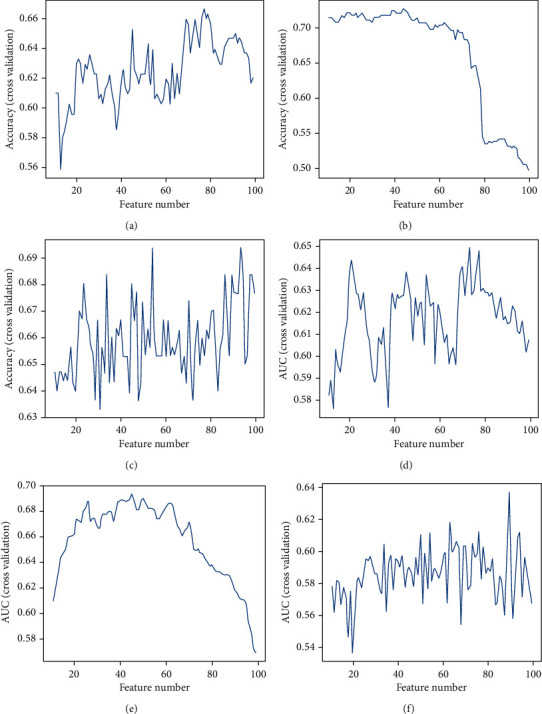
Evaluation of the LNM prediction model. The blue line represents the average area under the curve (AUC) and accuracy when the features are added. (a, d) Logistic regression model. (b, e) Naive Bayesian model. (c, f) Random forest model.

Three machine learning classifiers were performed to construct an LNM prediction model based on 298 eligible GC patients. To avoid the machine learning model from overfitting, we conducted 5-fold cross-validation in our experiment for binary classification. An optimized LNM prediction model was eventually constructed using a signature of 26 genes (as shown in Figure 3).

### 3.3. Validation and Evaluation of the Prediction Model

We first investigated the immune-related gene panels to predict LNM in advanced gastric cancer. Here, we performed 5-fold cross-validation on the training data set to evaluate the prediction model. The resulting immune gene-based diagnostic model showed good performance on the training set and test set, with AUCs of 0.741 and 0.688, respectively. Moreover, the good accuracy, precision, recall, and F1 score conformed to the generality of the Naive Bayesian classifier (as shown in Table 3).

## 4. Discussion

Although surgery has been achieved for the management of gastric cancer, it is widely accepted that advanced gastric cancer patients benefit from systemic therapies. Therefore, continuous search for new prognostic factors is helpful to select reasonable treatment strategies. Lymph node metastasis status might be the most significant prognostic indicator for the outcomes of GC patients. Accumulating evidence has suggested that the development of LNM is genetically determined with immune progression [20, 21]. To date, no immune molecular biomarkers have been confirmed to predict LNM in GC. Hence, there is an urgent need to identify an immune molecular panel with the preoperative predictive value and reveal potential malignant progression.

The prognosis and quality of life vary considerably in GC patients with or without LNM, and several studies have demonstrated associations between clinical factors and the risk of LNM [22]. Several reports have indicated that tumor size, tumor differentiation, the depth of tumor invasion, and lymphovascular infiltration were significantly associated with LNM [23–26]. However, these clinical factors still fail to achieve preoperative prediction accurately.

Machine learnings are well-established classification tools for LNM of cancers [27–30]. In recent years, combination of radiomics and machine learning has been succeeded in LNM classification due to its noninvasiveness and high efficiency. Li et al. developed a dual-energy CT-based nomogram to facilitate the preoperative prediction of LNM in GC patients and identify tumor thickness, Borrmann classification, and iodine concentration venous phase as independent predictors of LNM [31]. Feng et al. utilized lesion-based radiomic features to identify LNM with an accuracy of 76.4% preoperatively [32]. Wang et al. analyzed the values of radiomics features in the arterial phase with the random forest as feature selection and realized the individual prediction of LNM in GC [33]. However, combination of radiomics and machine learning has its exclusive challenges. Firstly, the performance of models is mainly dependent on a large number of the patient population. Extracting imaging features from a limited data set is feasible to diminish its predictive value and increase the risk of overfitting. In addition, the variability in CT or MRI image segmentation may introduce inevitable bias into the derived features.

With the rapid development of genomics in recent years, the molecular characteristics of LNM are becoming clear. To date, an increasing number of IRGs have been shown to be associated with LNM [34]. However, there are few studies on the combination of genomics and machine learning. In this study, we compared three classifiers and validated Naive Bayesian algorithm by using a genomics approach for preoperative evaluation of LN status in GC patients. First, we developed an IRG expression profile that included 141 DEGs between gastric patients and nongastric patients. Gastric mucosal tissue samples could be obtained by endoscopic biopsy preoperatively. Cancer-related gene sets were used to detect LNM in patients with GC. To refine the profiles, an immune signature of 26 genes with high predictive power for predicting LNM was extracted from the 141 DEGs using feature selection. Based on these mRNA sequencing data from the TCGA-STAD Project, our novel 26-IRG panel showed good performance. In internal validation, the selected model also showed beneficial prediction for LNM with AUC of 0.688. Our TCGA analysis showed that altered gene expression might further change in tumor progression. However, the molecular function of several genes in GC is not fully understood and deserves further investigation.

Admittedly, our study still had several limitations. First, the results were based on a public database obtained from TCGA. We did not perform further validation on a larger scale of sample size. To help address this limitation, we are comfortable with the further application of this model in our population cohort. Second, it is not clear that the performance of the model in early gastric cancer subgroup is due to the limitation of the T1 sample size. Besides, the majority of patients in this study were of the white race and the predictive performance for other racial groups is unproven. Therefore, further investigations are essential to confirm the current findings.

## 5. Conclusions

We developed a 26-mRNA-based Naive Bayesian classifier for the LN status preoperative prediction in advanced GC patients. The Naive Bayesian model based on IRGs showed outperform performance and would help clinicians guide useful individualized treatment strategies.

## Figures and Tables

**Table 1:** TCGA advanced GC patient characteristics (*n* = 298).

Clinical characteristic	Variable	No. of samples	Percentages
Gender	Male	192	64.4%
	Female	106	35.6%
Age at diagnosis	<65	124	41.6%
	≥65	172	55.7%
	Missing	2	0.7%
Grade	G1	5	1.7%
	G2	96	32.2%
	G3	191	64.1%
	Missing	6	2.0%
Stage	I	31	10.4
	II	103	34.6%
	III	141	47.3%
	IV	14	4.7%
	Missing	9	3.0%
TNM stage-T	II	71	23.8%
	III	147	49.3%
	IV	77	25.8%
	Missing	3	1.0%
TNM stage-N	N0	89	29.9%
	N1	84	28.2%
	N2	66	22.1%
	N3	59	19.8%

**Table 2:** The confusion matrix.

Confusion matrix		Predictive value	
		Negative	Positive
Actual value	Negative	TN	FP
	Positive	FN	TP

**Table 3:** The performance of the Naive Bayes classifier.

Evaluation index	Data set	Result
Accuracy	Training set	0.749
	Test set	0.720
Precision	Training set	0.708
	Test set	0.652
Recall	Training set	0.641
	Test set	0.600
F1-score	Training set	0.653
	Test set	0.597
AUC	Training set	0.741
	Test set	0.688

## Data Availability

The data that support the findings of this study are available from the TCGA or the corresponding authors upon reasonable request.

## Supplementary Materials

Supplementary 1 Table S1: the expression profile of differentially genes in gastric cancer.

Supplementary 2 Table S2: the expression profile of differentially immune-related genes in gastric cancer.

## References

[1] Bray F., Ferlay J., Soerjomataram I., Siegel R. L., Torre L. A., Jemal A. (2018). Global cancer statistics 2018: GLOBOCAN estimates of incidence and mortality worldwide for 36 cancers in 185 countries. *CA: A Cancer Journal for Clinicians*.

[2] Rugge M., Genta R. M., di Mario F. (2017). Gastric cancer as preventable disease. *Clinical Gastroenterology and Hepatology*.

[3] Pedrazzani C., de Manzoni G., Marrelli D. (2007). Nodal staging in adenocarcinoma of the gastro-esophageal junction. Proposal of a specific staging system. *Annals of Surgical Oncology*.

[4] Saito H., Fukumoto Y., Osaki T. (2007). Prognostic significance of level and number of lymph node metastases in patients with gastric cancer. *Annals of Surgical Oncology*.

[5] Cidon E. U., Cuenca I. J. (2009). Gastric adenocarcinoma: is computed tomography (CT) useful in preoperative staging?. *Clinical medicine. Oncology*.

[6] Seevaratnam R., Cardoso R., Mcgregor C. (2012). How useful is preoperative imaging for tumor, node, metastasis (TNM) staging of gastric cancer? A meta-analysis. *Gastric Cancer*.

[7] Saito T., Kurokawa Y., Takiguchi S. (2015). Accuracy of multidetector-row CT in diagnosing lymph node metastasis in patients with gastric cancer. *European Radiology*.

[8] Kudou M., Kosuga T., Kubota T. (2018). Value of preoperative PET-CT in the prediction of pathological stage of gastric cancer. *Annals of Surgical Oncology*.

[9] Yang W., Lai Z., Li Y. (2019). Immune signature profiling identified prognostic factors for gastric cancer. *Chinese Journal of Cancer Research*.

[10] Hong H., Wang Q., Li J., Liu H., Meng X., Zhang H. (2019). Aging, cancer and immunity. *Journal of Cancer*.

[11] Ito S., Fukagawa T., Noda M. (2018). Prognostic impact of immune-related gene expression in preoperative peripheral blood from gastric cancer patients. *Annals of Surgical Oncology*.

[12] Cui Y., Yu S., Zhu M. (2020). Identifying predictive factors of recurrence after radical resection in gastric cancer by RNA immune-oncology panel. *Journal of Cancer*.

[13] Liu J., Li H., Sun L., Yuan Y., Xing C. (2020). Profiles of PD-1, PD-L1, PD-L2 in gastric cancer and their relation with mutation, immune infiltration, and survival. *BioMed Research International*.

[14] Bhattacharya S., Dunn P., Thomas C. G. (2018). ImmPort, toward repurposing of open access immunological assay data for translational and clinical research. *Scientific Data*.

[15] Ritchie M. E., Phipson B., Wu D. (2015). limma powers differential expression analyses for RNA-sequencing and microarray studies. *Nucleic Acids Research*.

[16] James G., Witten D., Hastie T., Tibshirani R. (2013). *An Introduction to Statistical Learning-With Applications in R*.

[17] Witten I. H., Frank E. (1999). *Data Mining: Practical Machine Learning Tools and Techniques with Java Implementations*.

[18] Guyon I., Gunn S., Hur A. B., Dror G. Result analysis of the nips 2003 feature selection challenge.

[19] Pedregosa F., Varoquaux G., Gramfort A. (2011). scikit-learn: machine learning in Python. *Journal of Machine Learning Research*.

[20] Deng J. Y., Liang H. (2014). Clinical significance of lymph node metastasis in gastric cancer. *World Journal of Gastroenterology*.

[21] Sarela A. I., Turnbull A. D., Coit D. G., Klimstra D., Brennan M. F., Karpeh M. S. (2003). Accurate lymph node staging is of greater prognostic importance than subclassification of the T2 category for gastric adenocarcinoma. *Annals of Surgical Oncology*.

[22] Nakamoto J., Torisu R., Aoki R. (2007). Clinicopathological evaluation of biological behavior of submucosal invasive gastric carcinomas: relationship among lymph node metastasis, mucin phenotype and proliferative activity, *mucin phenotype and proliferative activity*. *The Journal of Medical Investigation*.

[23] Bu Z., Zheng Z., Li Z. (2013). Lymphatic vascular invasion is an independent correlated factor for lymph node metastasis and the prognosis of resectable T2 gastric cancer patients. *Tumor Biology*.

[24] Zhang C. D., Ning F. L., Zeng X. T., Dai D. Q. (2018). Lymphovascular invasion as a predictor for lymph node metastasis and a prognostic factor in gastric cancer patients under 70 years of age: a retrospective analysis. *International Journal of Surgery*.

[25] Liang J., Liang H., Deng J., Wang X., Wu L. (2018). Clinical study on lymph node metastasis regularity in 1456 patients with gastric cancer. *Chinese Journal of Gastrointestinal Surgery*.

[26] Park Y. D., Chung Y. J., Chung H. Y. (2008). Factors related to lymph node metastasis and the feasibility of endoscopic mucosal resection for treating poorly differentiated adenocarcinoma of the stomach. *Endoscopy*.

[27] Dihge L., Vallon-Christersson J., Hegardt C. (2019). Prediction of lymph node metastasis in breast cancer by gene expression and clinicopathological models: development and validation within a population-based cohort. *Clinical Cancer Research*.

[28] Huang C. Y., Liao K. W., Chou C. H. (2020). Pilot study to establish a novel five-gene biomarker panel for predicting lymph node metastasis in patients with early stage endometrial cancer. *Frontiers in Oncology*.

[29] Perera D., Ghossein R., Camacho N. (2019). Genomic and transcriptomic characterization of papillary microcarcinomas with lateral neck lymph node metastases. *The Journal of Clinical Endocrinology and Metabolism*.

[30] Zhang Y., Zhu Z., Sun Z., Wang Z., Zheng X., Xu H. (2014). Preoperative predicting score of lymph node metastasis for gastric cancer. *Tumor Biology*.

[31] Li J., Fang M., Wang R. (2018). Diagnostic accuracy of dual-energy CT-based nomograms to predict lymph node metastasis in gastric cancer. *European Radiology*.

[32] Feng Q. X., Liu C., Qi L. (2019). An intelligent clinical decision support system for preoperative prediction of lymph node metastasis in gastric cancer. *Journal of the American College of Radiology*.

[33] Wang Y., Liu W., Yu Y. (2020). CT radiomics nomogram for the preoperative prediction of lymph node metastasis in gastric cancer. *European Radiology*.

[34] Gentles A. J., Newman A. M., Liu C. L. (2015). The prognostic landscape of genes and infiltrating immune cells across human cancers. *Nature Medicine*.

