# Supplementary material for: An Immune-Related Gene Panel for Preoperative Lymph Node Status Evaluation in Advanced Gastric Cancer
Source: Biomed Res Int. 2020 Dec 7;2020:8450656. doi: 10.1155/2020/8450656 (PMC7789469; doi:10.1155/2020/8450656)
Supplement: Supplementary 1 — Table S1: the expression profile of differentially genes in gastric cancer. [file 8450656.f1.pdf]

gene	conMean	treatMear	logFC	pValue	fdr
TTYH3	11.82071	43.4214	1.877089	2.03E-10	9.51E-09
LINC0123!	0.099489	0.848435	3.09219	1.49E-10	7.84E-09
AKR1C4	0.380311	1.284628	1.756097	0.022016	0.036531
AC025031	0.10032	0.336601	1.746432	9.48E-05	0.000332
MYOC	45.8385	1.727174	-4.73007	1.74E-11	2.48E-09
AC105052	0.233122	0.673435	1.530453	5.87E-07	4.67E-06
SLC39A10	1.255696	5.185128	2.045893	1.27E-13	1.20E-09
MIR196A1	0.378397	1.949539	2.365162	1.47E-05	6.90E-05
AL356740.	0.06107	0.323851	2.406789	0.000132	0.000441
NDRG4	1.610113	0.546514	-1.55883	0.000211	0.000658
DNASE2B	0.062035	0.235103	1.922144	0.002779	0.006094
SPC25	1.935405	5.48544	1.502972	9.05E-09	1.64E-07
CENPF	1.564397	8.622276	2.462462	2.41E-11	2.88E-09
ATP4A	136.2253	6.402048	-4.41132	0.00048	0.001335
TMEM105	0.086409	0.253763	1.554236	0.000767	0.002
GPM6A	1.205505	0.188718	-2.67533	4.99E-07	4.10E-06
IGKV2D-3	29.15886	2.540701	-3.52064	0.024282	0.039806
LINC0159	0.103488	0.481713	2.218705	0.000854	0.0022
AC107081	0.172553	0.493861	1.517062	5.75E-08	7.12E-07
DGKG	1.832149	0.54557	-1.7477	2.32E-05	0.000101
ASCL2	4.774954	24.6662	2.368977	0.000388	0.00111
RBFOX3	0.813711	0.275177	-1.56415	0.002386	0.005336
FAR2P4	0.01966	0.345047	4.133476	0.000161	0.000521
AC020612	0.071037	0.322548	2.182867	1.66E-08	2.62E-07
C4BPAP2	0.040447	0.215182	2.411444	0.001355	0.003279
AKR1B10	248.1823	64.78946	-1.93757	0.000256	0.00078
EXO1	0.711436	3.304245	2.215514	3.38E-11	3.29E-09
PRDM8	3.628674	1.252628	-1.53448	0.000967	0.002449
C5orf34	0.456085	1.566938	1.780572	2.88E-11	3.06E-09
AL353622.	0.299483	0.867228	1.533935	8.18E-07	6.19E-06
HLA-Z	0.05363	0.26337	2.295983	0.000752	0.001968
HPDL	2.356815	9.879886	2.067656	1.35E-07	1.42E-06
RNVU1-6	0.156799	0.479488	1.612579	0.000741	0.001943
AC016735	1.591436	4.612754	1.535299	2.93E-05	0.000123
LINC0210(	1.274662	0.293552	-2.11843	2.72E-05	0.000116
RNU6-834	0.195331	0.665802	1.769172	0.000131	0.00044
AC124944	0.146562	0.568871	1.956591	5.23E-10	1.84E-08
RN7SL683	0.100404	0.308579	1.61982	0.001429	0.003437
GADD45B	52.20142	15.20451	-1.77959	6.34E-05	0.000236
AL078587.	0.095061	0.527978	2.473545	6.55E-08	7.91E-07
AC008429	0.099832	0.301778	1.595911	0.00064	0.001709
FGFR4	4.578763	17.78795	1.957871	6.52E-08	7.88E-07
OLR1	0.531661	3.217443	2.597335	1.97E-10	9.38E-09
AP003170	0.079699	0.24165	1.600283	6.64E-05	0.000245
LINC0010(	0.5398	1.811861	1.746974	8.67E-08	9.96E-07
CSTA	482.9438	16.269	-4.89166	0.014097	0.024802
HOXA4	5.769164	1.98734	-1.53752	8.23E-05	0.000294
ONECUT2	1.491513	5.4766	1.876504	1.55E-07	1.59E-06
LIPF	3584	258.2714	-3.79461	0.002214	0.005008
AP006748	0.03933	0.226188	2.52382	0.0017	0.003982
AC016737	0.100911	0.546172	2.436269	5.13E-08	6.50E-07
AL512506.	0.09608	0.294521	1.616061	6.92E-05	0.000254
AC083906	0.027036	0.306293	3.501974	1.55E-06	1.05E-05
AC073365	0.001385	0.570742	8.686725	8.75E-05	0.000311
GKN1	5854.337	282.2414	-4.3745	8.18E-05	0.000293
AC005391	0.128553	0.752616	2.549552	5.83E-08	7.20E-07
AC026368	0.222282	0.953583	2.100966	5.27E-06	2.93E-05

BPIFA1	0.014046	1.605702	6.836913	0.009532	0.017703
RNU6-31(	0.07583	0.221784	1.548307	0.017041	0.029239
AC108860	0.136238	0.387836	1.509322	0.000203	0.000638
SCARA5	12.83607	1.550858	-3.04906	1.00E-10	6.06E-09
FXYD1	2.328905	0.468188	-2.31449	4.52E-10	1.67E-08
AC113346	0.020637	0.434342	4.395538	8.23E-07	6.22E-06
CCL7	0.154037	0.754276	2.291816	1.41E-09	3.88E-08
LINC0052	0.032837	0.364041	3.470707	0.00013	0.000436
NANOGN	0.087179	0.249427	1.516573	5.97E-06	3.25E-05
PRR5-ARH	0.024127	0.214074	3.14939	1.05E-12	1.31E-09
AL355987.	0.122245	0.356949	1.545941	2.83E-08	4.02E-07
MIR4435-	0.588858	2.540783	2.109282	9.84E-13	1.31E-09
AC016027	0.074208	0.282317	1.927677	5.53E-06	3.06E-05
RNU6-22F	0.085008	0.24367	1.519252	0.011221	0.020359
HOXC11	0.341424	2.431687	2.832323	9.06E-10	2.80E-08
IL13RA2	0.098766	0.874795	3.146855	1.65E-06	1.10E-05
PRR11	2.370722	8.52244	1.84594	1.68E-11	2.48E-09
SPATA17	0.08174	0.243712	1.576065	1.56E-05	7.24E-05
AC090192	0.03946	0.605766	3.940309	1.14E-05	5.59E-05
LINC0101	0.081039	0.290302	1.840866	1.90E-06	1.24E-05
AL355802.	0.357278	1.192009	1.738275	4.42E-08	5.80E-07
ADH1B	18.77555	3.97058	-2.24143	1.04E-09	3.06E-08
FOXP4-AS	1.112404	3.265607	1.55367	5.85E-05	0.000221
AL121989.	0.06681	0.290018	2.118002	3.97E-06	2.31E-05
ACRV1	0.065287	0.226617	1.795392	6.10E-08	7.48E-07
RNF150	5.69962	1.786476	-1.67375	0.003773	0.007945
AC103563	1.633228	0.149579	-3.44874	3.83E-05	0.000155
DPP6	1.59466	0.196511	-3.02057	6.39E-06	3.44E-05
KRT87P	0.042603	0.262988	2.625962	5.18E-07	4.23E-06
SMC1B	0.066453	0.348813	2.392053	0.000347	0.00101
P3H4	3.46585	15.50359	2.161321	2.73E-10	1.16E-08
RIPPLY3	0.109823	0.704356	2.681128	0.022428	0.037132
FRMD5	0.320228	1.457333	2.18616	4.37E-09	9.16E-08
AL158166.	0.186635	0.569476	1.609416	2.89E-07	2.62E-06
RNFT2	0.305386	1.662921	2.445014	1.74E-12	1.40E-09
PGM2L1	0.943945	3.532166	1.903779	1.30E-12	1.32E-09
ZWILCH	1.919299	5.707508	1.572282	2.11E-11	2.71E-09
AL022345.	0.097693	0.331122	1.761039	0.000287	0.000857
MIR4768	0.318208	1.057801	1.733027	0.005614	0.011205
ZNF114	0.08958	0.401722	2.164941	0.000338	0.000987
CFD	187.9966	28.80231	-2.70645	2.14E-12	1.40E-09
WDR97	0.076793	0.223345	1.540222	5.50E-05	0.00021
AC019186	0.075917	0.237383	1.644724	1.71E-05	7.82E-05
DHRS2	0.093853	2.512334	4.742475	6.60E-05	0.000244
MIR657	0.129609	0.577711	2.15618	0.000562	0.001529
PTPRO	0.231925	1.36522	2.557405	3.29E-05	0.000136
SPTBN5	0.195781	0.577788	1.561297	0.000115	0.000392
PLAC9	12.08274	3.597502	-1.74788	1.05E-07	1.17E-06
RN7SL684	0.07056	0.246092	1.802278	4.73E-05	0.000185
FAP	0.395997	2.372595	2.582906	1.56E-10	8.04E-09
RNU6-80F	0.039162	0.270467	2.78794	6.76E-05	0.000249
MIR5685	0.097984	0.33886	1.790073	0.008007	0.015229
EIF4EP1	0.166218	0.516559	1.635857	6.09E-07	4.81E-06
AC092681	0.097685	0.375238	1.941589	1.01E-05	5.03E-05
AL117382.	0.479132	2.802014	2.547968	2.87E-07	2.61E-06
RHPN1-AS	0.223621	0.791752	1.823992	1.63E-07	1.66E-06
PWAR6	1.31952	0.381119	-1.7917	9.83E-05	0.000342
NTSR1	0.316114	1.909954	2.595022	0.027187	0.043853

AL391056.	0.18893	1.39538	2.884735	5.64E-05	0.000214
AC004009	0.202654	0.802155	1.984862	0.003865	0.008115
AC119403	0.159674	0.686725	2.104603	0.000147	0.000483
LACTB2-A	0.059336	0.252478	2.089189	1.10E-09	3.20E-08
AC087273	0.017327	0.229714	3.728736	8.25E-06	4.27E-05
S100B	9.369454	2.209127	-2.08449	2.64E-08	3.81E-07
AC112721	0.067705	0.25304	1.902025	4.25E-06	2.45E-05
C1QTNF6	0.717643	2.897721	2.01358	1.08E-11	2.22E-09
PPIAP77	0.107727	0.3583	1.733791	2.01E-06	1.30E-05
AL031666.	0.091748	0.265242	1.531559	0.000525	0.001443
AC007922	0.070065	0.215297	1.619557	0.000551	0.001503
AL513185.	0.077343	0.256184	1.727843	0.00538	0.010811
AC008440	0.217912	0.874478	2.004674	2.17E-05	9.55E-05
RNA5SP49	0.138307	0.422454	1.610921	0.001358	0.003286
AC243967	0.151639	0.574265	1.921079	0.016729	0.028778
HTRA4	0.064576	0.437345	2.75971	8.54E-10	2.69E-08
AL513185.	0.12768	0.462282	1.856238	0.000603	0.001625
RNU6-665	0.084355	0.359817	2.092727	0.001287	0.003138
PTMAP1	0.35186	1.547322	2.1367	9.71E-09	1.73E-07
AC105235	0.113194	0.405852	1.84216	5.31E-07	4.32E-06
LINC01572	0.069506	0.224841	1.693698	1.09E-08	1.88E-07
DLEU2	0.443663	1.616004	1.864894	7.71E-12	2.04E-09
CENPE	0.776234	3.439882	2.147794	9.27E-11	5.91E-09
AL390961.	0.076075	0.256809	1.755199	1.80E-05	8.14E-05
MIR5692C	0.226885	0.687029	1.59841	0.006317	0.012404
AL359075.	0.19219	1.265273	2.718842	2.28E-07	2.16E-06
SNX18P3	0.101602	0.356998	1.812989	0.00105	0.002631
SNORD99	0.688193	3.089446	2.166463	1.31E-08	2.19E-07
GPX3	97.03653	21.41519	-2.17989	1.38E-11	2.40E-09
IGLV3-6	1.365068	0.195238	-2.80566	0.024986	0.040757
AC008569	0.126271	0.404817	1.680745	5.35E-07	4.35E-06
LINC0171	0.018284	0.337458	4.206028	3.98E-10	1.51E-08
RNA5SP2(	0.273404	1.044812	1.934138	0.001185	0.002921
AC129510	0.277458	0.786728	1.503597	2.40E-07	2.25E-06
AC010976	0.097037	0.28784	1.568659	4.19E-08	5.54E-07
SLC25A4	18.40836	6.12355	-1.58792	6.88E-08	8.21E-07
SCARNA4	0.070391	0.243185	1.788583	5.28E-05	0.000203
RNA5SP15	0.058178	0.217638	1.90338	0.013667	0.024154
CPXM1	1.60229	8.253707	2.364907	1.12E-09	3.23E-08
RAB3IP	1.124715	3.661976	1.703062	2.79E-11	3.02E-09
AL139120.	0.226336	0.720863	1.671255	3.93E-06	2.29E-05
APOA2	0.079249	39.63358	8.966118	0.003335	0.007134
CA15P1	0.075039	0.213755	1.51025	0.00327	0.00701
UTAT33	0.110735	0.340103	1.618863	1.19E-06	8.45E-06
AC073415	0.344303	1.030507	1.581603	1.13E-06	8.13E-06
AC010275	0.048898	0.215078	2.137016	0.00091	0.002325
DRICH1	0.079825	0.269549	1.755631	1.10E-05	5.41E-05
RNU6-104	0.157026	0.476094	1.600243	0.001852	0.004293
AC087588	0.330561	1.354216	2.034468	1.61E-07	1.65E-06
AC093826	0.09593	0.273826	1.513203	0.007054	0.013647
KCNK9	0.135658	0.472067	1.799013	0.002018	0.00462
AC099518	0.155924	0.562109	1.850003	2.63E-07	2.43E-06
AL021407.	0.047569	0.269413	2.501727	1.37E-05	6.47E-05
ZIC5	0.091918	0.939218	3.353039	1.48E-05	6.92E-05
KIF20A	2.187633	7.650957	1.806269	1.07E-09	3.13E-08
PROM1	4.64287	13.51268	1.541226	0.001595	0.00377
NHS	0.947819	2.719515	1.520666	2.23E-07	2.13E-06
AC073575	0.114619	0.358209	1.643955	2.35E-08	3.47E-07

ZBTB46-A	0.042851	0.580393	3.759616	0.004735	0.009673
COL1A2	50.06903	192.8901	1.945789	1.03E-08	1.81E-07
RNA5SP24	0.07274	0.231571	1.670631	0.000839	0.002165
CXCL5	13.46146	71.81886	2.415528	0.006869	0.013331
AC002546	0.601438	0.177909	-1.75728	3.23E-05	0.000134
SOX15	9.951349	2.068426	-2.26636	9.76E-06	4.90E-05
MIR454	0.043359	0.24519	2.499502	0.00026	0.00079
MIOX	0.087735	0.708767	3.014087	0.00051	0.001406
LINC0177	0.600961	0.203607	-1.56148	0.021779	0.036208
IGKV2OR2	3.69345	0.24146	-3.93511	0.006571	0.012842
EPYC	0.023514	0.624274	4.730603	6.90E-06	3.66E-05
MIR135B	0.112238	0.521669	2.216569	5.14E-05	0.000198
POU5F1	0.510996	1.543467	1.594792	6.03E-05	0.000226
RNU5A-8I	0.174292	0.606138	1.79814	0.002782	0.0061
MT-TE	3.317113	1.045447	-1.66581	0.000301	0.000894
AL356740.	0.140748	0.410307	1.543584	0.003014	0.006534
RNU6-850	0.849374	3.295847	1.956177	6.14E-08	7.52E-07
AL158824.	0.121024	0.349382	1.529507	5.62E-07	4.51E-06
AC008870	0.129971	0.369037	1.505578	0.000263	0.000799
IGKV3D-7	13.07182	1.149693	-3.50714	0.009502	0.017657
PEBP4	1.527897	0.22909	-2.73756	1.41E-09	3.88E-08
APOB	47.00037	2.942697	-3.99746	0.001702	0.003984
AP006284	0.318505	0.949987	1.576593	2.77E-06	1.70E-05
PRAC2	0.186185	1.011533	2.441732	3.76E-05	0.000153
UBXN10-/	5.772033	1.004407	-2.52273	6.91E-06	3.66E-05
DUSP1	331.9223	104.5552	-1.66658	2.19E-06	1.40E-05
MIR5009	0.096042	0.387671	2.013087	0.000459	0.001285
PSD	12.67187	2.375432	-2.41537	0.000991	0.002503
TUBB3	0.110974	0.54119	2.285914	6.61E-08	7.96E-07
MYBPH	0.022669	0.326548	3.848494	0.008713	0.016381
ADGRG1	8.484127	24.52492	1.53141	1.46E-08	2.36E-07
SKA3	1.740446	7.288295	2.066125	9.72E-11	6.00E-09
BBOX1-AS	0.756306	2.581317	1.771065	1.74E-06	1.15E-05
AC006273	0.062245	0.235892	1.922105	1.84E-06	1.21E-05
RNU6-930	0.201712	0.672967	1.738236	9.75E-06	4.90E-05
MIR4295	0.055312	0.218422	1.98146	0.010561	0.019325
WNT2	0.388098	2.58307	2.734593	5.75E-11	4.42E-09
UBD	11.45295	34.06261	1.57247	2.35E-06	1.48E-05
GDPD5	0.609452	2.690107	2.142079	2.56E-09	6.06E-08
TBX4	0.021083	0.47336	4.488821	0.001307	0.003183
MIR320C1	0.194768	0.873749	2.165461	2.12E-05	9.38E-05
AC115102	0.156623	0.552696	1.819189	1.93E-06	1.26E-05
SEM1P1	0.359287	1.125409	1.647241	5.60E-05	0.000213
FAM72A	0.093064	0.418599	2.169277	3.07E-11	3.14E-09
AP003419	0.114775	0.391785	1.771253	1.67E-05	7.65E-05
SAPCD2P3	0.088563	0.2715	1.616173	1.48E-06	1.01E-05
XPNPEP2	24.78342	3.931954	-2.65606	0.002793	0.006119
AL158166.	0.369759	1.261273	1.770225	2.23E-07	2.13E-06
ENPP7P11	0.215064	0.963833	2.164019	0.000543	0.001484
CXCL9	10.09992	47.04507	2.2197	2.66E-07	2.45E-06
ZNF887P	0.111059	0.411876	1.890886	6.27E-10	2.11E-08
AC139720	0.151115	0.483767	1.678665	0.001507	0.003593
AL627309.	0.639247	2.019544	1.659583	0.000268	0.000811
PABPC1L	0.731813	4.996046	2.77124	1.91E-11	2.60E-09
HOXA11-/	0.145651	1.140382	2.968928	2.31E-08	3.43E-07
RNA5SP18	0.112285	0.575638	2.357991	4.66E-05	0.000183
PART1	1.919846	0.445234	-2.10836	2.80E-07	2.55E-06
AC020978	0.179223	0.522028	1.542369	4.46E-06	2.55E-05

MSR1	1.050822	3.654397	1.798115	6.18E-08	7.56E-07
ODF2-AS1	0.159093	0.495076	1.637781	4.12E-06	2.38E-05
RNU6-13(	0.109309	0.651661	2.575717	0.00049	0.001358
AC091057	0.346674	1.232518	1.829955	2.56E-10	1.11E-08
AL591895.	4.460823	12.76531	1.516847	3.37E-08	4.66E-07
AC022762	0.162921	0.567689	1.800925	9.45E-08	1.07E-06
IGLC5	0.989035	0.28028	-1.81915	0.012901	0.022971
MIR4787	0.89857	0.27668	-1.69941	0.015226	0.026515
IGKV1OR-	1.545231	0.229964	-2.74834	0.02272	0.037539
BAAT	0.027689	2.220307	6.325315	2.16E-10	9.96E-09
GGH	3.911492	16.71519	2.095368	2.87E-07	2.61E-06
AC007376	0.082189	0.239207	1.541247	0.000234	0.000721
MIR6783	0.043315	0.348789	3.00942	6.36E-05	0.000236
AL353691.	0.108145	0.370628	1.777003	8.52E-06	4.38E-05
F2R	6.261759	22.39161	1.838318	3.02E-11	3.13E-09
IGF2BP2-/	0.041953	0.246685	2.555815	2.79E-08	3.97E-07
RPL39P36	0.571359	1.781151	1.640339	2.81E-09	6.54E-08
SLC5A6	3.808633	11.0639	1.538515	3.08E-10	1.26E-08
TREH	5.104449	0.486847	-3.39021	0.006119	0.012071
MIR4653	1.445217	4.426057	1.614736	2.20E-05	9.67E-05
AL592301.	0.114398	0.338918	1.566873	6.56E-07	5.13E-06
AL512413.	0.161901	0.982397	2.601197	7.14E-08	8.48E-07
AL355488.	0.407068	1.424135	1.806746	7.53E-09	1.42E-07
DIAPH3	0.791071	2.336432	1.562428	5.74E-09	1.15E-07
LAMP3	2.045368	5.952918	1.541237	3.45E-06	2.04E-05
FOXD3	0.984747	0.28923	-1.76753	0.000116	0.000395
PTK7	2.729826	9.879884	1.855685	1.49E-07	1.54E-06
CSRP1	118.0443	40.87557	-1.53002	3.46E-05	0.000142
KIF15	0.783174	3.11934	1.993835	1.91E-10	9.13E-09
MIR6812	0.098971	0.467035	2.238454	8.63E-05	0.000307
LINC0242(	0.020831	0.355871	4.094575	0.008841	0.01659
TRAJ23	0.108654	0.351885	1.69536	0.005607	0.011193
NCAPH	2.076321	6.908087	1.734256	1.82E-09	4.68E-08
CYP2W1	0.916748	15.88561	4.115051	0.000787	0.002047
AP002360	0.150564	0.697143	2.21108	3.10E-07	2.77E-06
XIRP1	0.053936	0.233248	2.112541	4.67E-08	6.05E-07
SNORD12	0.378918	1.086548	1.519794	0.000882	0.002262
ETV4	1.787617	14.37577	3.007529	8.84E-11	5.71E-09
P2RX2	1.215879	0.253722	-2.26068	9.73E-10	2.94E-08
YRDCP1	0.058741	0.243884	2.053751	6.93E-07	5.38E-06
SNORD11	0.219682	0.631271	1.522841	0.001079	0.002692
KCNJ15	3.444257	1.095244	-1.65294	0.000685	0.001812
AC091057	0.152266	0.606947	1.994974	4.92E-08	6.29E-07
FBXL22	4.369903	1.513969	-1.52927	0.001608	0.003796
NAALADL	5.808566	1.24095	-2.22674	0.000193	0.000612
PDZD4	3.591926	0.981623	-1.87152	7.15E-06	3.77E-05
NNAT	14.40078	4.095415	-1.81406	0.002101	0.004781
AC136475	0.336802	1.037747	1.623481	6.44E-08	7.81E-07
AL606534.	0.08233	0.344558	2.06526	1.87E-05	8.41E-05
GLIS3-AS1	0.062655	0.241119	1.944241	0.003661	0.00774
DUTP2	0.062583	0.330506	2.400823	6.84E-09	1.31E-07
PLAC1	0.060122	0.300577	2.321756	0.007678	0.014684
RNU4-4P	0.053632	0.321794	2.58498	5.97E-05	0.000225
AL354928.	0.053695	0.226607	2.077328	1.50E-06	1.02E-05
SLED1	0.114683	0.404653	1.819039	0.001183	0.002918
NCAPD2P	0.029171	0.284373	3.285175	3.15E-08	4.41E-07
MC1R	0.214139	0.638226	1.57552	3.23E-09	7.25E-08
SNORD6	1.141315	4.178518	1.872294	3.81E-07	3.28E-06

MCRIP2P1	0.065716	0.296181	2.172172	5.09E-06	2.85E-05
AC022784	0.0165	0.271078	4.038189	8.07E-08	9.38E-07
AC025176	0.075335	0.344423	2.192799	1.59E-06	1.07E-05
ELOVL2	0.189572	0.615287	1.698518	0.018311	0.031102
M1AP	0.189102	1.046461	2.468279	0.000201	0.000632
AC137894	0.088088	0.476018	2.433994	0.002472	0.005505
ARMC2-A	0.072422	0.279351	1.947581	0.00723	0.013935
CENPI	0.443658	1.716722	1.952136	1.52E-11	2.46E-09
AC011447	0.149276	0.445215	1.576522	0.002623	0.005795
CALY	1.075228	0.264314	-2.02432	2.49E-05	0.000107
RNF207	0.915185	2.676235	1.54807	6.10E-08	7.48E-07
CSAG3	0.012999	1.413961	6.76516	4.57E-05	0.00018
CREG2	0.052674	0.521701	3.308061	7.65E-07	5.86E-06
LDB3	7.22597	1.714679	-2.07525	4.15E-06	2.39E-05
ZWINT	6.268105	17.79962	1.505745	1.01E-08	1.79E-07
CLEC3B	33.13421	3.895721	-3.08836	2.30E-10	1.03E-08
AC009268	0.058517	0.264942	2.178754	8.37E-08	9.68E-07
AC004466	0.084955	0.303524	1.837045	1.94E-06	1.26E-05
RNU6-583	0.073559	0.385938	2.391399	3.85E-05	0.000156
RNU6-762	0.185701	0.890918	2.262313	3.92E-07	3.35E-06
HMGB1P2	0.09871	0.304757	1.626391	3.56E-07	3.10E-06
AC021739	0.06407	0.242891	1.92259	1.34E-07	1.42E-06
AC027449	0.110089	0.316895	1.525335	0.024537	0.040133
AC078860	0.021355	0.229338	3.424827	3.85E-05	0.000156
RNU6-46I	0.156196	0.48097	1.622592	0.002271	0.005118
COL8A1	2.606164	8.884216	1.769316	2.16E-06	1.38E-05
FEZF1	0.059811	1.167955	4.287434	9.24E-10	2.83E-08
NCAPG	1.445708	4.978691	1.78399	3.37E-09	7.47E-08
AC097493	0.045653	0.233279	2.353263	7.26E-05	0.000265
ALKAL1	0.170198	1.525709	3.164194	1.42E-09	3.88E-08
HIST1H2B	0.01879	0.288536	3.940689	0.000904	0.002311
HOXC10	0.898443	7.470507	3.055707	3.32E-09	7.39E-08
GNGT1	0.017713	0.564786	4.994785	5.35E-05	0.000205
LEXM	1.821993	0.20272	-3.16795	0.002915	0.006348
RNU7-151	0.120472	0.350359	1.540137	0.01004	0.01852
CLDN2	3.970414	27.34376	2.78385	0.000485	0.001345
AC127526	0.086548	0.354653	2.034841	2.13E-06	1.37E-05
LINC00858	0.025224	0.406423	4.010119	1.85E-07	1.84E-06
AC091053	0.072671	0.25631	1.818442	3.17E-07	2.83E-06
RNU6-795	0.165763	0.593014	1.838944	4.23E-05	0.000169
SGCA	10.365	2.376383	-2.12488	6.32E-07	4.97E-06
MAB21L2	1.391493	0.461965	-1.59078	2.07E-05	9.17E-05
AC007347	0.079865	0.311269	1.96253	9.84E-07	7.23E-06
STRIP2	0.347496	1.682968	2.27594	9.57E-11	5.99E-09
AL662797.	0.145943	0.436587	1.580864	1.01E-09	2.99E-08
AC044839	0.015574	0.365991	4.554565	0.006369	0.012494
MYB	1.718035	5.85596	1.769146	1.50E-06	1.02E-05
SNORA24	0.044544	0.288056	2.693058	3.44E-06	2.04E-05
TMEM252	3.649067	0.904489	-2.01235	4.33E-06	2.49E-05
TMEM132	2.283421	6.531364	1.516188	5.38E-07	4.36E-06
C2CD4D	0.685799	1.988772	1.53602	3.65E-07	3.17E-06
LRRIQ4	0.090426	0.440769	2.285211	5.87E-07	4.67E-06
FNDC1	1.95779	11.63795	2.57154	9.31E-09	1.68E-07
GNAO1	6.842627	2.252137	-1.60326	0.001341	0.003251
MCEMP1	0.19736	0.621213	1.654257	0.001097	0.002731
HNF1B	1.968868	6.546282	1.73331	4.35E-07	3.66E-06
MIR3129	0.346395	1.362257	1.97551	0.005872	0.011645
CXCL1	14.62557	61.3474	2.068509	6.72E-07	5.24E-06

MT1M	15.74503	3.122673	-2.33404	9.07E-10	2.80E-08
DPF1	0.084677	0.329976	1.962315	2.40E-08	3.51E-07
AC097478	0.007963	0.282365	5.148094	2.93E-05	0.000123
KIFC1	3.15145	9.710377	1.623512	8.92E-09	1.62E-07
AP003498	0.084632	0.346125	2.032015	0.008567	0.016146
DSCR8	0.008385	0.95366	6.829544	0.000616	0.001654
SLC51B	8.979478	1.931698	-2.21676	0.00339	0.00724
CLPSL2	0.009074	0.249599	4.781787	2.22E-05	9.76E-05
RAD51	1.394684	3.969217	1.508916	1.77E-09	4.59E-08
KIF11	3.222254	10.74564	1.737609	3.71E-10	1.43E-08
EN2	0.031649	0.671233	4.406584	0.014336	0.025185
CDCA5	2.093641	7.440588	1.829402	8.80E-10	2.74E-08
AL121832.	0.04336	0.342583	2.982009	0.001381	0.003336
RNU6-122	0.223795	0.784915	1.810357	5.88E-05	0.000222
AC107072	0.092517	0.269761	1.543886	1.96E-05	8.75E-05
AL391832.	0.037634	0.212271	2.495781	1.11E-05	5.47E-05
AL121832.	0.106533	0.533025	2.322902	7.57E-10	2.45E-08
AC103996	0.074788	0.226527	1.5988	0.003802	0.007998
AL590004.	0.161674	0.564896	1.804897	0.02216	0.036743
ATP11A	2.974281	13.62521	2.195666	1.74E-12	1.40E-09
LRRN4	0.089936	0.769641	3.097222	2.86E-06	1.75E-05
HPGD	46.40773	10.41339	-2.15593	1.33E-07	1.41E-06
SNORD19	0.42826	1.397806	1.706604	1.70E-05	7.78E-05
RAB5CP1	0.090758	0.301369	1.731432	2.05E-05	9.10E-05
SIGLEC11	1.148159	0.239525	-2.26107	0.000112	0.000382
LPAR3	0.738821	0.229675	-1.68563	0.00918	0.017139
RF00569	0.276708	1.135661	2.037097	2.14E-07	2.06E-06
AC103739	0.147333	0.41867	1.506731	1.43E-06	9.77E-06
FCHO1	1.153874	3.390524	1.555023	2.17E-07	2.08E-06
HPSE2	2.785721	0.326119	-3.09458	1.96E-07	1.92E-06
MT1G	168.8175	52.72388	-1.67894	0.000468	0.001305
AL022316.	0.145553	0.726264	2.318948	7.24E-07	5.57E-06
AC007849	0.541986	1.775063	1.711541	0.002239	0.005056
FAM238B	0.012751	0.222917	4.127824	2.39E-05	0.000104
C1QTNF1-	0.077212	0.260739	1.755717	0.007708	0.014731
MIR7851	0.084528	0.309767	1.873683	0.000775	0.002018
SNX10	2.393666	7.303239	1.609315	2.79E-09	6.50E-08
NDUFA3P	0.128203	0.416441	1.699677	9.37E-05	0.000328
RNU6-57F	0.064399	0.269688	2.066175	0.000542	0.001483
AC007342	0.565065	1.867221	1.724405	5.18E-05	0.000199
PMEPA1	6.76393	38.75541	2.518464	3.79E-11	3.55E-09
RF00575	0.057033	0.244112	2.097667	0.001223	0.003004
ALDH3A1	69.08124	11.63006	-2.57044	6.54E-05	0.000242
RNU6-132	0.125946	0.473011	1.909067	0.000507	0.001399
RGS2	63.03849	20.67159	-1.60858	5.18E-07	4.23E-06
RN7SL19P	0.093968	0.308989	1.717316	1.59E-05	7.35E-05
RNU6-116	0.180042	1.355997	2.912946	4.82E-06	2.72E-05
AL359880.	0.062985	0.371749	2.561239	2.95E-08	4.17E-07
PRKN	1.5227	0.53771	-1.50173	1.44E-06	9.87E-06
AC008443	0.32458	0.919919	1.502934	0.001238	0.003036
C20orf144	0.085961	0.264044	1.619034	7.42E-09	1.40E-07
AL513217.	0.695132	0.189442	-1.87553	0.000737	0.001934
AC007128	0.026075	0.511653	4.294436	1.06E-09	3.10E-08
ALPG	0.506487	10.78031	4.41173	2.94E-07	2.66E-06
SPARC	70.24325	210.5866	1.583982	1.25E-08	2.10E-07
OSBPL3	2.587628	7.820588	1.595647	2.37E-11	2.88E-09
AL357054.	1.551564	0.494482	-1.64973	0.000413	0.001171
ABCA9	1.292077	0.451751	-1.51609	0.000685	0.001812

FOXM1	4.187632	17.2093	2.038982	2.00E-10	9.43E-09
F2	0.035496	0.804483	4.502342	0.003812	0.008017
RF02247	0.016052	0.375495	4.547961	6.84E-05	0.000252
RF01169	0.054613	0.297109	2.443661	1.72E-05	7.86E-05
CRP	0.025347	1.169784	5.528257	0.005682	0.011331
MIR5194	0.192978	0.932759	2.27307	1.47E-06	1.00E-05
LINC0181	0.066224	0.279627	2.07808	4.73E-10	1.72E-08
AC097372	0.064184	0.226599	1.819853	6.55E-06	3.51E-05
AC005522	0.055549	0.276375	2.3148	3.28E-07	2.92E-06
PDK4	98.77031	18.75195	-2.39704	1.42E-07	1.48E-06
MIR4530	1.126719	0.354209	-1.66946	0.001149	0.002846
ULBP1	0.054206	0.391897	2.853961	5.13E-08	6.50E-07
SNORA74	0.106114	0.37528	1.822348	7.61E-06	3.98E-05
RNU6-12	0.11112	0.703263	2.661947	8.67E-07	6.49E-06
AC046143	0.325129	0.933784	1.522078	7.06E-08	8.40E-07
HS3ST2	0.299053	1.328711	2.151553	4.39E-05	0.000174
ASPM	0.978912	4.276198	2.127077	2.81E-10	1.19E-08
AC092919	0.067043	0.257721	1.942656	0.005249	0.01058
C2orf40	53.39315	6.137684	-3.12089	1.40E-08	2.29E-07
AC096745	1.366017	0.407574	-1.74484	0.000405	0.001152
AC103957	0.180969	0.527271	1.542802	0.01351	0.023917
AC008514	0.17585	1.273365	2.856226	0.000199	0.000627
RNA5SP2	0.08529	0.290501	1.768086	0.00574	0.011424
AC016394	0.578685	1.80022	1.637324	3.60E-10	1.40E-08
COL27A1	1.202293	3.854501	1.680755	7.79E-07	5.93E-06
CNKSR2	1.13913	0.301725	-1.91663	0.00197	0.004522
MIR378G	0.105004	0.993924	3.242688	9.44E-06	4.77E-05
AL359881	0.11211	0.506897	2.176779	0.000205	0.000644
DLEU7	0.079032	0.342788	2.116811	1.93E-12	1.40E-09
MIR25	1.124357	3.431506	1.609742	3.28E-07	2.92E-06
AQP2	0.030273	0.97827	5.014131	0.001821	0.004227
AC087269	0.084481	0.533015	2.657475	3.86E-06	2.25E-05
CYP2B6	1.725835	5.343821	1.630577	2.84E-05	0.00012
AC013731	0.119671	0.384654	1.684494	3.04E-06	1.84E-05
CPN1	0.013052	0.332726	4.671974	0.000963	0.002442
RN7SL505	0.066425	0.239833	1.852224	7.16E-06	3.77E-05
RNY4P19	0.076097	0.401876	2.400838	0.000826	0.002139
AC007342	0.347506	1.531517	2.139852	4.48E-06	2.56E-05
SLC5A2	0.075115	0.267878	1.834409	8.91E-08	1.02E-06
AC090673	0.038168	0.340755	3.158285	5.64E-10	1.95E-08
TNNI3	0.229837	0.844716	1.877857	9.75E-06	4.90E-05
SHANK2-	0.060173	0.262925	2.12747	3.24E-05	0.000134
IGKV2-4	0.948666	0.197734	-2.26234	0.011255	0.020416
PCAT1	0.062255	0.271371	2.123998	2.50E-08	3.63E-07
RN7SL541	0.05858	0.393901	2.74935	2.96E-08	4.19E-07
MIR579	0.08096	0.379073	2.227186	4.01E-05	0.000162
AC019227	0.19122	0.554681	1.536425	0.000518	0.001425
ZFP36	508.7434	175.021	-1.53941	3.36E-05	0.000138
CCL20	13.58526	49.59117	1.868041	0.003496	0.007441
KIF18A	0.659115	3.013702	2.192935	4.17E-11	3.74E-09
MAMDC2	11.80861	1.588056	-2.89451	2.08E-09	5.17E-08
LIMS2	15.40251	4.59363	-1.74546	0.000166	0.000535
AC124067	0.152924	1.301434	3.089214	1.47E-05	6.89E-05
DPT	64.33818	5.348179	-3.58856	8.53E-12	2.04E-09
SLC22A1	0.084968	0.264856	1.640208	7.19E-05	0.000262
AC073957	0.31106	1.152581	1.889605	4.37E-09	9.16E-08
STMN2	4.263079	1.189122	-1.842	0.002502	0.005564
PTN	18.63078	5.014096	-1.89363	2.11E-07	2.05E-06

MIR5582	0.106509	0.301841	1.502817	0.026905	0.043449
IGKV2D-2	1.416957	0.192317	-2.88124	0.012345	0.022073
LRRC15	0.95127	3.007139	1.660465	0.000154	0.000503
RNU6-116	0.055021	0.320989	2.54448	0.000175	0.000561
AMTN	1.468623	0.289979	-2.34044	5.31E-05	0.000204
MT1A	11.46396	3.68711	-1.63654	2.75E-08	3.94E-07
RNU6-479	0.078731	0.228526	1.537347	0.013167	0.023381
AC090709	0.021424	0.349152	4.026543	0.000118	0.000401
DUSP26	1.357639	0.472552	-1.52255	1.26E-05	6.06E-05
UHRF1	1.249836	5.419974	2.116547	3.22E-11	3.24E-09
MIR548AC	0.094826	0.499495	2.397111	0.001193	0.002939
LINC0197	0.062183	0.343811	2.467035	9.86E-06	4.94E-05
MIR200C	0.057035	0.242598	2.08864	0.001499	0.003581
RNU6-115	0.259882	0.906056	1.801745	2.11E-06	1.36E-05
AC108463	0.071598	0.354187	2.306529	1.35E-10	7.40E-09
AC087222	0.205617	0.608584	1.565498	6.07E-06	3.30E-05
PRR7	2.658314	9.294197	1.805819	3.23E-10	1.30E-08
RPL21P65	0.06773	0.223522	1.722538	2.36E-05	0.000103
MIR1245A	0.104992	0.532202	2.341698	0.000837	0.002163
LINC0232	0.073138	0.3316	2.180752	0.000245	0.00075
USP32P3	0.064504	0.22753	1.818609	5.93E-06	3.24E-05
MIR4635	0.458884	2.03161	2.146423	1.64E-06	1.09E-05
SNORD7	0.218959	0.982807	2.166246	5.58E-07	4.49E-06
MIR4753	0.089806	0.345788	1.945006	0.010643	0.019442
HBA1	12.07828	2.540965	-2.24897	2.05E-09	5.11E-08
ATP4B	304.7412	10.49374	-4.85998	0.001664	0.00391
TPM2	245.9354	73.90878	-1.73446	0.002367	0.005301
MIR4261	0.166492	1.103014	2.727928	3.07E-06	1.85E-05
AL353705.	0.113621	0.339964	1.581153	0.002338	0.005245
ARL4AP5	0.07448	0.251322	1.754611	1.18E-07	1.28E-06
AP003108	0.027032	0.221712	3.035923	0.00713	0.013775
LINC0216	0.009645	0.9301	6.591412	2.92E-10	1.21E-08
FOXE3	0.018277	0.245694	3.748768	8.84E-09	1.61E-07
AP001542	0.062382	0.486373	2.962867	2.61E-06	1.62E-05
KIF24	0.63124	2.04136	1.693271	1.21E-10	6.81E-09
RUNX1	2.61365	8.496407	1.700787	1.79E-11	2.51E-09
ZNF812P	5.231531	0.535398	-3.28855	0.000843	0.002174
CHEK1	1.19334	3.725808	1.642548	3.44E-11	3.31E-09
DNAH14	0.069233	0.296797	2.099953	0.000223	0.00069
MIR589	0.296471	0.994012	1.745371	3.15E-06	1.89E-05
LINC0208	0.300048	2.258119	2.911858	3.43E-07	3.01E-06
AL136982.	0.089093	0.269909	1.599095	1.05E-05	5.23E-05
IGKV1OR2	7.183942	0.840853	-3.09485	0.021881	0.036348
THBS2	6.168096	28.70828	2.21857	6.02E-08	7.39E-07
AL121721.	0.017088	0.282508	4.047215	3.67E-05	0.00015
AC005086	0.300407	0.857307	1.512892	0.001225	0.003008
MIR3945H	0.129976	0.556082	2.097048	1.20E-05	5.83E-05
CST5	0.051006	0.248037	2.281823	0.000356	0.001032
AC010487	0.18028	0.657087	1.86585	2.06E-06	1.33E-05
RNA5SP25	0.068632	0.274981	2.002385	0.006506	0.01273
RF02246	0.029178	0.825786	4.822796	9.11E-05	0.000321
MCM4	6.456335	18.70196	1.534402	2.83E-09	6.55E-08
HMGN1P1	0.074925	0.221524	1.563939	0.000395	0.001128
AL035458.	0.202102	0.676322	1.742627	5.98E-07	4.75E-06
AC004233	0.054528	0.267117	2.292392	0.000453	0.00127
GPC2	0.079951	0.321928	2.009551	6.04E-06	3.28E-05
RNU6-106	0.084213	0.285461	1.761181	0.007942	0.015125
ARL9	0.418095	1.244685	1.573877	9.64E-05	0.000336

PF4V1	0.095291	0.359416	1.915234	0.001862	0.004311
SNORD12	0.607266	3.672704	2.596442	8.16E-11	5.47E-09
ZFP42	0.011385	0.301607	4.727408	0.004035	0.008427
AL391832.	0.253639	0.87864	1.792497	8.91E-07	6.63E-06
BRCA1	1.184962	3.51892	1.570292	1.10E-10	6.51E-09
AL356320.	0.160037	0.555904	1.796427	6.51E-07	5.10E-06
AC018641	0.01366	0.273161	4.321695	7.60E-08	8.92E-07
SOX21-AS	4.642353	1.349135	-1.78282	0.000627	0.001679
SALL4	0.061807	2.200915	5.154187	3.27E-12	1.40E-09
LARP1P1	0.05848	0.243341	2.056959	2.45E-05	0.000106
AC013652	0.078112	0.330279	2.080061	1.96E-05	8.75E-05
AC011773	0.093527	0.346257	1.888388	7.57E-07	5.81E-06
RNU1-72P	0.03192	0.229264	2.844479	0.000428	0.00121
RNA5SP33	0.124912	0.370552	1.56876	0.005692	0.011341
F2RL2	1.491704	6.953279	2.220732	4.77E-09	9.85E-08
SCNN1B	13.43332	1.055161	-3.67028	1.19E-10	6.77E-09
PARPBP	0.973073	2.940527	1.595454	2.14E-09	5.28E-08
LINC0095	0.097926	0.382232	1.96469	0.001595	0.00377
PPP1R14A	34.47669	10.66932	-1.69215	4.60E-06	2.61E-05
RNU4-25P	0.053532	0.230517	2.106389	0.00036	0.001041
PVR	8.74607	25.18279	1.525731	7.08E-11	5.00E-09
RNU2-22P	0.1145	0.370606	1.694538	0.002276	0.005123
SLC17A9	2.206332	9.844993	2.15774	2.71E-08	3.89E-07
DACT3	9.599488	3.168492	-1.59916	0.000151	0.000495
AC015883	0.163837	0.584388	1.834662	7.88E-07	5.99E-06
FRGCA	0.02841	0.25849	3.18565	0.000951	0.002414
SNORA9B	0.130844	0.609224	2.21912	1.28E-07	1.36E-06
DDX50P1	0.083864	0.249793	1.574603	1.25E-08	2.10E-07
UPK3A	0.259267	3.654198	3.817044	0.027505	0.044321
SOHLH1	0.005069	0.414577	6.353694	0.000329	0.000964
AC233280	0.146922	0.656093	2.15885	1.13E-07	1.24E-06
AL163953.	0.075453	0.438352	2.538435	0.000282	0.000844
TMEM74B	0.84007	3.065274	1.867435	7.91E-05	0.000284
IGKV2-40	2.486435	0.449821	-2.46666	0.019841	0.033378
AC108451	0.307732	1.026782	1.738382	0.004181	0.008688
ATP2A1	0.159196	0.493154	1.631232	1.18E-07	1.28E-06
RFX8	0.039824	0.218677	2.457095	2.57E-08	3.72E-07
GTF2IP9	0.288504	0.821875	1.510326	4.92E-06	2.76E-05
CATIP-AS	0.11928	0.480866	2.011284	6.39E-07	5.02E-06
APOA4	495.4121	8.475145	-5.86925	0.009864	0.018238
AL035456.	0.116532	0.379024	1.701562	4.00E-10	1.52E-08
AL117381.	0.236058	0.882139	1.901865	4.41E-07	3.69E-06
MIR548I2	0.185723	0.795267	2.098289	4.25E-05	0.00017
LBP	0.076918	1.965733	4.675593	0.000773	0.002014
ZNF695	0.096426	0.648019	2.748537	3.71E-10	1.43E-08
AC122688	0.225682	0.643308	1.511217	3.07E-08	4.31E-07
AC010343	0.012991	0.279795	4.42877	0.002059	0.004701
KRT23	0.421981	14.67923	5.120454	6.87E-05	0.000253
MUC12	0.204891	1.725131	3.073781	3.20E-05	0.000133
RAD51AP1	1.458185	6.195384	2.08702	1.71E-11	2.48E-09
RNU6-554	0.079447	0.284797	1.841868	0.000582	0.001578
TSPEAR	0.072803	0.414208	2.508294	3.17E-05	0.000132
AC105219	0.076927	0.621822	3.014937	1.41E-08	2.31E-07
AC073657	0.20061	0.611556	1.60809	6.68E-05	0.000247
CYCSP6	0.165226	1.051589	2.670062	0.000358	0.001037
TRIP13	1.405273	6.954613	2.30712	2.61E-11	2.91E-09
PRKCG	0.409128	1.818149	2.151847	4.65E-06	2.63E-05
AC087620	0.216928	0.634807	1.549104	0.000205	0.000642

CNTN1	4.610052	1.015943	-2.18196	2.37E-05	0.000103
LINCR-00(	0.108764	0.808474	2.894003	2.73E-06	1.69E-05
FCGR1A	0.412078	1.545693	1.907263	2.21E-09	5.41E-08
NCR3LG1	0.800907	3.024966	1.917213	1.35E-07	1.42E-06
WSCD2	2.776643	0.714847	-1.95763	1.76E-05	8.00E-05
GCNT4	6.690099	1.457384	-2.19865	1.08E-07	1.19E-06
AC103740	0.737456	0.183284	-2.00847	0.013636	0.0241
LINC0088	0.281593	0.842464	1.581003	2.43E-06	1.53E-05
BX470102	0.836459	2.79847	1.742272	1.04E-06	7.60E-06
PROX1	0.534017	3.416248	2.677456	0.022299	0.036936
PRC1-AS1	0.08585	0.278387	1.697204	1.45E-07	1.51E-06
MIR548AA	0.178553	0.968063	2.438752	9.36E-07	6.92E-06
U47924.1	0.067097	0.243334	1.858612	1.11E-05	5.43E-05
BPIFA2	0.017814	0.842084	5.562841	1.32E-05	6.29E-05
DNAJB5	11.90185	3.472122	-1.7773	6.87E-05	0.000253
KIF23	1.14474	5.156333	2.171325	3.12E-11	3.15E-09
LHX4	0.113989	0.330221	1.534532	3.42E-09	7.54E-08
AP000943	0.008109	0.319074	5.298234	2.97E-06	1.80E-05
VEGFD	4.127582	0.644971	-2.67799	1.21E-10	6.81E-09
TPSP2	0.012822	0.267796	4.384409	1.62E-05	7.46E-05
LRGUK	0.064438	0.256007	1.990202	6.97E-07	5.41E-06
COX7A1	18.72902	6.323361	-1.56651	1.55E-06	1.05E-05
FAM19A4	6.570484	0.393004	-4.06338	0.00013	0.000436
LINC0039	0.018088	0.352763	4.285612	0.025245	0.041127
AC024940	0.150541	0.426803	1.503415	1.41E-06	9.68E-06
KCNA5	3.356857	0.543555	-2.62661	2.19E-06	1.40E-05
TMEM35A	5.727978	0.874889	-2.71085	9.94E-07	7.29E-06
AP000919	0.124354	0.359275	1.530634	1.26E-07	1.35E-06
IGKV1OR9	2.951802	0.272386	-3.43787	0.005273	0.010627
LYVE1	10.28962	1.420513	-2.85671	4.39E-10	1.64E-08
CLDN9	0.134419	1.299094	3.272695	1.78E-05	8.06E-05
AC019117	0.205701	1.370549	2.736133	0.000263	0.000799
RN7SL233	0.124039	0.574202	2.210768	9.45E-06	4.77E-05
MTND4P2	0.046797	0.252929	2.434244	2.81E-09	6.53E-08
PGR	0.841656	0.259352	-1.69832	0.000622	0.001667
AC012531	0.006864	0.437603	5.994455	1.47E-06	1.00E-05
ATP6V1G2	0.932586	0.294	-1.66542	0.003338	0.007137
AC023302	0.081037	0.241178	1.573438	0.000146	0.000481
AC253536	0.173673	1.167514	2.748996	0.014426	0.025319
MSL3P1	0.167754	0.482891	1.525348	3.18E-06	1.90E-05
ATP6V0D2	0.205947	0.651412	1.661297	7.71E-05	0.000278
LINC0183	0.302655	1.168236	1.948583	1.25E-06	8.78E-06
AL354993.	0.100994	0.911075	3.173303	4.91E-05	0.000191
AC004264	0.188759	0.916168	2.279068	7.02E-10	2.31E-08
AC127024	0.11632	0.384822	1.726089	2.32E-08	3.44E-07
FAM86GP	0.136402	0.433921	1.669571	3.48E-05	0.000143
ACAP2-IT	0.216164	0.612918	1.503565	1.56E-06	1.05E-05
AC022080	0.067745	0.219037	1.692977	0.000662	0.001761
BBIP1P1	0.105754	0.354155	1.743666	2.89E-05	0.000122
DUXAP8	0.040162	0.54918	3.773376	1.07E-10	6.36E-09
COX6B2	0.043632	0.547041	3.648202	6.21E-09	1.22E-07
KRTAP5-1	0.226447	0.821286	1.85871	2.14E-07	2.06E-06
RNU6-19F	0.061231	0.221375	1.854164	0.001008	0.002537
FAM3B	21.75096	7.650915	-1.50737	0.001	0.002519
TBC1D30	0.685979	2.116326	1.625325	2.46E-08	3.59E-07
MIR181A2	0.037853	0.243271	2.684097	9.51E-05	0.000333
C2CD4A	0.923466	5.835445	2.659712	3.22E-06	1.92E-05
PRR7-AS1	0.157435	0.65118	2.048296	5.95E-10	2.03E-08

P2RY1	1.933146	0.642721	-1.58869	0.013636	0.0241
TTLL2	0.039688	0.222697	2.488325	1.27E-05	6.11E-05
MIR3648-	0.07654	0.438571	2.518524	0.015063	0.026271
RPL36P16	0.109842	0.497916	2.180477	2.44E-07	2.28E-06
RNU6-547	0.186959	0.655939	1.810839	2.13E-05	9.41E-05
COCH	0.378837	1.969397	2.378105	0.000183	0.000582
LINC0208:	0.079162	0.436375	2.462689	6.83E-08	8.18E-07
EIF1P4	0.072782	0.221486	1.605569	0.005769	0.011478
MIR554	0.171476	0.950474	2.470637	5.62E-07	4.51E-06
AC020656	0.253162	1.63077	2.68742	1.21E-06	8.53E-06
ZNF469	0.335447	1.769015	2.398791	9.57E-11	5.99E-09
AC010536	0.081951	0.255931	1.64292	1.77E-08	2.76E-07
AL606489.	0.07253	0.295524	2.026627	9.01E-06	4.58E-05
AC007639	0.096754	0.384035	1.988852	5.05E-07	4.14E-06
ARHGEF26	7.511462	2.293838	-1.71133	0.000391	0.001118
DBF4	1.645681	5.628122	1.773969	1.45E-11	2.41E-09
LRRC37A6	0.133484	0.39014	1.547327	0.002771	0.006078
DCDC2	0.154209	1.271299	3.04335	0.000121	0.000408
AL162727.	0.071476	0.347506	2.281509	1.52E-08	2.44E-07
AC020922	0.117829	0.424675	1.849665	7.88E-07	5.99E-06
METTL24	4.908335	1.211123	-2.01889	2.72E-05	0.000116
TRPM2	0.963127	2.868329	1.574412	1.46E-08	2.36E-07
MIR4312	0.126338	0.553476	2.131233	4.14E-05	0.000166
PGM5P4	2.810631	0.741841	-1.92171	1.67E-05	7.65E-05
RNU6-640	0.081713	0.36171	2.146194	0.001893	0.004374
AC026356	0.172856	0.544969	1.656603	4.79E-08	6.17E-07
EMP1	139.7881	18.19776	-2.94141	0.025255	0.041127
CENPK	0.833244	2.574217	1.627322	3.00E-09	6.87E-08
SH3BGR	7.43124	1.80416	-2.04228	3.29E-05	0.000136
AP000943	0.120889	0.432668	1.839571	4.01E-06	2.33E-05
RNU6-302	0.054851	0.366312	2.739483	1.12E-05	5.51E-05
RPL7AP28	0.598739	0.175114	-1.77363	0.000174	0.000558
HOXC-AS	0.054559	0.53736	3.299998	2.09E-11	2.71E-09
RNU4-38F	0.077638	0.564938	2.863249	1.79E-05	8.12E-05
AC091588	0.164975	0.653132	1.985132	3.45E-06	2.04E-05
AL139246.	0.076804	0.246649	1.683211	1.54E-05	7.15E-05
LINC0191	0.031259	0.418947	3.744411	9.83E-11	6.05E-09
AC022973	0.082966	0.292714	1.818904	1.57E-07	1.61E-06
AC021739	0.073853	0.23133	1.647218	1.18E-05	5.73E-05
AC016526	0.075262	0.282044	1.905916	5.44E-06	3.02E-05
KCNS1	0.033918	0.253979	2.904593	0.007042	0.013627
ASCL5	0.063123	0.260281	2.043844	0.000152	0.000499
VTRNA1-3	1.709935	0.537699	-1.66907	0.018833	0.031871
ACTBL2	0.055893	0.226835	2.02091	2.52E-06	1.58E-05
R3HDML	0.032939	0.333019	3.337752	2.58E-08	3.74E-07
MAGEA1	0.010208	2.107425	7.689617	0.007988	0.015198
GAPDHP6	0.044928	0.217165	2.273103	1.96E-09	4.94E-08
CCDC162I	0.059198	0.405553	2.776268	3.96E-07	3.39E-06
IQCH	0.138671	0.412964	1.57435	3.25E-06	1.94E-05
AC026740	0.358714	1.603537	2.16035	3.51E-08	4.81E-07
RNU6-238	0.218199	0.840026	1.944788	2.96E-05	0.000124
AC073333	0.171409	0.62734	1.871808	3.57E-10	1.40E-08
AC015660	0.075456	0.306599	2.022649	0.007513	0.014416
AL035461.	0.61719	2.237496	1.858098	5.99E-09	1.18E-07
SNORC	0.803951	3.022677	1.910647	1.40E-05	6.61E-05
ARC	8.593914	0.794916	-3.43444	7.67E-05	0.000277
AC004009	0.079818	0.309921	1.957114	0.015113	0.02635
LINC0123	0.024922	0.968801	5.280685	6.84E-09	1.31E-07

AKR1B10P	4.745367	0.673053	-2.81773	0.00022	0.000682
HMGA2	0.062111	1.619224	4.704305	1.94E-10	9.26E-09
ELFN1-AS	0.737229	5.243797	2.830427	1.90E-06	1.24E-05
FANCB	0.189997	0.810601	2.093018	1.40E-11	2.41E-09
MACC1	2.771387	8.7238	1.654348	1.93E-07	1.91E-06
GSN	116.4378	39.50002	-1.55963	5.50E-09	1.11E-07
SLC22A20	0.147672	0.425565	1.526986	0.00065	0.001732
AC211476	0.132423	0.391605	1.564245	3.32E-07	2.95E-06
IGHVIII-67	4.556258	0.688012	-2.72734	0.022162	0.036743
DEPDC1	1.034378	3.576599	1.789824	6.25E-09	1.22E-07
URB2	1.349014	3.909378	1.535034	3.38E-12	1.40E-09
CACNA1E	0.031906	0.317101	3.313027	2.45E-05	0.000106
LDHAL6B	0.079323	0.230571	1.539407	1.08E-06	7.80E-06
AC023043	0.483574	1.547185	1.677837	3.86E-08	5.19E-07
ASPHD1	2.31	7.88314	1.770877	0.000137	0.000455
NR6A1	0.360443	1.111653	1.624864	1.61E-07	1.65E-06
OLFML2B	4.225746	14.32205	1.76096	4.79E-08	6.17E-07
AC090772	0.08091	0.248879	1.621052	1.51E-05	7.03E-05
C6orf223	0.362594	3.795019	3.387681	1.20E-07	1.29E-06
SLC12A8	1.509231	4.408204	1.546377	1.14E-10	6.59E-09
SLC19A3	0.83072	3.20653	1.948578	0.005858	0.011617
GIF	240.7957	11.15939	-4.43148	0.022474	0.037186
GIP	25.6534	0.605382	-5.40516	0.000163	0.000526
APLN	0.466807	3.148749	2.75388	2.41E-09	5.81E-08
PRSS41	0.099308	0.798648	3.00758	0.00116	0.002868
C11orf96	84.07809	28.1814	-1.57699	0.003262	0.006995
SYT13	2.498214	12.25196	2.294043	0.000112	0.000382
AP005271	0.040616	0.283886	2.805205	0.018227	0.030985
LIF	1.508628	7.601252	2.333	2.23E-10	1.02E-08
AP000553	0.066088	0.219921	1.734517	0.000202	0.000635
RN7SKP18	0.075854	0.225588	1.572384	5.50E-05	0.00021
AC093458	0.066678	0.213048	1.675907	0.002023	0.004631
CACNB2	2.862834	0.895698	-1.67636	0.011841	0.021302
ZIC2	0.210243	2.589844	3.622737	2.69E-05	0.000115
AC068587	0.723334	2.110497	1.544849	0.007222	0.013923
AC090181	0.192074	0.599152	1.641259	4.25E-07	3.59E-06
WASIR2	0.105758	0.34207	1.693518	0.000215	0.000668
AL353807.	0.435629	1.591361	1.86909	3.68E-08	4.99E-07
NFE2L3	4.780891	18.10456	1.921002	2.08E-11	2.71E-09
ATP6V1B1	0.108152	0.738969	2.772458	1.26E-05	6.09E-05
AC104564	0.070887	0.222898	1.652799	5.03E-06	2.82E-05
ASPA	1.342537	0.352711	-1.9284	1.60E-09	4.25E-08
FANCD2	0.821007	2.763791	1.751182	1.49E-10	7.84E-09
SCUBE1	2.166236	0.696114	-1.63779	4.92E-05	0.000191
SLC28A1	1.648175	0.095664	-4.10675	0.003945	0.008263
LINC0232	0.104635	0.557012	2.412347	2.19E-06	1.40E-05
CHRM2	4.844908	0.926727	-2.38625	0.000753	0.00197
PPP1R1A	11.46719	1.034345	-3.47072	4.30E-07	3.62E-06
Z94721.1	0.318215	1.05852	1.733976	8.73E-09	1.60E-07
MIR6739	0.496841	1.57642	1.665795	2.20E-06	1.40E-05
HNRNPA3	0.138754	0.512017	1.883665	4.88E-10	1.76E-08
RN7SL442	0.057805	0.212651	1.879219	0.025337	0.04125
RNA5SP10	0.162173	0.501103	1.627574	0.000217	0.000675
ALG1L3P	0.032357	0.297828	3.202313	1.09E-05	5.38E-05
IGKV1OR2	5.01171	0.636195	-2.97776	0.014325	0.025168
CASC9	0.465332	3.544292	2.929164	1.57E-06	1.06E-05
MIR624	0.057369	0.292602	2.350594	0.002375	0.005318
AL136980.	0.065573	0.233926	1.834886	4.76E-05	0.000186

AL451050.	0.195044	0.659053	1.756594	5.08E-09	1.04E-07
AL049612.	0.088176	0.31829	1.851889	0.013975	0.024626
AC097493	0.162355	0.594916	1.873534	1.10E-05	5.40E-05
APOC1	8.096319	39.32638	2.280159	2.83E-08	4.02E-07
KRTAP4-1	0.004945	0.588311	6.894366	2.36E-06	1.49E-05
MMP13	0.031622	0.854844	4.75666	5.91E-09	1.18E-07
SNORA1B	0.13817	0.460443	1.736576	0.000272	0.000821
RECQL4	2.291267	9.578913	2.063717	1.71E-10	8.37E-09
AC092535	0.56557	2.032593	1.845545	1.91E-06	1.25E-05
ERBB2	16.21466	61.58727	1.925333	0.00815	0.015469
KLF2P1	0.011525	0.65945	5.838417	7.76E-08	9.08E-07
C5orf66-A	9.342769	1.04896	-3.15489	5.19E-09	1.06E-07
GJB4	0.256617	1.398048	2.445728	5.84E-06	3.20E-05
FOXC1	2.731533	8.950558	1.712266	8.08E-06	4.18E-05
GAS8-AS1	0.118716	0.396494	1.739778	6.55E-06	3.51E-05
IGLV8OR8	1.23666	0.178331	-2.79382	0.027718	0.044596
AC226118	0.080264	0.381575	2.249141	0.005073	0.010263
ZNF525	0.548155	1.621459	1.564635	1.61E-07	1.65E-06
EME1	0.308283	1.429965	2.213651	2.33E-11	2.88E-09
LINC0094	0.124459	1.410855	3.502828	1.27E-06	8.87E-06
LINC0141	0.005819	0.335265	5.848323	0.002575	0.005708
SKA1	1.049555	3.82171	1.86444	1.20E-09	3.43E-08
WASHC5-	0.076006	0.373843	2.29824	7.07E-10	2.32E-08
AC124947	0.347583	1.491404	2.101243	1.08E-07	1.19E-06
GINS4	0.475929	1.600555	1.749754	3.60E-10	1.40E-08
AL034550.	0.198829	0.773335	1.959567	4.19E-09	8.91E-08
GAPLINC	0.15826	0.689193	2.122613	8.67E-10	2.70E-08
AL606490.	0.064824	0.222642	1.780116	0.001506	0.003592
PLIN4	23.15584	4.47899	-2.37013	1.24E-06	8.69E-06
FKBP10	6.546421	37.73252	2.52703	2.06E-07	2.01E-06
CDH3	2.877836	8.76555	1.60686	5.45E-07	4.40E-06
CHRNA1	0.047674	0.480166	3.332262	3.91E-11	3.62E-09
RNA5SP3(	0.069834	0.242423	1.795519	0.001378	0.003329
CLCN5	0.894835	2.549735	1.510653	3.42E-09	7.54E-08
KCNC3	0.348005	1.254002	1.84936	1.21E-07	1.31E-06
OVOL3	0.077296	0.265563	1.780592	2.43E-06	1.53E-05
AC004801	0.172791	0.63046	1.86738	3.07E-08	4.31E-07
LINC0116	0.18793	0.60317	1.682371	0.011941	0.021462
NXPH4	0.426795	3.391684	2.990386	1.70E-05	7.79E-05
DES	1352.684	426.9645	-1.66364	5.97E-06	3.25E-05
TMPRSS1	21.02437	0.996664	-4.39881	0.013843	0.024414
AL139349.	0.223822	0.645607	1.528302	9.76E-06	4.90E-05
CCDC148	0.092322	0.264978	1.521119	0.00046	0.001286
NECAB1	2.154687	0.512939	-2.07062	0.000409	0.001162
UNC93B2	0.06291	0.229089	1.864539	0.000459	0.001284
MIR194-1	0.080339	0.320322	1.995344	0.001284	0.003135
LINC0128	0.008863	0.394228	5.475114	0.015091	0.026315
C1orf195	0.237765	0.788625	1.729804	1.82E-07	1.82E-06
ULBP3	0.774111	2.2362	1.530437	6.15E-05	0.00023
MEX3A	0.25489	2.213447	3.118349	1.47E-11	2.41E-09
PLAU	5.86371	27.41512	2.225086	1.29E-10	7.14E-09
CBX2	0.495631	2.788587	2.492197	7.74E-09	1.45E-07
ADH7	16.20479	0.715961	-4.5004	1.07E-08	1.86E-07
APOC1P1	0.085376	0.390175	2.192216	3.14E-06	1.89E-05
MIR1285-	0.490225	1.417219	1.531546	0.000575	0.00156
AP005233	0.360853	2.472484	2.776477	4.29E-07	3.62E-06
RNU6-93	0.099791	0.485632	2.282877	0.000289	0.000863
AC068491	0.041435	0.274311	2.726889	1.16E-10	6.61E-09

CSAG1	0.058083	6.652679	6.839686	0.000208	0.000652
AC087752	0.178656	0.51775	1.53507	4.62E-05	0.000182
MNX1-AS	0.284585	0.888463	1.642452	1.08E-06	7.79E-06
AP002336	0.223472	0.646591	1.532761	4.22E-07	3.57E-06
RNU6-48I	0.134242	0.389881	1.538201	0.000139	0.00046
AC100843	0.060316	0.244299	2.018039	2.09E-05	9.26E-05
BARX1	67.12498	19.23225	-1.80332	0.000564	0.001534
TREM2	2.004752	9.404202	2.229882	4.73E-10	1.72E-08
CYP2C18	18.42123	6.206749	-1.56946	0.003889	0.008158
AC244093	0.055856	0.262276	2.231292	1.05E-08	1.84E-07
CEP55	3.078272	11.80918	1.939716	1.63E-10	8.20E-09
IER5L	5.171814	16.48174	1.672126	2.87E-09	6.62E-08
AC005162	0.075118	0.252064	1.746571	0.016189	0.027978
CACNA1D	0.277392	1.179786	2.088526	7.45E-08	8.75E-07
SLC9A4	9.183814	1.946467	-2.23824	0.000934	0.002377
AL359636.	0.073777	0.286904	1.959336	0.002157	0.004899
TCEAL5	0.549377	0.174998	-1.65046	1.60E-06	1.08E-05
MAP6	2.668562	0.884037	-1.59388	0.000247	0.000755
ARHGAP3	1.744366	5.287523	1.599889	2.94E-10	1.22E-08
FGF19	0.009225	2.492638	8.077888	1.25E-07	1.34E-06
AC012254	0.054562	0.258416	2.243725	0.000452	0.001268
HMGN2P2	0.119457	0.373858	1.646	1.51E-06	1.02E-05
PRSS56	0.004493	0.713272	7.310767	0.001372	0.003316
AC078778	0.211731	0.822364	1.957548	2.68E-08	3.85E-07
BGN	47.80769	234.5953	2.29486	5.31E-11	4.26E-09
ELOVL4	2.352988	0.579375	-2.02193	0.007432	0.014277
MIR1972-	0.276691	0.935455	1.757391	8.42E-05	0.0003
AP001065	0.047235	0.230451	2.28652	0.000244	0.000747
MIR4783	0.064397	0.293673	2.189135	0.008962	0.016777
AC093520	0.089045	0.35007	1.975043	9.88E-07	7.26E-06
COL9A1	0.196207	0.899961	2.197483	0.000281	0.000842
HSPB3	1.477387	0.240902	-2.61653	9.35E-05	0.000328
PODXL2	8.264187	23.47033	1.505893	0.000132	0.00044
RF02141	0.051625	0.753071	3.866654	2.07E-07	2.01E-06
ADAMTSL	1.741388	0.567718	-1.61699	1.05E-07	1.17E-06
AC012254	0.093741	0.306298	1.708184	0.004266	0.008836
COL22A1	0.11575	1.218327	3.395821	2.40E-08	3.51E-07
RNU6-757	0.177484	0.529075	1.575785	0.001745	0.004071
AL158839.	0.076679	0.322038	2.070325	0.010695	0.019523
AC002128	0.270937	0.848813	1.64749	1.87E-07	1.86E-06
NCAM1	4.924092	0.988486	-2.31657	3.47E-07	3.04E-06
MORN3	0.178029	0.602716	1.75937	0.003496	0.007441
CST1	3.052795	91.62547	4.907546	2.74E-12	1.40E-09
OTOP3	10.40855	0.482043	-4.43246	0.000361	0.001042
LOXL2	4.616483	13.10606	1.505368	3.23E-09	7.25E-08
LEFTY1	0.240377	6.431912	4.741878	0.00359	0.007613
CYP24A1	0.04541	0.928539	4.353869	0.002347	0.005262
TNFSF15	1.0076	3.098271	1.62054	1.61E-07	1.65E-06
NARF-AS1	0.068569	0.235545	1.780381	1.08E-07	1.19E-06
HOXC-AS	0.028789	0.342614	3.572973	4.74E-09	9.80E-08
PKMYT1	1.617563	5.409506	1.741675	1.80E-09	4.63E-08
FSCN1	9.77752	30.4042	1.63673	1.81E-06	1.20E-05
MCTS2P	1.110028	3.482409	1.64949	7.69E-10	2.48E-08
AC107308	0	0.28733	Inf	2.70E-08	3.88E-07
AC024361	0.120708	0.384273	1.670614	3.97E-05	0.00016
AL138760.	0.074301	0.218995	1.559448	0.004256	0.008822
MYH11	600.8356	134.134	-2.1633	8.83E-06	4.50E-05
TRIM71	0.007174	0.236837	5.045006	0.002017	0.00462

AC006042	0.570543	2.528542	2.147899	1.77E-08	2.76E-07
AC011825	0.109522	0.320178	1.547653	1.94E-06	1.26E-05
AC007249	0.143271	0.48009	1.744553	3.36E-05	0.000138
DLG2	0.545494	0.183177	-1.57433	0.001204	0.002959
AL356215.	0.071537	0.297919	2.05815	0.001815	0.004217
DKK2	0.312159	0.965653	1.629225	2.16E-05	9.51E-05
NOX1	0.223876	4.702112	4.392538	1.38E-07	1.45E-06
SEMG2	0.020346	0.291279	3.839565	0.00804	0.01528
AL080317.	0.916463	2.600833	1.504826	1.46E-09	3.96E-08
AC011447	0.434522	1.807089	2.056165	0.001245	0.003048
RDH8	0.010472	0.493545	5.558543	6.89E-08	8.22E-07
HOXB-AS	0.304176	1.685206	2.469948	6.53E-08	7.89E-07
AC106028	0.102665	0.330243	1.685582	2.93E-07	2.65E-06
CTD-2201	0.106795	0.337755	1.661129	5.31E-05	0.000204
IGKV2OR2	16.79962	1.981209	-3.08398	0.018316	0.031107
MMP1	24.08508	73.51541	1.609907	4.67E-05	0.000183
RPL23AP5	0.139193	0.434482	1.642211	3.13E-08	4.39E-07
AP000345	0.061189	0.214937	1.812581	0.000626	0.001676
EZH2	2.105213	6.223342	1.563723	1.34E-09	3.71E-08
RDH16	0.071697	0.295711	2.044214	4.09E-05	0.000164
BARX1-DT	1.222905	0.405905	-1.5911	0.001809	0.004205
AC131097	0.035375	0.45234	3.676614	1.66E-08	2.62E-07
AL356299.	0.08535	0.462244	2.437185	1.01E-09	2.99E-08
AC073592	0.044786	0.251823	2.491283	0.000132	0.000441
AL592295.	0.11447	0.418826	1.871376	1.81E-05	8.17E-05
PRR20G	0.020283	0.956685	5.559735	0.00553	0.011067
C6orf15	0.019406	2.288063	6.881453	0.000259	0.000786
AC083906	0.042132	0.273139	2.696652	2.90E-06	1.77E-05
ADAMTS2	2.005901	10.71008	2.416647	5.06E-11	4.13E-09
MEST	2.768328	13.16557	2.249684	1.25E-10	6.96E-09
AC053503	2.656956	0.718069	-1.88758	0.000647	0.001727
RPL7P57	0.069712	0.25302	1.859764	4.07E-08	5.43E-07
MIR581	0.135426	0.490165	1.855767	0.001793	0.004171
SETP5	0.084404	0.262959	1.639455	5.63E-06	3.11E-05
CKMT2	3.366966	0.674179	-2.32025	1.67E-08	2.63E-07
REEP2	4.255688	1.498325	-1.50604	0.001387	0.003345
JPH2	13.47813	4.110679	-1.71317	0.000303	0.000898
HOXC8	0.078109	1.613647	4.36869	1.15E-11	2.25E-09
SYCP2	0.226439	0.742519	1.713304	0.029601	0.047167
DENND2C	2.506303	0.544176	-2.20342	0.014766	0.025828
ELFN2	0.033427	0.288786	3.110902	0.000161	0.000521
ANGPTL7	2.138054	0.414511	-2.36682	6.54E-05	0.000242
ECEL1	0.25696	1.343092	2.385944	4.65E-05	0.000182
COL1A1	71.6203	465.2526	2.699574	1.02E-11	2.17E-09
AC007272	0.120129	0.35011	1.543222	2.29E-05	1.00E-04
AC079062	0.036292	0.443782	3.612127	0.003736	0.007884
RPLP0P2	0.301639	1.436044	2.251204	8.98E-11	5.79E-09
RNU6-82F	0.07475	0.230946	1.627406	0.003644	0.007714
AP003555	0.300086	1.592494	2.40784	3.66E-08	4.96E-07
HLF	6.43527	1.145688	-2.48979	2.78E-05	0.000118
AC093732	0.145802	0.543934	1.899423	3.93E-06	2.29E-05
TSPEAR-A	0.187183	0.783674	2.065802	6.25E-05	0.000233
HBB	118.5418	23.74398	-2.31976	1.85E-08	2.85E-07
MSX2	2.184333	8.028121	1.877869	1.28E-05	6.16E-05
HAVCR1	0.049423	1.212864	4.617095	1.85E-05	8.36E-05
RNU6-112	0.156135	0.496142	1.66796	0.001055	0.002641
TEX19	0.007801	0.266461	5.09418	5.74E-06	3.16E-05
SUMO1P1	0.022396	0.380267	4.08569	0.000594	0.001603

PITPNM3	5.759929	1.926481	-1.58008	0.000116	0.000395
AL049836.	0.06499	0.427406	2.717314	2.36E-05	0.000103
AC012170	0.110612	0.314263	1.506461	2.33E-05	0.000102
PKD2L1	0.046844	0.219751	2.22994	1.15E-08	1.97E-07
AL590729.	0.063435	0.287996	2.182705	6.70E-09	1.30E-07
AC036108	2.979894	0.704696	-2.08019	0.026045	0.042213
LRRC36	0.084534	0.29629	1.809399	3.76E-05	0.000153
MXRA5Y	0.146665	0.440031	1.585081	7.01E-05	0.000257
JAM2	8.187914	2.779416	-1.55871	4.10E-06	2.37E-05
AL009178.	0.131053	0.382651	1.545874	4.70E-06	2.66E-05
NUSAP1	6.597064	19.30923	1.549394	4.83E-09	9.98E-08
CD3EAP	0.633346	1.859304	1.553696	6.98E-12	2.00E-09
FANCA	0.914139	3.015151	1.721745	7.46E-10	2.43E-08
AC245884	0.065333	0.649982	3.314513	9.55E-06	4.82E-05
AC018695	0.177424	0.507897	1.517337	5.63E-08	7.02E-07
MIR5581	0.358981	1.136925	1.663159	0.000129	0.000433
AL353807.	0.095169	0.288468	1.599842	5.90E-05	0.000223
PSCA	982.5228	111.6379	-3.13766	0.000106	0.000366
DMD	5.325716	1.789781	-1.57319	0.00339	0.00724
NCLP1	0.136131	0.44845	1.719949	9.98E-09	1.78E-07
AC083822	0.113121	0.324866	1.521978	0.000786	0.002047
GHR	2.547057	0.746877	-1.76989	7.35E-08	8.68E-07
THY1	7.293307	22.49539	1.624984	4.36E-08	5.73E-07
AC114488	0.493045	1.94762	1.981922	2.02E-07	1.98E-06
ERMN	0.079834	0.240422	1.590498	2.32E-06	1.47E-05
PSORS1C3	0.578916	2.812935	2.280651	3.67E-05	0.00015
HOXC13	0.067277	1.44344	4.423261	3.26E-06	1.94E-05
MIR181B2	0.119257	0.379048	1.668308	0.002073	0.004729
LGI4	3.117143	0.817021	-1.93178	2.40E-07	2.25E-06
OPA1-AS1	0.068399	0.256895	1.909129	3.24E-07	2.89E-06
AC016550	0.026129	0.312574	3.580478	0.005791	0.011512
KRT17	7.009285	52.07617	2.893284	1.18E-05	5.73E-05
KCNA1	1.180466	0.163806	-2.8493	0.006581	0.012848
TBC1D31	0.513138	1.504634	1.551993	8.38E-12	2.04E-09
MIR544B	0.038257	0.296149	2.952515	8.14E-05	0.000292
MYOSLID	0.08237	0.591213	2.84349	6.57E-06	3.52E-05
AC079949	0.346315	1.657545	2.258893	0.01739	0.029752
C19orf84	0.048697	0.529705	3.443277	2.63E-07	2.43E-06
IGLJCOR18	3.497348	0.182339	-4.26157	0.012955	0.023044
AC053503	5.456635	1.286623	-2.08442	2.06E-05	9.15E-05
SNORD72	0.202821	0.878126	2.114223	1.92E-06	1.25E-05
FAM81A	0.443869	1.800605	2.020275	5.12E-09	1.05E-07
AC134312	0.050172	0.300932	2.584493	2.15E-08	3.23E-07
AC067931	0.189112	0.666501	1.817365	1.18E-08	2.01E-07
DUSP5P1	0.034926	0.213785	2.613799	5.20E-06	2.90E-05
AL031668.	0.073726	0.286321	1.957382	3.29E-07	2.92E-06
G2E3-AS1	0.011763	0.219096	4.219292	8.63E-06	4.42E-05
PILRB	0.456573	1.372738	1.588139	2.31E-07	2.18E-06
NUF2	1.153039	5.010525	2.119521	7.42E-11	5.16E-09
RNU1-12(	0.084377	0.29156	1.788868	0.006067	0.011989
KIAA1024	0.140489	0.430716	1.616282	1.54E-08	2.46E-07
APOBEC3I	1.640016	5.760311	1.812437	1.99E-06	1.29E-05
AC116348	0.094553	0.297012	1.651325	0.007255	0.013982
SLCO4A1-	0.595842	4.537816	2.928997	3.57E-05	0.000146
HAND2	22.3045	4.779351	-2.22245	0.004227	0.008765
SHOX2	0.069344	0.291158	2.06996	3.46E-05	0.000142
AC025857	1.736745	5.925367	1.770518	7.80E-10	2.50E-08
KCNK3	3.421023	1.173751	-1.5433	6.24E-06	3.38E-05

AC090589	0.0855	0.261084	1.610524	0.000135	0.000449
RNU4-21I	0.243361	0.847649	1.800366	0.000344	0.001001
C16orf89	22.8135	2.408377	-3.24375	2.18E-08	3.27E-07
LINC0215	0.013023	0.292768	4.490673	2.59E-07	2.40E-06
MIR302D	0.051739	0.291122	2.492314	0.000656	0.001748
AL355388.	0.186489	0.544215	1.545082	0.019108	0.032283
TMC7	0.87245	3.274388	1.908082	1.82E-09	4.68E-08
EIF5AP3	0.106799	0.314437	1.557872	6.47E-07	5.08E-06
CXCL17	225.645	66.30906	-1.76678	0.000638	0.001706
PYY2	0.070407	0.473223	2.748737	4.99E-08	6.37E-07
IGLV3-13	3.724188	0.290279	-3.68141	0.010971	0.01997
AC108693	0.18461	0.545996	1.564409	1.32E-05	6.30E-05
AC093616	0.133601	0.447453	1.74381	1.17E-09	3.35E-08
SMIM35	0.069281	0.364325	2.394685	3.41E-07	3.00E-06
AC019069	0.268354	0.8989	1.744025	9.44E-09	1.70E-07
AL353583.	0.146665	0.420128	1.518305	4.49E-06	2.56E-05
RN7SL8P	0.421873	1.235337	1.550022	0.02986	0.047538
AC008147	0.727511	2.289479	1.653979	2.43E-07	2.27E-06
DLX4	0.080281	0.697388	3.118826	3.43E-07	3.01E-06
FAM72B	0.189296	0.737041	1.961102	1.80E-09	4.63E-08
RPSAP71	0.061131	0.246845	2.013636	0.022356	0.037024
BLM	0.630161	2.455736	1.962363	5.75E-11	4.42E-09
C5orf58	0.064972	0.233915	1.848099	8.39E-05	0.000299
LINC0161	0.261364	1.489229	2.510431	7.55E-09	1.42E-07
IGF2BP3	0.256409	1.889153	2.88122	5.20E-08	6.57E-07
AC007215	0.101606	0.287555	1.500847	8.32E-05	0.000297
RNY1P4	0.102572	0.323224	1.6559	0.001944	0.004478
HELLS	0.766587	3.536512	2.205805	2.71E-12	1.40E-09
AC106772	0.061454	0.33753	2.457437	1.80E-05	8.15E-05
AC093582	0.310093	0.965139	1.638038	8.69E-06	4.44E-05
AL031666.	0.093865	0.352683	1.90972	2.12E-08	3.19E-07
KIF26B-AS	0.03908	0.579396	3.890058	4.65E-11	3.96E-09
AC073210	0.329749	1.105087	1.744719	7.98E-07	6.05E-06
AC024560	0.267019	0.885793	1.730028	1.58E-08	2.52E-07
RNU6-62I	0.080174	0.234627	1.549165	0.024744	0.040433
CENPA	1.492496	5.131475	1.781646	2.38E-09	5.74E-08
CENPO	1.038938	2.998862	1.529306	2.49E-11	2.90E-09
IL11	0.266487	2.800497	3.393548	5.14E-11	4.19E-09
MAL	382.5217	10.59947	-5.17348	5.80E-12	1.85E-09
AC018629	1.289448	3.897637	1.595846	0.012002	0.021551
GBP5	1.772565	5.400226	1.607181	3.19E-05	0.000132
DRGX	0.026983	0.40782	3.91782	0.000338	0.000987
PRIMA1	14.13141	2.377932	-2.57113	5.34E-08	6.70E-07
GABRR1	0.016079	0.254769	3.985964	4.11E-05	0.000165
PRSS51	0.08712	1.041917	3.58009	6.09E-07	4.81E-06
FAR2P1	0.008552	0.523212	5.935013	1.81E-07	1.80E-06
RNU4-78I	0.295402	0.881603	1.577451	6.40E-05	0.000238
AC245100	0.038337	1.59392	5.377696	0.003674	0.007766
PROZ	0.053052	0.237567	2.162853	2.13E-05	9.42E-05
LINC0127	0.083645	0.344076	2.040374	1.15E-06	8.21E-06
RPS3AP3	0.040201	0.255922	2.670392	1.70E-06	1.13E-05
AL133215.	0.204057	0.636324	1.640788	3.48E-05	0.000143
TIMP1	125.996	413.2126	1.713507	7.92E-10	2.53E-08
AL513123.	0.016201	0.434604	4.745507	2.26E-07	2.15E-06
MAPK4	2.014051	0.500253	-2.00937	1.24E-05	6.00E-05
AL133330.	0.286193	0.921367	1.686789	0.000221	0.000684
AC244093	0.192308	0.623115	1.696082	4.17E-07	3.53E-06
HOXC6	0.374199	1.841171	2.298746	4.93E-08	6.29E-07

AGTR1	2.460755	0.752889	-1.70859	1.58E-05	7.31E-05
SCUBE2	2.391397	0.812226	-1.5579	1.50E-06	1.02E-05
ANK1	0.327073	1.278665	1.966956	0.025886	0.041997
MTCO3P4	0.047607	0.314876	2.725542	2.55E-09	6.06E-08
C10orf91	0.139461	0.568369	2.02697	2.20E-05	9.67E-05
SSC4D	0.173319	0.668981	1.948535	1.82E-08	2.81E-07
AC016292	0.115043	0.340573	1.56579	0.001045	0.002619
TNFRSF11	0.506766	5.18658	3.355391	1.06E-07	1.18E-06
KRT7	3.152407	42.1481	3.740942	1.19E-05	5.78E-05
DUXAP9	0.016551	0.222056	3.745917	2.41E-10	1.06E-08
ADAM12	0.350509	2.456506	2.809082	1.79E-11	2.51E-09
CSMD2	0.068511	0.25077	1.871954	3.04E-06	1.84E-05
PLOD3	9.598509	27.16921	1.501091	2.60E-10	1.12E-08
SEMA6D	3.088962	1.056825	-1.54739	0.002859	0.006241
AC090912	0.086598	0.256698	1.567667	8.77E-05	0.000311
FHL1	112.7931	22.61589	-2.31827	3.56E-08	4.87E-07
AL033527.	0.047063	0.21826	2.213383	8.76E-08	1.00E-06
ADCY5	9.518937	3.18649	-1.57883	0.001097	0.002731
BRIP1	0.418488	1.540339	1.87999	1.68E-10	8.35E-09
TMOD1	7.903366	2.060599	-1.9394	1.85E-06	1.22E-05
SNRPCP4	0.085716	0.272971	1.671116	0.00012	0.000406
GK-IT1	0.317487	0.988498	1.63854	9.41E-07	6.95E-06
RN7SKP29	0.098986	0.497458	2.329284	1.17E-07	1.27E-06
KIF14	0.381309	2.333505	2.613465	3.81E-12	1.46E-09
GAD1	0.04323	0.780508	4.174301	7.92E-10	2.53E-08
CCNE1	1.38516	11.37574	3.037836	5.50E-09	1.11E-07
RNF32	0.307553	0.878164	1.513653	8.17E-08	9.47E-07
AL627230.	0.079059	0.362872	2.19847	3.12E-06	1.88E-05
AP002954	0.192987	0.546988	1.503004	0.001055	0.002641
AL035420.	0.069521	0.221455	1.671496	0.000803	0.002086
PIMREG	0.858209	2.899594	1.75645	5.50E-09	1.11E-07
COMP	0.859207	10.91113	3.66665	1.68E-06	1.12E-05
OTX1	0.359169	1.847771	2.363053	2.60E-10	1.12E-08
RBPMS2	41.60266	9.280238	-2.16444	5.97E-06	3.25E-05
AL133373.	0.105991	0.399157	1.913014	7.75E-05	0.000279
PCLAF	2.861725	8.871945	1.632365	6.91E-09	1.32E-07
AC010761	0.325478	0.934043	1.520928	2.93E-06	1.78E-05
TMSB15A	0.173222	0.632444	1.868316	3.43E-07	3.01E-06
ARID3A	1.809928	6.085069	1.749341	0.000102	0.000354
GAPDHP3	0.06431	0.212654	1.725393	2.05E-07	2.00E-06
RSPO2	2.374383	0.530708	-2.16156	1.96E-07	1.92E-06
CCDC78	0.157151	0.489283	1.638517	1.88E-06	1.23E-05
SERPINE1	8.532432	37.97013	2.153836	1.63E-07	1.66E-06
SLC28A3	0.585203	2.476084	2.081052	2.99E-05	0.000125
AC008521	0.171745	0.646942	1.913367	4.06E-05	0.000163
AC092953	0.102178	0.30649	1.584762	0.000302	0.000896
RNU7-12P	0.103797	0.416984	2.006229	0.001596	0.003772
UBE2QL1	1.464955	0.437195	-1.74451	3.75E-07	3.23E-06
MTFR2	0.736117	2.973051	2.013937	1.58E-10	8.08E-09
HENMT1	1.453286	4.825091	1.731238	1.65E-08	2.61E-07
LINC0159	0.038654	0.28742	2.89446	0.000446	0.001252
CDK1	3.64179	13.21298	1.859237	6.81E-10	2.26E-08
RNU6-12(	0.089424	0.274157	1.616273	0.009305	0.017345
IGKV1-37	1.19092	0.138424	-3.10491	0.01293	0.023003
MET	4.709488	24.7771	2.395365	5.94E-11	4.49E-09
AP001107	2.709791	0.397431	-2.7694	2.80E-07	2.55E-06
LONRF2	1.737046	0.546237	-1.66904	0.000428	0.001209
GSTM1	13.73754	4.584922	-1.58315	0.010048	0.018521

CKS1BP3	0.145322	0.508048	1.805714	3.39E-07	2.99E-06
AC093702	0.909446	0.233446	-1.9619	0.006228	0.012258
BUB1	1.745675	7.101148	2.024267	2.00E-10	9.43E-09
WT1	0.036504	0.279816	2.938374	5.44E-05	0.000208
SST	112.1308	35.07082	-1.67684	5.15E-05	0.000199
FAM19A5	0.809813	2.514793	1.63478	3.09E-05	0.000129
AC006449	0.077985	0.286377	1.876658	2.28E-07	2.16E-06
DNA2	0.586243	2.51547	2.101258	1.23E-11	2.34E-09
AC099489	0.071309	0.224771	1.656293	0.000173	0.000553
KLF15	5.457345	1.612599	-1.75881	2.31E-07	2.18E-06
GRIN3B	0.087565	0.36883	2.074529	0.001582	0.003744
CNTD2	0.198598	1.293212	2.703033	3.16E-06	1.90E-05
RF00405	0.236235	0.70243	1.57213	0.000606	0.00163
CHI3L1	2.329014	13.86624	2.573786	6.40E-07	5.02E-06
RBL1	1.339955	3.995137	1.576061	7.34E-12	2.00E-09
FABP6	0.846414	2.635516	1.63865	0.000402	0.001144
AL365356.	0.074923	0.388386	2.374001	0.002019	0.004623
ADCYAP1I	1.084637	0.149072	-2.86313	4.63E-07	3.85E-06
CLDN14	0.078611	0.256646	1.706967	6.53E-06	3.50E-05
ASB5	3.836821	0.721695	-2.41045	0.000316	0.000934
AC084083	0.145319	0.444353	1.612484	1.04E-05	5.18E-05
PODXL	6.21197	17.57252	1.500199	3.82E-10	1.47E-08
CIP2A	0.877135	3.217728	1.875172	7.34E-12	2.00E-09
DUSP5	30.21641	9.601852	-1.65395	0.000535	0.001466
PBK	3.333874	11.48103	1.783981	2.18E-08	3.27E-07
RCC2	16.38553	47.97339	1.549812	6.53E-12	1.99E-09
AC060773	0.064927	0.252433	1.959004	3.38E-05	0.000139
OXTR	0.179712	0.763262	2.08649	2.96E-09	6.79E-08
Z93242.1	0.100694	0.377152	1.905173	1.60E-05	7.37E-05
GHRL	16.46639	2.613006	-2.65574	0.017166	0.029411
AL121601.	0.092314	0.35214	1.931526	1.79E-07	1.79E-06
ANGPT2	0.656811	2.71281	2.046239	2.11E-11	2.71E-09
ENDOU	10.35717	0.376616	-4.78139	2.75E-05	0.000117
FAM129A	33.94799	11.05429	-1.61872	0.000199	0.000627
MYMX	1.446559	0.482627	-1.58364	8.39E-05	0.000299
LINC0151	0.021574	0.468444	4.440485	0.001079	0.002692
RNU6-18	0.147291	0.483927	1.716123	0.001682	0.003944
BHMT2	2.814713	0.844032	-1.73762	1.05E-05	5.23E-05
STK31	0.09754	1.065588	3.449519	2.45E-09	5.85E-08
AL691482.	0.660029	2.776972	2.072912	1.23E-07	1.32E-06
CHST13	0.417156	3.711403	3.153304	0.000122	0.000412
AC012531	0.005964	0.276898	5.53685	3.62E-05	0.000148
AL807752.	0.082557	0.235652	1.51319	9.51E-08	1.07E-06
AC091153	0.189026	0.579103	1.615232	2.91E-05	0.000123
SMIM25	0.667162	2.298284	1.784447	2.79E-08	3.97E-07
AC131571	0.09353	0.322809	1.787186	2.66E-05	0.000114
CXCL11	2.732417	10.67899	1.966526	4.29E-06	2.47E-05
MYO16-A	0.043663	0.279101	2.676289	0.012303	0.022012
LINC0046	0.053928	0.518499	3.265236	1.06E-07	1.18E-06
HPX	0.054604	0.323528	2.566807	6.03E-05	0.000226
RNA5SP2	0.139336	0.45699	1.7136	0.000996	0.002515
TDGF1P5	0.081017	0.271348	1.743837	2.19E-06	1.40E-05
LINC0236	0.03008	0.615169	4.354125	4.15E-06	2.39E-05
ASB2	9.957121	3.096603	-1.68504	9.00E-05	0.000318
CASQ2	16.75823	2.769882	-2.59697	9.20E-08	1.04E-06
ACTG1P3	0.21612	0.665889	1.62345	1.01E-09	2.99E-08
MKRN9P	0.066243	0.267246	2.012333	0.020805	0.034797
FNDC5	2.384122	0.706023	-1.75567	4.49E-06	2.56E-05

CHML	1.105429	3.405939	1.623447	3.49E-11	3.34E-09
AC023825	0.101847	0.332357	1.706329	1.66E-06	1.11E-05
AC073283	0.100963	0.527319	2.384856	1.61E-08	2.55E-07
AC110588	0.083445	0.299462	1.843474	4.70E-05	0.000184
LINC0050:	0.058824	0.270259	2.199862	4.51E-06	2.57E-05
FANCI	1.653563	6.556279	1.987299	2.61E-11	2.91E-09
AL157871.	0.314608	0.988636	1.651885	0.000247	0.000755
PLA2G4E	0.076835	0.29413	1.936613	0.000496	0.001372
MYOT	1.334454	0.255202	-2.38654	3.61E-08	4.92E-07
IGHV3OR:	2.717339	0.433935	-2.64664	0.014387	0.025257
IGHV3-16	1.889452	0.175984	-3.42445	0.026853	0.043373
SULT2A1	6.500524	2.215415	-1.55298	0.00324	0.006955
AGBL2	0.127408	0.375496	1.559347	5.38E-07	4.36E-06
PHF5CP	0.103523	0.329763	1.671481	3.94E-06	2.29E-05
AC139720	0.035375	0.254385	2.846208	0.0068	0.013224
AC007406	0.109967	0.488774	2.152097	5.99E-10	2.04E-08
LINC0167:	0.110592	0.644803	2.543606	0.02268	0.037495
AC243967	0.039009	0.326708	3.066128	4.42E-05	0.000175
SNORA58	0.119723	0.373544	1.641579	0.000981	0.002483
ANLN	2.79978	12.08604	2.109956	2.61E-11	2.91E-09
NRIR	0.13001	0.44152	1.76386	1.46E-05	6.86E-05
UBE2T	7.319158	21.88746	1.580355	6.81E-09	1.31E-07
AL589743.	0.054381	0.342715	2.655839	3.44E-08	4.74E-07
RNA5SP3ᛘ	0.015847	0.603809	5.251847	0.000166	0.000535
AL136418.	0.193911	1.094608	2.496946	0.000132	0.00044
AL354726.	0.053112	0.221251	2.058583	1.26E-06	8.87E-06
LINC0174ᛘ	0.071023	0.605946	3.092838	3.70E-05	0.000151
CORIN	0.072215	0.394485	2.449594	1.53E-09	4.10E-08
SNORD62	0.120552	0.690776	2.518565	6.05E-07	4.80E-06
CLSPN	0.592713	3.211715	2.437938	2.00E-12	1.40E-09
CYP1A1	1.409894	0.171171	-3.04208	0.000348	0.001011
TAGLN	378.3015	123.5203	-1.61479	8.23E-05	0.000294
SGCG	0.797792	0.162325	-2.29712	1.43E-07	1.49E-06
DEFA5	624.081	16.90695	-5.20604	0.000805	0.00209
NKAIN4	0.139897	0.492958	1.817103	0.002152	0.004887
ATAD2	2.200656	9.937563	2.174958	9.51E-13	1.31E-09
FERP1	0.111967	0.325774	1.540802	3.37E-06	2.00E-05
MAGEA6	0.015262	5.290794	8.437411	0.000246	0.000752
CYP3A4	25.12219	1.751315	-3.84245	0.016295	0.028133
AL139002.	0.002147	0.274763	6.999389	0.000342	0.000998
AC024560	0.218631	0.820429	1.907879	6.35E-08	7.74E-07
AL035252.	0.166531	0.4802	1.527848	2.76E-06	1.70E-05
RPS15AP6	0.196985	0.579744	1.557328	1.83E-05	8.28E-05
KRT1	8.199241	0.200257	-5.35556	0.001736	0.004055
ERVMER3⁴	0.356688	1.627895	2.190271	0.003187	0.006857
PDZRN4	6.704642	0.846963	-2.98479	3.14E-06	1.89E-05
ORC1	1.123024	3.769197	1.746868	2.96E-09	6.79E-08
LNCAROD	0.015736	0.296681	4.236751	0.023688	0.038946
MIR647	4.459194	13.11955	1.556864	4.96E-07	4.09E-06
MYOCD	7.429524	2.474448	-1.58616	0.000114	0.000389
MCM10	0.488646	2.494674	2.351988	5.66E-11	4.42E-09
RAD54B	0.237866	0.828216	1.79986	6.86E-11	4.89E-09
ACTL8	0.02676	3.40599	6.991849	8.82E-06	4.50E-05
HAND2-A	5.837247	1.031479	-2.50057	0.001164	0.002876
MTATP6P:	0.091413	0.265325	1.537295	0.000794	0.002063
RNU6-13:	0.098135	0.397013	2.016346	0.000138	0.000457
AC005046	0.483313	1.372809	1.506101	6.76E-06	3.60E-05
HSPB6	466.8402	98.74979	-2.24108	4.81E-06	2.71E-05

AL365440.	0.071358	0.210907	1.563452	1.05E-07	1.17E-06
MRGPRF	30.66792	10.26357	-1.5792	0.005858	0.011617
CEACAM6	57.47068	226.4174	1.978087	0.002367	0.005301
PLEKHG4	0.707764	3.975509	2.489799	5.35E-10	1.87E-08
AL031665.	0.039539	0.263099	2.734264	0.000345	0.001005
AP002336	0.062144	0.221365	1.83275	2.03E-05	9.03E-05
PMFBP1	0.102321	0.440841	2.107154	8.48E-07	6.37E-06
VIT	1.713962	0.301627	-2.5065	3.04E-09	6.94E-08
AC090246	0.070292	0.244074	1.795886	1.92E-05	8.61E-05
CYP4A22-	0.084404	0.392643	2.217834	1.23E-08	2.08E-07
NRCAM	0.396781	2.064509	2.379383	0.001745	0.004071
RNY1P13	0.150795	0.535024	1.827015	5.60E-05	0.000213
FAM111B	1.594442	4.739363	1.571642	7.65E-08	8.97E-07
AP000786	0.229991	0.800635	1.799566	4.51E-05	0.000178
DAB1	1.395323	0.269894	-2.37013	3.92E-06	2.28E-05
INSYN1	1.418749	0.434815	-1.70615	5.04E-06	2.82E-05
HOXC13-/	0.006251	0.248154	5.311093	0.000166	0.000535
RNU6-45(	0.162428	0.477258	1.554968	0.00085	0.002191
AL591767.	0.041371	0.230873	2.480414	6.54E-09	1.27E-07
CDC45	1.623216	5.327151	1.714509	2.24E-09	5.47E-08
AC138466	0.087067	0.264139	1.601098	1.38E-06	9.54E-06
OBP2B	0.011396	0.337705	4.889168	0.000156	0.00051
DEFA6	207.7274	11.42037	-4.18501	0.017143	0.029393
CD44-AS1	0.396391	1.19564	1.592786	3.69E-05	0.00015
AC245100	0.022726	0.340063	3.903394	0.002889	0.006298
TYRP1	0.418783	1.215333	1.537078	0.013455	0.023824
MTND3P1	0.025209	0.225182	3.15907	2.65E-08	3.82E-07
CXCR2	3.904955	1.00625	-1.95632	0.00517	0.010437
SAPCD2	4.177994	15.0205	1.84605	4.25E-09	8.97E-08
AC106900	0.166519	1.047724	2.653504	3.78E-07	3.27E-06
AC009237	0.408745	1.171374	1.518929	7.90E-05	0.000284
RNU4ATA	0.180062	0.86528	2.264676	5.03E-07	4.13E-06
FAXDC2	7.405937	2.561916	-1.53146	3.11E-06	1.87E-05
C1QTNF7	3.095701	0.865507	-1.83865	5.73E-05	0.000217
TNFSF11	0.162143	1.099128	2.761017	1.00E-10	6.06E-09
RNU7-75F	0.190303	0.948079	2.316708	2.12E-06	1.36E-05
PIWIL1	0.012974	1.109053	6.417531	5.70E-08	7.08E-07
MTBP	0.267892	1.1015	2.039744	2.37E-12	1.40E-09
AC062037	0.083575	0.23973	1.520261	0.001518	0.003616
LGALS9C	5.594538	0.879545	-2.66919	0.00065	0.001732
AC090912	0.056759	0.344491	2.601549	1.32E-07	1.41E-06
AC012512	0.046515	0.563586	3.59886	1.54E-05	7.16E-05
RNY3P13	0.078545	0.349992	2.155737	0.00108	0.002693
GPT	9.360461	3.202362	-1.54744	0.027354	0.044087
NSG2	0.835872	0.277748	-1.58951	4.24E-06	2.44E-05
VCAN	4.4256	15.06808	1.767551	3.07E-08	4.31E-07
WISP3	0.234143	1.103391	2.236481	0.00366	0.007738
LINGO1	0.567298	2.262762	1.995906	8.08E-07	6.12E-06
SNORA22	0.19386	0.589641	1.604821	5.91E-06	3.22E-05
AC009097	0.118117	0.378967	1.68186	8.86E-06	4.51E-05
ILDR1	1.150775	4.196454	1.866565	3.89E-09	8.45E-08
SLCO1B3	0.124859	0.748527	2.583754	4.12E-05	0.000165
SYNM	120.6229	26.57575	-2.18232	7.99E-05	0.000287
PSMD10P.	0.272354	1.023236	1.909584	1.89E-09	4.82E-08
ITGA8	10.79317	3.006739	-1.84385	5.85E-05	0.000221
LINC0247.	0.124485	1.454177	3.546158	3.21E-05	0.000133
AC079907	0.132358	0.396734	1.583724	1.37E-06	9.50E-06
AC093458	0.096158	0.341234	1.827287	3.96E-05	0.00016

BMP8A	0.60697	2.600769	2.09924	1.74E-10	8.46E-09
EFS	6.476746	2.011959	-1.68667	2.04E-05	9.10E-05
MIR6797	0.17833	0.700142	1.973098	3.09E-06	1.86E-05
LINC0108‸	4.862619	1.479632	-1.71649	1.37E-07	1.44E-06
RPSAP74	0.06431	0.223552	1.797502	0.000116	0.000396
METTL27	1.086041	4.196715	1.950182	1.58E-05	7.31E-05
CLEC5A	0.202242	0.882201	2.125022	2.05E-09	5.11E-08
AC100797	0.069609	0.255485	1.875894	0.000186	0.000593
AC021876	0.021513	0.298528	3.794572	1.07E-06	7.78E-06
MMP12	10.51363	32.83861	1.643133	0.000215	0.000668
AARD	1.38844	0.379856	-1.86994	0.000357	0.001033
RNU1-124	0.063766	0.261162	2.034089	0.000288	0.000861
RNU6-122	0.168949	0.479207	1.504056	0.00052	0.00143
KCNB1	1.809859	0.346594	-2.38456	5.51E-06	3.05E-05
E2F3	1.658168	5.125412	1.628078	2.37E-13	1.20E-09
CYP4F22	4.360956	0.440955	-3.30594	0.000728	0.001912
AC016866	0.074846	0.345716	2.207598	2.15E-06	1.38E-05
TICRR	0.665639	2.486593	1.901358	3.09E-09	7.01E-08
IGF2BP1	0.02238	1.789484	6.321217	7.77E-06	4.05E-05
AL031281.	0.13878	0.426724	1.620501	6.95E-06	3.68E-05
LSAMP-AS	0.097014	0.388357	2.001115	0.002458	0.00548
PTGDR2	3.104808	0.727399	-2.09369	0.010118	0.018627
MORN5	5.478905	0.976468	-2.48824	0.003342	0.007145
PTCHD1	1.629107	0.274667	-2.56832	4.22E-05	0.000168
C6orf58	41.72364	14.51317	-1.5235	0.025726	0.041812
AL591178.	0.108407	0.495385	2.192093	0.000259	0.000786
NUP62CL	0.476405	2.019506	2.083743	1.31E-07	1.39E-06
ANKRD1	0.028841	0.717359	4.636483	4.59E-06	2.61E-05
DNAJC19P	0.053934	0.420307	2.962175	9.15E-07	6.79E-06
SLC11A1	0.488257	1.584628	1.698431	7.55E-08	8.86E-07
QRSL1	3.108025	10.55351	1.763653	9.55E-06	4.81E-05
AC112777	0.201986	0.811919	2.007082	8.67E-10	2.70E-08
AL137802.	0.082525	0.313432	1.925253	0.000119	0.000405
AC016542	0.072645	0.320688	2.142241	2.76E-07	2.52E-06
ATP1B2	4.004607	1.292072	-1.63197	0.000638	0.001706
HAGLROS	0.239059	0.762716	1.673778	0.000351	0.001018
AC073592	0.038002	0.272831	2.843868	1.54E-05	7.17E-05
CDC6	1.578902	10.38101	2.716953	1.85E-10	8.91E-09
AL354993.	0.082315	0.259046	1.653976	0.002293	0.005156
DSCC1	1.24427	5.224318	2.069944	3.33E-12	1.40E-09
AL357033.	0.037416	0.265924	2.829283	0.000744	0.001951
ENPP3	1.881674	0.266783	-2.81828	8.31E-05	0.000297
MIR16-1	0.086809	0.347614	2.001574	0.000899	0.0023
LINC0065‸	0.740642	2.550265	1.783798	2.16E-05	9.51E-05
AC090559	0.288322	0.819006	1.506194	0.000103	0.000357
ANK2	2.996087	1.037073	-1.53056	0.001008	0.002537
AC007996	0.495885	1.490766	1.587977	9.33E-08	1.05E-06
MIR3680-	0.10746	0.351938	1.711522	0.000885	0.002269
HJURP	1.407605	5.616995	1.996556	2.77E-10	1.18E-08
RERG	10.45102	2.658815	-1.97479	0.009842	0.018198
NRBF2P5	0.230294	0.652374	1.502222	2.39E-07	2.24E-06
LINC0170‸	0.126739	1.035889	3.030931	1.07E-07	1.19E-06
RNA5SP21	0.410141	1.262173	1.621718	5.70E-06	3.14E-05
PLCD4	2.644367	0.817221	-1.69412	0.028545	0.045738
AL391095.	0.062936	0.288166	2.194949	1.47E-07	1.53E-06
MIR643	0.143021	0.512314	1.8408	4.61E-05	0.000181
TMEM26	0.087853	0.376028	2.097672	9.11E-12	2.07E-09
RNU6-875	0.056911	0.446536	2.971992	3.04E-06	1.84E-05

SNORD12	0.498473	1.595095	1.678057	1.25E-06	8.77E-06
E2F1	2.880725	11.07106	1.94229	5.84E-11	4.46E-09
RNU6-702	0.037029	0.377585	3.350068	7.43E-06	3.90E-05
AP000523	0.09678	0.43384	2.164385	1.42E-07	1.48E-06
ARHGEF38	0.091587	0.476728	2.379946	3.34E-07	2.96E-06
RNU7-18P	0.216856	0.778475	1.843913	0.000655	0.001746
S100A8	795.2633	69.01211	-3.52651	0.002424	0.005409
CFAP157	0.249759	0.78712	1.656045	8.53E-12	2.04E-09
AZU1	0.033719	0.63675	4.239106	0.005281	0.010642
AL121772.	0.125179	0.587098	2.229611	4.98E-06	2.79E-05
HSPA2	11.78369	3.067673	-1.94158	0.00044	0.001238
AL356320.	0.149383	0.746601	2.321318	1.15E-07	1.25E-06
AC068658	0.029541	0.37225	3.6555	0.003845	0.008079
MIR6069	0.081126	0.229493	1.500224	0.007388	0.014204
AF001548.	5.139606	1.39528	-1.8811	0.000562	0.001529
CHIT1	0.107316	1.361995	3.665783	0.000137	0.000455
FIRRE	0.042223	0.407782	3.271682	1.89E-09	4.82E-08
FXYD6	15.86424	3.631172	-2.12727	0.000526	0.001443
AL133373.	0.063187	0.49649	2.974061	0.000156	0.000508
SHISA2	0.917535	3.201186	1.802771	0.002134	0.004851
FOXD2-AS	0.682004	2.923125	2.099659	5.40E-11	4.30E-09
FBXO41	0.835717	2.585791	1.629519	2.12E-08	3.19E-07
LINC0134	0.130405	0.517776	1.989334	0.000703	0.001855
AC112206	0.041042	0.255287	2.636945	0.009797	0.018129
SPINK2	2.288242	0.244273	-3.22767	0.000146	0.000483
LINC0225	0.025857	0.27809	3.426937	0.007893	0.015045
AC023825	0.040793	0.242563	2.571958	3.04E-09	6.94E-08
AL121895.	0.091857	0.340939	1.892051	8.91E-07	6.63E-06
AP005899	0.319355	0.984386	1.624061	1.08E-07	1.19E-06
KCNJ16	2.03399	0.360686	-2.4955	0.003363	0.007189
AC087072	0.072083	0.216621	1.587442	7.11E-05	0.00026
CIDEC	22.14855	2.16126	-3.35727	0.00037	0.001067
IL37	0.018372	0.380323	4.371682	1.51E-08	2.44E-07
AC103760	0.256169	0.803921	1.649958	2.70E-06	1.67E-05
WDR66	0.063855	0.255819	2.002241	5.71E-08	7.08E-07
MMS22L	0.302713	0.945913	1.643759	2.41E-11	2.88E-09
AC005840	0.237632	0.704117	1.567087	4.69E-07	3.89E-06
KCNJ14	0.195071	0.666582	1.77278	1.12E-10	6.54E-09
AF127577.	0.008118	0.389628	5.584754	5.26E-08	6.64E-07
BRCA2	0.484314	1.855185	1.93755	6.75E-11	4.85E-09
Z98200.1	0.097984	0.305124	1.638777	1.05E-05	5.22E-05
GINS1	1.360942	5.675978	2.060263	6.86E-11	4.89E-09
BTBD16	0.109234	1.024589	3.22955	5.34E-09	1.08E-07
MIR4269	1.582641	0.487684	-1.69832	0.022108	0.036671
SYNDIG1	0.628581	1.816011	1.530602	6.09E-05	0.000228
EMILIN3	0.983754	0.314817	-1.64378	3.94E-07	3.37E-06
TPX2	6.50795	32.80875	2.333806	2.92E-11	3.09E-09
SNORD1B	0.141239	0.702875	2.315135	1.41E-06	9.72E-06
SIX4	0.343517	1.142341	1.733541	6.24E-06	3.38E-05
RN7SKP17	0.066961	0.287732	2.103338	2.51E-07	2.33E-06
AL118505.	0.329745	1.272536	1.948285	4.60E-08	5.99E-07
HNF1A-AS	1.648452	7.024875	2.091361	8.85E-08	1.01E-06
RNU6-610	0.126603	0.668552	2.400727	2.45E-05	0.000106
RNU6-282	0.268096	0.787503	1.554537	2.53E-05	0.000109
AC055748	0.074588	0.318665	2.095029	6.77E-05	0.00025
LINC0049	0.115724	0.440218	1.927524	0.001634	0.003849
MIR3174	0.262202	0.960648	1.873328	6.09E-05	0.000228
AC005041	0.274633	0.918464	1.741717	2.24E-08	3.34E-07

Z82243.1	0.38048	1.07999	1.505124	4.48E-08	5.85E-07
IGHV3OR1	3.615554	0.527648	-2.77657	0.015914	0.027571
AC106820	0.239925	0.681618	1.506379	7.88E-07	5.99E-06
RPL23AP2	0.071427	0.23353	1.709074	7.43E-05	0.00027
CDC25A	1.018821	2.99663	1.55644	3.42E-08	4.71E-07
TONSL	2.196441	6.921604	1.655939	4.59E-10	1.68E-08
AC126696	0.059266	0.210988	1.831893	6.24E-05	0.000233
RNU6-89(	0.198579	0.603678	1.604068	0.000144	0.000475
RNU1-10(	0.331988	1.74358	2.392849	6.95E-09	1.33E-07
IGKV1D-3	2.09835	0.164766	-3.67077	0.003933	0.00824
PGGHG	2.506939	8.549963	1.769991	1.15E-07	1.26E-06
IGBP1-AS	0.090867	0.327007	1.847494	4.36E-08	5.73E-07
NPY6R	1.786743	0.292798	-2.60936	0.001238	0.003036
CACNG8	0.043017	0.235132	2.450492	2.29E-06	1.45E-05
RNU6-10(	0.235336	0.781259	1.731078	6.70E-06	3.58E-05
AL356652.	0.117419	0.456387	1.958596	7.15E-08	8.48E-07
PCSK2	1.07589	0.298396	-1.85023	1.22E-08	2.07E-07
AC104809	0.031131	0.37118	3.575681	0.019081	0.032242
GTF2IP23	0.581209	1.988892	1.774836	1.17E-08	1.99E-07
S100A7	3.811125	98.84484	4.696877	0.003483	0.007419
SNRPCP1	0.115077	0.389865	1.760373	0.000372	0.00107
LINC0254	0.799367	3.786133	2.243796	0.012839	0.022864
SNORD53	0.187617	0.617587	1.71885	0.00011	0.000377
CCDC144I	0.073332	0.502094	2.775443	4.39E-05	0.000174
LMO7-AS	0.282576	0.898686	1.669176	1.90E-05	8.53E-05
HNRNPA1	0.24834	0.953188	1.940445	1.61E-10	8.13E-09
GNG7	5.904562	1.684822	-1.80923	3.13E-10	1.27E-08
STC2	0.477216	1.685847	1.820758	6.79E-08	8.14E-07
ESRRG	2.437658	0.345677	-2.818	3.33E-08	4.61E-07
MLNR	2.258565	0.388486	-2.53947	0.014604	0.025587
RF00340	0.073356	0.215324	1.553517	0.002258	0.005091
SLC25A34	3.107536	0.645565	-2.26714	0.001433	0.003444
HOXB9	1.932982	7.068486	1.870573	4.41E-07	3.69E-06
MMRN1	4.887329	1.52169	-1.68337	1.54E-08	2.46E-07
HAL	0.109646	0.353807	1.690106	0.014572	0.025535
COL5A2	8.854249	33.12449	1.903456	2.85E-10	1.20E-08
RNU4-80I	0.678468	2.488018	1.874645	0.000414	0.001174
AL390726.	0.102465	0.38903	1.924757	0.0017	0.003983
NEB	0.048513	1.049055	4.434561	1.53E-09	4.10E-08
SH3GL2	1.104182	0.186181	-2.5682	3.96E-09	8.55E-08
RNU6-92	0.413024	1.477691	1.839046	1.25E-07	1.34E-06
MUCL1	0.025457	1.410922	5.792435	0.007337	0.014117
RNU6-62	0.071638	0.319199	2.155659	0.000148	0.000487
AC011465	0.287294	1.237217	2.106498	2.67E-09	6.29E-08
ABCA8	2.957319	1.042353	-1.50445	1.67E-06	1.11E-05
MT1X	35.88993	11.44144	-1.64931	3.06E-07	2.75E-06
SLC16A8	0.187439	0.57446	1.615789	4.87E-06	2.74E-05
CXCL6	0.309888	4.176066	3.752326	1.11E-07	1.22E-06
IBSP	0.088482	0.760205	3.102931	1.46E-09	3.96E-08
AC136475	1.761686	7.180406	2.027108	1.24E-05	6.00E-05
TRPM2-A	0.220076	1.959325	3.154283	8.43E-09	1.55E-07
CLCA1	59.75222	4.984992	-3.58333	0.010816	0.019727
RN7SL368	0.429833	2.480934	2.529036	1.64E-09	4.34E-08
GSTM5	2.706607	0.556247	-2.28269	8.04E-10	2.55E-08
SEMA5B	0.165185	0.562405	1.767529	8.43E-09	1.55E-07
CELSR3	0.448335	2.028006	2.177413	3.88E-10	1.48E-08
SP8	0.006513	0.376377	5.852727	1.73E-07	1.74E-06
AC140479	0.037589	0.263935	2.811817	1.79E-08	2.79E-07

ARHGEF25	9.750509	3.253662	-1.58341	0.000814	0.002112
LINC0183	0.030415	1.123811	5.207474	2.12E-07	2.06E-06
ATAD5	0.620342	2.168704	1.805697	7.98E-12	2.04E-09
RANBP17	0.131944	0.442864	1.746938	0.000157	0.00051
NR4A3	11.70857	2.75722	-2.08628	0.025886	0.041997
CASC8	0.23759	0.748027	1.654617	9.45E-05	0.000331
C9orf50	0.089438	0.272633	1.607997	2.18E-05	9.59E-05
ACTN2	2.747867	0.566454	-2.27828	0.012002	0.021551
AC131009	0.500533	2.029177	2.019356	3.33E-11	3.27E-09
MFAP2	1.727514	8.963222	2.37532	1.52E-08	2.44E-07
SLC4A11	1.720538	5.628918	1.709998	4.10E-06	2.37E-05
CIDEA	1.896366	0.17949	-3.40127	1.04E-09	3.06E-08
RNU6-117	0.08364	0.327177	1.967809	0.000307	0.000909
KCNMB2-	0.025381	0.91945	5.178963	2.22E-08	3.32E-07
AL358232.	0.298603	1.543134	2.369561	5.78E-08	7.15E-07
AL021407.	0.063935	0.284367	2.153084	8.68E-06	4.44E-05
ANOS1	0.516173	1.816811	1.815481	1.74E-07	1.75E-06
GPR19	0.162033	0.47899	1.563707	3.47E-07	3.04E-06
SBK1	0.588992	2.261128	1.940722	0.002405	0.005371
SNORD70	0.214097	0.843007	1.977281	1.92E-06	1.25E-05
AC008443	0.028486	0.453557	3.992983	6.87E-05	0.000253
CLDN1	9.597586	27.70942	1.529633	1.56E-08	2.49E-07
RN7SL329	0.19168	0.567654	1.566311	2.51E-05	0.000108
CCDC187	0.065274	0.31778	2.28344	0.003389	0.00724
AL442067.	0.103841	0.32295	1.636941	5.48E-05	0.000209
AC026250	0.077791	0.249501	1.681364	5.72E-07	4.58E-06
RN7SKP97	0.094863	0.373519	1.977268	6.30E-07	4.96E-06
PLK4	1.100018	3.447757	1.648131	1.44E-09	3.92E-08
ATP1A2	9.423339	1.794094	-2.39298	1.14E-07	1.24E-06
AL023284.	1.398007	4.086319	1.54743	3.29E-06	1.96E-05
SNORA71	0.462509	2.618908	2.501411	1.66E-10	8.26E-09
AC090116	0.009468	0.40235	5.409283	2.15E-06	1.38E-05
ANKRD10	2.40275	8.558995	1.832755	6.71E-10	2.24E-08
AC245884	0.272422	0.780549	1.518648	5.45E-07	4.40E-06
EIF4HP2	0.211023	0.623954	1.564042	2.40E-07	2.25E-06
CEMIP	1.005536	10.70953	3.41286	6.03E-11	4.52E-09
OR7E128F	0.112877	0.464997	2.042467	7.31E-09	1.39E-07
ECT2	4.10072	20.73156	2.33788	8.96E-12	2.06E-09
AP002478	0.007655	0.302651	5.305067	7.48E-10	2.43E-08
AC008760	0.668238	2.24332	1.747201	0.000101	0.000351
AP000344	0.029338	0.2254	2.941621	0.002573	0.005704
AC012213	0.107428	0.307584	1.517614	7.79E-07	5.93E-06
TRIM72	0.04067	0.349194	3.101992	0.00058	0.001571
MIR27B	2.203966	0.670152	-1.71754	0.002059	0.004701
TUFMP1	0.088348	0.331265	1.906714	0.005325	0.010711
RF00494	0.104966	0.333633	1.668344	3.23E-06	1.93E-05
AL591806.	0.215871	0.772612	1.839572	3.39E-06	2.01E-05
AC005479	0.076069	0.24889	1.710119	9.22E-06	4.67E-05
C1orf112	0.538796	1.720736	1.675215	2.37E-12	1.40E-09
RN7SL381	0.252021	0.844296	1.744205	1.82E-08	2.81E-07
TTK	1.216796	4.92455	2.016905	9.49E-10	2.88E-08
MLXIPL	1.602197	5.701781	1.831361	0.001788	0.004159
MIR4285	0.0918	0.321154	1.806691	0.024269	0.039787
MIR101-2	0.168954	0.53947	1.674912	0.009894	0.018288
ADIPOQ	1.615048	0.526981	-1.61575	2.83E-08	4.02E-07
DMBX1	0.142766	0.699252	2.292165	2.70E-09	6.36E-08
MIR5697	0.036746	0.364003	3.308303	9.96E-05	0.000346
MCM8	1.361076	4.352033	1.676942	3.16E-12	1.40E-09

PENK	4.54293	0.643235	-2.82021	0.006563	0.012827
SMOC2	66.13318	21.45287	-1.6242	0.008383	0.015849
AC087284	0.325309	0.958044	1.558281	1.51E-06	1.03E-05
AKR1C1	10.52963	3.333319	-1.65942	3.84E-07	3.30E-06
VIP	16.97405	2.916114	-2.54121	4.44E-05	0.000175
FABP4	15.87788	4.637632	-1.77556	7.96E-09	1.48E-07
AL035252.	0.196952	0.705925	1.841667	9.05E-09	1.64E-07
AC004134	0.102335	0.427649	2.063136	4.95E-07	4.08E-06
CBWD4P	0.18409	0.532317	1.531873	2.50E-05	0.000108
ADAMTS1	0.504353	3.426724	2.764325	8.53E-12	2.04E-09
KRT24	1.56372	0.274873	-2.50815	4.01E-09	8.63E-08
AC008808	2.726476	0.850093	-1.68135	0.00035	0.001018
KRT7-AS	0.070722	1.048844	3.890502	2.50E-08	3.63E-07
AC022211	0.483485	1.54498	1.676045	6.74E-08	8.10E-07
PLIN1	3.041584	0.946813	-1.68367	1.49E-07	1.54E-06
RAD54L	0.894855	3.081424	1.783871	1.19E-09	3.38E-08
AP000487	0.084377	0.26458	1.648784	6.04E-05	0.000227
FOSB	95.20635	14.3947	-2.72552	0.003416	0.007289
CARTPT	11.60007	1.895212	-2.6137	0.000227	0.000703
AC105339	0.079409	0.250284	1.6562	1.47E-05	6.87E-05
OR5BA1P	0.04888	0.216511	2.147137	0.000711	0.001875
MDFI	3.666815	10.74976	1.551705	4.30E-07	3.62E-06
AC108860	0.403546	1.174168	1.540832	1.45E-07	1.50E-06
RF00093	0.034822	0.28177	3.016449	1.27E-05	6.11E-05
LINC0254	0.083833	0.56959	2.764342	2.40E-08	3.52E-07
TMEM270	0.070877	0.291585	2.04052	5.40E-05	0.000207
AC010789	0.151776	1.436128	3.242166	5.12E-06	2.86E-05
PGBD5	0.13874	0.482842	1.799167	0.000335	0.000979
AC020978	0.0726	0.265564	1.87102	6.41E-07	5.03E-06
AP000593	0.060344	0.257339	2.092387	2.33E-06	1.47E-05
EVA1A	0.240303	1.623638	2.756301	4.43E-09	9.27E-08
RNA5SP1	0.044895	0.247322	2.461771	0.000716	0.001887
PON1	0.041225	0.38596	3.22686	0.005634	0.011244
SFRP1	22.07367	4.312455	-2.35574	4.07E-09	8.71E-08
VCX	0.004281	0.521896	6.929787	3.80E-05	0.000154
KIF18B	1.330327	6.518794	2.292824	5.84E-11	4.46E-09
PSAPL1	25.20971	3.053206	-3.04558	0.002619	0.005795
ALPP	0.158369	2.09927	3.728525	6.34E-09	1.24E-07
RPL18P10	0.197247	0.622256	1.657502	4.22E-07	3.57E-06
LINC0257	0.057511	0.707241	3.620292	1.61E-09	4.27E-08
SERPINH1	16.3595	46.90937	1.519748	3.33E-11	3.27E-09
SLC2A4	9.157611	2.116182	-2.11351	1.57E-06	1.06E-05
C1QL1	7.61197	2.248946	-1.75902	2.17E-07	2.08E-06
MIR608	0.107102	0.443196	2.048953	0.000197	0.000622
TDRD6	0.111631	0.333542	1.579124	2.04E-06	1.31E-05
GPM6B	6.316997	1.132888	-2.47923	4.58E-07	3.81E-06
LINC0135	0.15077	0.496721	1.720088	6.57E-06	3.52E-05
SMIM5	2.151829	0.657467	-1.71057	3.52E-07	3.07E-06
GJB7	0.036703	0.283957	2.95172	0.004937	0.010036
MIR661	0.112388	0.419005	1.898477	0.001448	0.003476
IGLV1-50	13.77548	1.804362	-2.93254	0.026284	0.042553
CAB39L	10.18649	3.168399	-1.68483	5.21E-06	2.90E-05
AMPD1	1.993756	0.316176	-2.65669	0.002858	0.006241
PRR13P2	0.179837	0.546049	1.602338	7.06E-08	8.40E-07
RNU6-71	0.178707	0.593367	1.731327	0.00024	0.000737
RTN4RL2	0.75573	3.138731	2.054239	9.24E-07	6.84E-06
AC009237	0.310842	1.086087	1.804886	1.90E-06	1.24E-05
KRTAP5-5	0.029772	0.302733	3.346009	0.001063	0.002655

AC114737	0.091495	0.358751	1.971213	2.23E-05	9.79E-05
LINC0147	0.104924	0.351187	1.742892	0.030936	0.049041
RNU6-237	0.11112	0.446854	2.00768	7.96E-05	0.000286
RNU6-134	0.18516	0.771877	2.0596	3.51E-05	0.000144
GUCA2B	9.550743	0.768702	-3.63512	0.002848	0.006225
NDC80	1.257401	4.95749	1.979165	3.44E-11	3.31E-09
PROSER2-	0.080544	0.283973	1.817896	0.0001	0.000348
FLRT1	1.093771	0.289846	-1.91595	0.015362	0.026717
AC020891	0.043108	0.225756	2.388749	2.31E-05	0.000101
SOX14	0.130156	0.866889	2.73561	0.016421	0.028316
P2RY14	7.372499	2.083537	-1.82312	4.63E-09	9.61E-08
ANKRD18	0.182271	0.532177	1.545821	0.001145	0.002835
AC004837	0.127404	0.364062	1.514769	2.56E-07	2.37E-06
CTSV	1.625497	6.157275	1.921411	4.26E-05	0.00017
AL162413	0.491618	4.925171	3.324564	0.000901	0.002306
TM4SF19	0.100515	0.338358	1.751148	1.28E-06	8.96E-06
DLX6	0.048631	0.600029	3.625097	0.019468	0.032817
DMRTA1	0.8551	0.268524	-1.67104	3.93E-07	3.37E-06
PWAR5	0.990658	0.329071	-1.58999	0.000152	0.000499
RASGEF1A	0.327368	1.362641	2.057421	1.23E-05	5.94E-05
BANCR	0.036075	2.852437	6.305041	8.59E-05	0.000306
EGR4	0.135978	0.613005	2.172523	0.004764	0.009723
AC124067	0.284333	2.438384	3.100271	2.61E-06	1.62E-05
KNTC1	1.111252	3.737517	1.749894	3.28E-11	3.26E-09
AC011466	0.051821	0.26768	2.368897	4.79E-08	6.17E-07
AC000061	0.05851	0.863029	3.882643	0.000103	0.000356
CCNF	1.608866	4.859578	1.594787	1.72E-09	4.49E-08
HUNK	0.418994	1.415046	1.755849	0.015668	0.027185
MMP8	0.008814	0.228432	4.695777	2.25E-07	2.15E-06
AC034229	0.147684	0.430856	1.54469	0.000267	0.000807
LIPG	0.741304	2.612126	1.817088	3.70E-07	3.20E-06
AC092451	0.135547	0.393957	1.539247	0.005966	0.011812
POLQ	0.415303	1.803995	2.11896	7.13E-10	2.33E-08
TNXB	11.68715	3.669808	-1.67115	1.68E-05	7.72E-05
AL161725	0.071838	0.231469	1.688005	0.000526	0.001444
IGHG4	45.35539	249.5981	2.460261	0.000219	0.000679
AC007128	0.031266	0.566479	4.179343	5.41E-10	1.89E-08
MIR3682	1.079431	3.174449	1.556235	1.70E-05	7.79E-05
AC068491	0.089758	0.808857	3.171767	3.45E-10	1.36E-08
NKILA	0.31205	1.382473	2.1474	1.19E-06	8.45E-06
LINC0253	0.077646	1.930369	4.635828	0.000308	0.000912
AL356417	0.035477	0.271891	2.938088	3.00E-08	4.24E-07
TPM1	87.59655	26.73963	-1.71189	0.008929	0.016719
WDR72	0.3245	3.439294	3.405824	3.56E-07	3.10E-06
C20orf204	0.092241	0.813438	3.140554	5.31E-07	4.32E-06
ZHX1-C8	0.168602	0.492915	1.547715	8.84E-11	5.71E-09
CIT	0.97697	2.765115	1.500953	1.14E-10	6.59E-09
AC023389	0.235454	0.851014	1.853737	4.19E-08	5.54E-07
FNDC11	0.11965	0.364515	1.607157	3.43E-05	0.000141
AL139128	0.065761	0.21431	1.704397	7.06E-07	5.46E-06
INHBA	1.020371	9.136558	3.162557	3.11E-12	1.40E-09
PCDH10	0.726607	0.235735	-1.62401	0.009573	0.017759
AC112484	0.233302	1.072983	2.201355	0.000109	0.000374
MTND2P4	0.084931	0.280093	1.721552	2.94E-06	1.79E-05
AC092794	0.074832	0.26521	1.825414	0.008153	0.015474
AC006329	1.098018	3.58072	1.705348	2.32E-05	0.000101
PAX8	0.583495	1.690121	1.534335	7.15E-06	3.77E-05
KLK4	0.024578	0.405705	4.045017	0.011315	0.020501

RNA5SP49	2.872002	8.469514	1.560222	0.015261	0.026564
STIL	1.095445	4.53035	2.048106	4.45E-11	3.89E-09
AC004830	0.126367	0.367819	1.541372	2.54E-05	0.000109
MIR4292	1.557233	4.640236	1.575213	5.06E-07	4.14E-06
SEZ6L2	8.478221	26.45326	1.641612	1.08E-06	7.80E-06
AC009041	0.114574	0.448083	1.967482	1.95E-09	4.94E-08
FMO2	6.928658	2.130056	-1.70168	0.013727	0.024235
SIX3	0.030478	0.67963	4.478889	7.41E-05	0.000269
RNU6-10F	0.197454	1.311383	2.731498	2.43E-05	0.000105
CXCL8	4.784959	46.47494	3.279874	1.29E-07	1.38E-06
AC108676	0.040453	0.33265	3.039703	3.09E-08	4.34E-07
RNU6-669	0.131621	0.679136	2.367308	1.95E-06	1.26E-05
AC244093	0.060911	0.217158	1.833961	4.56E-05	0.00018
AC103691	0.857443	2.902852	1.759358	0.00012	0.000407
LINC0169	0.055863	0.394914	2.821565	0.003442	0.00734
TEDC2	0.623567	2.097307	1.74992	1.72E-09	4.49E-08
KCNMA1	9.854625	3.268621	-1.59212	0.000481	0.001337
CKS1BP2	0.054893	0.230629	2.070875	1.45E-06	9.88E-06
AHSG	0.042769	1.392268	5.024717	0.009178	0.017139
ADAMTS9	0.708839	0.192435	-1.88109	8.36E-06	4.31E-05
AC009961	0.313486	0.908265	1.534711	0.001491	0.003566
TRBV29OF	0.238781	0.798772	1.742092	4.82E-05	0.000188
AL078459.	0.093264	0.297737	1.674652	4.98E-09	1.02E-07
TAF9P3	0.239548	0.728611	1.604836	4.40E-05	0.000174
NEFL	1.975529	0.253757	-2.96072	5.47E-05	0.000209
PGM5-AS	11.68235	1.138625	-3.35897	1.95E-08	3.00E-07
PDLIM3	33.11552	10.65727	-1.63567	0.001298	0.00316
AP000525	0.027827	0.380803	3.77447	3.26E-08	4.55E-07
AC246787	0.726036	0.247345	-1.55352	0.022699	0.037522
LRAT	0.763685	0.167889	-2.18547	0.000107	0.000369
HOXA10	0.515008	4.220221	3.034653	2.15E-08	3.23E-07
LINC0225	0.029379	1.745223	5.892497	0.000187	0.000593
AL021807.	0.328138	1.137927	1.794034	0.000236	0.000725
ABCA13	0.03706	0.236397	2.673268	2.11E-05	9.34E-05
SNORD56	0.10603	0.39085	1.882143	0.010998	0.020005
PPIAP3	0.144728	0.439918	1.603892	1.41E-07	1.47E-06
GTF2IP13	0.74422	2.181237	1.551345	1.96E-07	1.92E-06
AL078621.	0.035812	0.252899	2.820045	0.013721	0.024235
PAEP	0.052277	1.333909	4.673347	6.13E-06	3.33E-05
RNU6-288	0.129012	0.366443	1.50609	0.004181	0.008689
AL132780.	0.08414	0.297958	1.824247	9.71E-09	1.73E-07
CORO6	1.132461	0.371291	-1.60884	0.022021	0.036531
CHODL	1.463478	0.255398	-2.51858	8.43E-09	1.55E-07
AC091390	0.218722	0.70869	1.696056	7.99E-06	4.15E-05
MAGEA12	0.007085	3.916119	9.110534	1.35E-05	6.41E-05
DLGAP1-A	0.351157	1.973526	2.490586	4.75E-11	3.96E-09
PPIAP45	0.056887	0.349926	2.620879	7.85E-07	5.98E-06
ARGFXP2	0.101521	0.347863	1.776743	1.03E-07	1.15E-06
AL161891.	0.121115	0.517964	2.096481	1.38E-11	2.40E-09
ADAMTS1	24.82741	7.658505	-1.6968	0.000104	0.00036
MIR548N	0	0.316618	Inf	4.65E-06	2.63E-05
STPG4	0.054822	0.298394	2.444405	2.52E-08	3.65E-07
SNORA5C	0.818363	2.468523	1.592835	8.49E-09	1.56E-07
CDCA2	1.025327	4.132053	2.010775	8.54E-10	2.69E-08
AC008147	0.308534	0.908624	1.558253	3.09E-06	1.86E-05
HSD17B3	0.080402	0.273766	1.767633	0.000653	0.001739
MIR17HG	0.123246	0.547173	2.150455	4.13E-08	5.48E-07
AC022211	0.394735	1.239396	1.650682	3.86E-08	5.19E-07

ERCC6L	0.491441	1.702998	1.792986	1.44E-09	3.92E-08
AC068580	0.039992	1.33716	5.063312	5.97E-08	7.33E-07
AC009237	0.332771	1.016366	1.610817	2.52E-05	0.000109
HSPB8	72.64623	13.57349	-2.4201	2.47E-07	2.30E-06
PAGE1	0.036822	1.423903	5.273125	0.003926	0.008226
AL365181.	1.78005	5.73971	1.68906	9.87E-06	4.94E-05
MAGEA4	0.006467	5.940371	9.843313	0.000759	0.001984
AC104534	1.920571	5.966383	1.635321	0.000321	0.000946
CSRNP1	43.16812	13.82142	-1.64306	3.46E-05	0.000142
TRIB3	2.100385	10.59259	2.33433	4.19E-07	3.55E-06
AL590133.	0.097228	0.327521	1.752148	2.72E-07	2.50E-06
RN7SKP78	0.211135	0.603707	1.515681	0.000142	0.000471
RN7SL614	0.111679	0.322519	1.530033	1.49E-05	6.96E-05
DTL	1.289931	5.35591	2.053838	4.04E-11	3.65E-09
RNU6-12(	0.11384	0.585779	2.363354	7.41E-06	3.89E-05
CILP2	0.181221	1.341449	2.887971	2.30E-08	3.42E-07
IGKV1OR1	1.267248	0.25231	-2.32843	0.010363	0.019033
PPIAP58	0.060981	0.266635	2.128442	3.37E-08	4.66E-07
TIMELESS	2.584009	8.137409	1.654959	1.85E-10	8.91E-09
GABRD	0.151276	1.426841	3.237566	2.50E-13	1.20E-09
ASH1L-IT1	0.107059	0.326318	1.607878	2.43E-05	0.000105
AC005550	0.603356	1.711907	1.504524	0.010833	0.019744
PLP1	4.120889	0.429155	-3.26338	7.57E-10	2.45E-08
PYGM	4.809446	0.804979	-2.57885	1.24E-06	8.69E-06
NALT1	3.312091	0.70803	-2.22586	0.000547	0.001494
GPRC5A	20.51698	77.44882	1.916425	5.48E-08	6.86E-07
C8orf88	7.268795	1.967678	-1.88522	9.18E-05	0.000323
SSTR5	0.133319	0.880997	2.724251	0.007011	0.013576
LINC0098:	3.018202	0.980757	-1.62172	0.005057	0.01023
CATSPERE	0.206221	0.930358	2.173593	7.15E-06	3.77E-05
RNU6-83:	0.079144	0.264944	1.743132	0.008401	0.015878
AC244205	1.189282	0.271761	-2.12968	0.000271	0.000817
MIR644A	0.127189	0.546751	2.103909	6.24E-05	0.000233
AC002128	0.202873	0.619608	1.61078	8.19E-09	1.52E-07
AL928654.	0.756202	2.196826	1.538577	8.62E-08	9.90E-07
RNA5SP82	0.648923	1.855815	1.515935	6.81E-06	3.63E-05
MAD2L1	1.633871	6.077862	1.89527	5.77E-10	1.98E-08
AL078644.	0.115208	0.330716	1.521349	6.52E-08	7.88E-07
LINC0133(	1.762144	0.431806	-2.02888	4.40E-07	3.69E-06
AC097639	0.077489	0.293428	1.920951	0.000724	0.001903
AC073585	0.149193	0.808618	2.438274	7.80E-08	9.13E-07
ZFPM2-AS	0.105701	0.657752	2.637555	0.000405	0.001152
MIR613	0.07081	0.308043	2.121111	0.007445	0.0143
NPM1P9	0.051664	0.219558	2.087371	2.64E-10	1.14E-08
GRIK5	2.845921	0.912424	-1.64112	0.001717	0.004013
MYADML2	0.07128	0.33163	2.218006	0.00033	0.000968
AC116348	0.078083	0.295908	1.922064	0.000159	0.000518
CPNE7	0.726728	3.109432	2.097164	1.26E-05	6.06E-05
IQGAP3	2.39024	10.62794	2.152635	2.97E-11	3.11E-09
KPNA2	15.02783	43.45274	1.53181	2.19E-10	1.00E-08
AC116562	0.052585	0.305187	2.536957	0.003072	0.006644
RNU7-181	0.284854	1.681359	2.561331	6.94E-08	8.27E-07
AL139099.	0.094008	0.27151	1.530159	1.22E-05	5.89E-05
RPL22P12	0.13617	0.419003	1.621548	4.53E-09	9.44E-08
TBX15	0.100273	0.314896	1.650937	6.80E-05	0.000251
ALG1L	0.735731	2.12955	1.533299	0.000926	0.00236
LARGE2	3.808192	11.54648	1.600275	0.000836	0.002158
SCNN1G	2.717228	0.474327	-2.51818	2.48E-07	2.31E-06

MIR3125	0.146122	0.57003	1.963866	0.005935	0.01176
MIR2116	0.263608	0.931477	1.821124	3.93E-05	0.000159
AC005828	0.056787	0.257974	2.183599	2.90E-05	0.000122
RNASE2	0.5934	1.855076	1.644402	2.30E-05	0.0001
ARSE	2.137398	8.688991	2.023333	1.28E-06	8.96E-06
BVES	5.117422	1.808655	-1.5005	0.002312	0.005192
LINC0089(	0.112748	0.330705	1.552443	7.75E-05	0.000279
TEAD4	4.100723	12.36822	1.592688	1.55E-11	2.48E-09
OCLM	0.244178	0.708298	1.536423	8.39E-08	9.70E-07
PURPL	0.030804	0.562775	4.191372	0.000193	0.000611
SORBS1	52.35603	16.40544	-1.67418	0.000133	0.000444
LINC0062	0.065745	0.227832	1.793014	8.19E-09	1.52E-07
AC108463	0.203732	0.92053	2.175791	3.28E-11	3.26E-09
MIR1-1H(	1.49584	0.32842	-2.18734	9.23E-06	4.68E-05
RNU6-418	0.416671	1.224707	1.555456	1.09E-05	5.38E-05
IGKV1OR2	3.458566	0.386815	-3.16046	0.025782	0.041896
PTPRU	2.573254	8.36623	1.700984	4.35E-05	0.000173
AC091133	0.009079	0.273722	4.913964	6.98E-06	3.69E-05
AC012676	0.339782	0.982082	1.531235	1.46E-08	2.36E-07
AC008114	0.047325	0.29973	2.662978	5.32E-08	6.70E-07
AC120498	0.104862	0.812401	2.953697	7.30E-05	0.000266
GTF2IP5	0.144289	0.47819	1.728626	4.04E-07	3.44E-06
RNU6-558	0.172581	0.747802	2.115386	0.001045	0.002619
PITX1	68.18611	21.69115	-1.65237	0.015463	0.026866
AC106875	0.001288	0.304825	7.886878	3.00E-05	0.000126
CYP27B1	0.302458	1.422092	2.233207	8.67E-10	2.70E-08
CKB	177.9071	42.94383	-2.0506	7.69E-07	5.88E-06
AP001628	0.184987	0.775837	2.068331	1.71E-07	1.72E-06
IGLV3-32	1.260702	0.175022	-2.84862	0.006288	0.012357
AC004584	0.078406	0.222963	1.507763	1.62E-05	7.49E-05
MIR4713H	0.121739	0.578191	2.247754	1.28E-05	6.16E-05
AL022322.	0.394487	1.405781	1.833321	1.01E-09	2.99E-08
AC005393	0.104376	0.313358	1.586017	0.000405	0.001151
CHST4	0.097684	1.398105	3.839201	6.04E-06	3.28E-05
MIR4698	0.11111	0.324607	1.546704	0.019227	0.032447
CLCN4	0.718326	2.517339	1.80919	4.05E-06	2.35E-05
HIST2H2B	0.270334	0.769259	1.508726	2.64E-06	1.64E-05
POLR3GP1	0.125488	0.660539	2.396098	6.87E-06	3.65E-05
LINC0210(	0.221865	0.739744	1.737345	7.37E-06	3.87E-05
ITGA11	1.482274	4.666057	1.654391	6.01E-07	4.77E-06
APOA1	288.9574	46.55286	-2.63392	0.026529	0.042888
ARL4D	8.10838	2.434109	-1.73602	5.18E-05	0.000199
AC104365	0.04356	0.256226	2.55635	9.05E-05	0.00032
ALOX12P2	0.076706	0.312583	2.026834	0.000274	0.000823
TCAM1P	0.012802	0.310219	4.598853	3.27E-07	2.91E-06
SNRPGP9	0.119171	0.4085	1.777299	4.70E-05	0.000184
TGFB2-AS	0.454789	1.303763	1.519411	1.74E-05	7.93E-05
AC009065	2.939635	8.458815	1.524819	8.74E-06	4.46E-05
PGAM1P7	0.110954	0.428734	1.950114	8.90E-07	6.63E-06
IGKV1OR-	1.61842	0.114633	-3.8195	0.018412	0.031268
AC117386	0.02756	0.293796	3.414167	1.80E-05	8.16E-05
SDS	0.922331	4.86496	2.399071	3.82E-10	1.47E-08
IGKV1OR2	3.121301	0.527425	-2.56511	0.017994	0.030633
FARSA-AS	0.124429	0.401292	1.689332	7.50E-07	5.76E-06
OTC	14.58935	2.919623	-2.32106	0.027612	0.044461
MAGEA3	0.017298	7.941312	8.842635	6.64E-05	0.000245
ADRB2	2.161302	0.620559	-1.80026	1.71E-10	8.37E-09
CTHRC1	3.195868	25.5544	2.999292	3.50E-12	1.42E-09

TDRD5	0.095348	0.828331	3.11894	3.07E-06	1.85E-05
PCDHB8	0.232525	0.961162	2.047394	0.001088	0.002712
TIGD1	0.680284	1.964852	1.530211	7.54E-11	5.21E-09
RNA5SP21	0.179696	0.592689	1.721716	0.00012	0.000407
AC005534	0.046142	0.277916	2.590504	6.99E-09	1.34E-07
ERFE	0.134262	0.762851	2.506355	6.17E-09	1.21E-07
Z97200.1	0.110733	0.32675	1.561106	0.00043	0.001214
SAGE1	0.003641	0.444825	6.93289	0.000325	0.000955
RNU6-856	0.275007	0.846958	1.622823	1.46E-05	6.86E-05
AK4P3	0.076682	0.284288	1.890404	0.000649	0.00173
CHTF18	1.272319	3.638506	1.515886	1.62E-09	4.29E-08
TLX1	0.249651	1.414605	2.502413	2.84E-05	0.00012
SLC16A9	2.800412	0.874761	-1.67868	0.00016	0.000519
BEND5	2.096263	0.73847	-1.50521	9.87E-06	4.94E-05
CCNB2	4.643946	13.44182	1.533305	4.43E-09	9.27E-08
DDX11-AS	0.100261	0.33178	1.726464	5.58E-09	1.12E-07
NALCN	0.098009	0.29006	1.565369	0.005565	0.011121
ITGAX	1.242637	4.056426	1.706804	1.52E-08	2.44E-07
TRAJ36	0.062504	0.483457	2.951363	0.001055	0.002641
WDHD1	0.854509	2.629966	1.621876	8.56E-11	5.65E-09
CEP72	0.912397	3.272265	1.842556	9.12E-11	5.85E-09
POLR2KP1	0.159216	0.560808	1.816517	3.80E-05	0.000154
CDC25C	0.858895	2.678612	1.640932	9.31E-09	1.68E-07
NPPC	2.565739	0.404066	-2.66671	2.28E-07	2.16E-06
CDC7	0.979051	3.416978	1.803264	1.09E-10	6.42E-09
AL512444.	0.048335	0.211682	2.13075	2.41E-05	0.000105
SLC1A5	16.43892	50.15324	1.609228	5.34E-09	1.08E-07
UAP1L1	1.692701	4.825666	1.511401	5.19E-09	1.06E-07
LINC01522	0.054222	0.835154	3.94509	0.001371	0.003314
AP001065	1.192452	0.368038	-1.69601	0.004453	0.009163
CASC19	0.536379	2.952225	2.460479	6.53E-06	3.50E-05
AC130456	0.065514	0.270111	2.043668	5.10E-07	4.18E-06
KCNE2	51.78906	2.91177	-4.15268	0.000545	0.001488
MYO3B	0.047046	0.221604	2.235846	0.020413	0.034203
FGF3	0.0215	2.255139	6.712726	0.000347	0.001009
AIFM3	0.125802	0.366233	1.541611	5.56E-05	0.000212
MYZAP	3.460845	1.065344	-1.6998	1.79E-08	2.79E-07
HASPIN	0.858734	2.798423	1.704331	4.07E-09	8.71E-08
MCM2	4.311475	15.02134	1.80076	9.42E-11	5.97E-09
RNA5SP47	0.084651	0.307977	1.863227	0.000213	0.000664
PGA4	8.174651	0.458845	-4.15508	0.000195	0.000617
CDK6	3.298799	14.20317	2.1062	9.17E-09	1.66E-07
AC138409	0.268114	0.85228	1.668481	2.14E-07	2.06E-06
AC007619	0.076064	0.275924	1.858985	5.13E-06	2.86E-05
AC004231	0.020233	0.387514	4.259499	0.000768	0.002004
NTN1	15.54856	5.361728	-1.53601	9.76E-06	4.90E-05
MYLK	59.69017	19.1797	-1.63791	0.001621	0.003821
FZD2	1.88611	5.462008	1.534018	4.76E-06	2.69E-05
RNU1-122	0.115185	0.402824	1.806192	0.00015	0.000492
ZBTB16	4.107073	0.895589	-2.1972	1.08E-07	1.19E-06
CST2	0.528435	6.66205	3.656168	1.91E-09	4.85E-08
RFC3	2.919801	11.22366	1.942601	2.62E-12	1.40E-09
BCHE	5.49851	1.100923	-2.32033	5.15E-06	2.87E-05
OR2B6	0.030238	0.284709	3.235044	1.57E-07	1.61E-06
AC010627	0.049116	0.216764	2.141858	0.00234	0.005247
PRC1	2.764095	10.07079	1.865298	1.33E-10	7.32E-09
AC022973	0.190221	0.717762	1.915831	7.40E-08	8.73E-07
AL162595.	0.224438	0.749983	1.740539	2.33E-10	1.04E-08

AC005089	0.122338	0.599103	2.291926	9.08E-06	4.61E-05
C3P1	0.036472	0.465849	3.674982	4.10E-08	5.45E-07
YES1P1	0.075877	0.228472	1.590279	1.53E-06	1.04E-05
HOXA13	0.637903	4.908899	2.94399	1.79E-08	2.79E-07
AC019155	0.012304	0.265301	4.430476	0.000653	0.00174
INA	1.302341	0.321585	-2.01784	8.59E-07	6.44E-06
PI16	27.0943	3.208093	-3.0782	6.17E-09	1.21E-07
RDM1	0.218143	0.773316	1.825787	7.16E-08	8.48E-07
ELOVL3	0.117404	0.396251	1.754931	2.35E-05	0.000102
PRSS33	0.089951	1.377865	3.937154	6.27E-05	0.000234
ABI3BP	20.32698	4.307913	-2.23833	4.17E-05	0.000167
RNASEH1I	0.013944	0.239167	4.100255	0.001327	0.003224
RN7SL809	0.159197	0.643256	2.014582	2.71E-08	3.89E-07
AL158825.	0.305301	1.039924	1.768176	8.50E-06	4.37E-05
EPHB2	1.853803	8.761793	2.240738	1.05E-07	1.17E-06
AL161729.	0.15045	0.488669	1.699572	1.07E-05	5.30E-05
PADI2	1.112961	4.244159	1.931075	0.000197	0.000622
PVT1	0.84144	3.55384	2.078447	2.85E-10	1.20E-08
AC116407	1.030746	0.329982	-1.64323	0.008743	0.016421
AL021331.	0.051417	0.218267	2.085775	0.000482	0.001338
RN7SL449	0.073654	0.212539	1.528889	0.000237	0.00073
HOXC9	0.121671	2.340379	4.265683	6.69E-12	1.99E-09
GRIK3	0.920802	0.203877	-2.17519	3.41E-06	2.02E-05
PCP2	0.176665	0.502195	1.507228	0.00032	0.000942
AL161729.	0.094348	0.422204	2.161877	1.89E-05	8.51E-05
ROS1	0.017069	0.28818	4.077553	3.95E-06	2.30E-05
PCMTD1P	0.063739	0.300162	2.235497	1.38E-05	6.55E-05
RNU6-123	0.102873	1.6464	4.000376	0.000122	0.000411
MMP10	0.205965	2.625911	3.672345	3.68E-07	3.19E-06
MYH7B	0.21136	0.838678	1.988417	0.000151	0.000495
TCEAL7	4.489326	1.569667	-1.51604	0.002084	0.004749
PLPPR4	0.339998	1.028329	1.596705	5.79E-05	0.000219
HOXA10-/	0.086499	0.677001	2.968399	3.92E-08	5.25E-07
RPL39P39	0.108593	0.330373	1.605159	0.000156	0.000509
SPP1	11.78024	85.34403	2.856921	3.33E-08	4.61E-07
MIR3153	0.176181	0.992218	2.4936	1.28E-06	8.98E-06
KIF26B	0.433037	2.63746	2.606587	4.24E-11	3.77E-09
AL133410.	0.215634	0.765851	1.828476	6.41E-10	2.15E-08
AMH	0.259317	1.867736	2.848503	2.46E-06	1.54E-05
ATP5MC1	0.230739	1.140973	2.305929	4.87E-09	1.00E-07
MPPED2	0.520934	0.183327	-1.50668	0.009573	0.017759
RNU6-322	0.459203	1.460001	1.668766	0.000194	0.000615
TRPM5	0.123062	0.386275	1.650248	0.009571	0.017759
DUSP9	0.046419	0.899999	4.277152	0.000201	0.000632
MIR3133	0.045323	0.283149	2.643235	0.000535	0.001466
PROC	0.423036	2.01113	2.249153	0.000223	0.00069
WDR62	0.490054	2.104542	2.102493	8.04E-11	5.40E-09
SCGB2A1	17.5311	2.458738	-2.83393	0.020239	0.033974
AP002784	0.011772	0.643771	5.773087	8.35E-08	9.67E-07
AC091179	0.033601	0.46853	3.801579	1.36E-05	6.46E-05
MIR5094	0.466845	1.780504	1.931271	1.42E-05	6.69E-05
MSLNL	0.05716	0.441268	2.948576	6.58E-07	5.15E-06
RNF43	3.01026	10.028	1.736074	4.39E-05	0.000174
AL390729.	0.418204	1.308287	1.645399	2.30E-05	0.0001
AC087385	0.298774	0.918646	1.620453	3.14E-06	1.89E-05
DEPDC1B	1.220338	4.917848	2.010747	4.25E-10	1.60E-08
LRFN4	4.286068	15.94697	1.895556	6.23E-11	4.54E-09
AL590438.	0.064709	0.307144	2.246873	0.000115	0.000392

MIR6832	0.117619	0.41625	1.823328	0.00205	0.004683
IGKV1OR2	3.735814	0.394099	-3.24479	0.010081	0.018579
AC106028	0.090533	0.377906	2.061507	4.30E-08	5.67E-07
RNU6-117	0.150709	0.472026	1.647104	6.17E-05	0.000231
GKN2	955.299	45.69557	-4.38583	0.000224	0.000695
GFY	0.010135	0.364856	5.169871	0.000181	0.000578
GPR158	0.10767	0.611573	2.505913	0.000479	0.001332
ESM1	0.158903	2.937778	4.208508	1.36E-13	1.20E-09
AC025423	0.109626	0.428017	1.965077	1.95E-06	1.26E-05
CDCA7	5.681787	19.00116	1.741671	1.15E-07	1.26E-06
SLC18A3	1.102896	0.172205	-2.6791	7.40E-05	0.000269
AC016949	0.366326	1.081425	1.561732	1.11E-07	1.22E-06
MAGEA8	0.013203	0.247901	4.230882	0.016474	0.028402
RHOB	423.8236	137.7717	-1.62118	0.006119	0.012071
AC022973	0.095364	0.470866	2.303796	2.51E-08	3.65E-07
KIF4A	1.407868	5.9895	2.088924	9.27E-11	5.91E-09
AC017006	0.101093	0.294494	1.542554	0.000169	0.000542
APOBEC2	4.311919	1.181667	-1.86751	5.09E-06	2.85E-05
NPTX1	5.24862	1.212113	-2.11441	0.003138	0.006762
AL365181.	0.35141	1.566426	2.156249	1.56E-05	7.24E-05
AC130456	0.752911	2.366913	1.652455	3.11E-06	1.87E-05
CCR8	0.161647	0.597531	1.886166	1.61E-07	1.65E-06
AC079684	0.173824	0.682662	1.973547	8.86E-09	1.61E-07
AP003696	0.061581	0.22098	1.843368	9.57E-05	0.000334
OSTCP8	0.043876	0.289164	2.720369	2.06E-09	5.13E-08
C5orf46	0.128164	0.48669	1.925012	4.30E-07	3.62E-06
TPM3P9	0.978872	2.959142	1.595986	3.28E-10	1.31E-08
UCA1	1.330109	12.88099	3.275628	0.000918	0.002342
AC087241	0.072011	0.227922	1.662259	0.001045	0.002619
MIR3198-	0.157687	0.661552	2.068792	2.50E-06	1.57E-05
AC087491	0.38862	1.12594	1.534699	0.024372	0.039913
RDH12	4.895343	0.766843	-2.67441	7.99E-06	4.15E-05
IGLV3-24	1.254585	0.200884	-2.64277	0.024646	0.040282
GJA3	0.121528	0.423077	1.799629	0.00049	0.001356
DCSTAMP	0.051903	0.289541	2.479879	3.13E-10	1.27E-08
SATB2	0.401326	1.372129	1.773569	4.30E-07	3.62E-06
ADRB3	0.652747	0.177144	-1.8816	2.79E-06	1.72E-05
SNHG4	0.577572	1.65253	1.516604	8.55E-09	1.56E-07
COL3A1	78.37401	350.0488	2.159109	1.36E-09	3.75E-08
MYBL2	8.752795	39.00175	2.155723	9.77E-10	2.95E-08
IL17RB	2.770569	8.772599	1.662822	1.12E-08	1.92E-07
MTTP	28.46248	1.576223	-4.17452	0.003744	0.007895
RNU6-571	0.06516	0.210166	1.689469	0.003051	0.006606
TRIM31-A	0.565164	2.189446	1.953824	1.98E-07	1.95E-06
AC113143	0.313292	1.145011	1.86978	5.06E-06	2.83E-05
HMGB3	3.368048	12.66033	1.91033	6.64E-11	4.79E-09
RNY3P12	0.065351	0.303023	2.213158	0.000937	0.002385
TRABD2A	0.862917	3.181757	1.88253	1.50E-05	6.98E-05
AL391095.	0.082603	0.235746	1.512962	6.98E-05	0.000256
MIR4728	0.45091	2.965784	2.717501	0.001356	0.003283
AC022144	0.147179	0.978907	2.733597	5.99E-09	1.18E-07
AC004678	0.096029	0.300538	1.646008	1.67E-08	2.63E-07
CCDC150	0.057107	0.321123	2.491384	3.38E-11	3.29E-09
CFL2	17.47198	5.88425	-1.57011	0.002904	0.006325
RNU6-104	0.258019	0.787729	1.610222	0.000356	0.001032
NKX6-2	9.767817	0.742919	-3.71676	6.11E-07	4.83E-06
AL096828.	0.130714	0.559334	2.097293	9.96E-08	1.12E-06
SULF1	5.554116	27.51268	2.308468	2.79E-09	6.50E-08

HMGB2P1	0.065315	0.257105	1.976873	4.75E-06	2.69E-05
SLC6A7	0.061845	0.254552	2.041226	7.08E-05	0.000259
AL121994.	0.088763	0.28324	1.673998	7.18E-06	3.78E-05
FOXS1	0.72849	4.479458	2.620343	1.71E-11	2.48E-09
FRAS1	0.572944	1.849356	1.690556	0.006164	0.012146
LINC0034(	0.244128	0.787673	1.689961	1.41E-06	9.68E-06
RN7SL481	0.195734	0.554536	1.502391	7.01E-06	3.71E-05
LY6H	1.405981	0.374634	-1.90802	5.15E-06	2.87E-05
AC007991	0.113665	0.775594	2.770514	3.35E-06	1.99E-05
LINC0060!	0.097406	0.419039	2.105006	0.018421	0.031272
LINC0114(	0.034465	0.355411	3.366265	9.14E-08	1.04E-06
CRYAB	39.30434	8.426193	-2.22174	3.33E-08	4.61E-07
AC008083	0.050716	0.226774	2.160735	0.000119	0.000403
LINC0161!	0.082862	0.419315	2.339251	5.42E-09	1.10E-07
RPL31P50	0.012985	0.221525	4.092561	2.24E-06	1.42E-05
UCN2	0.079133	0.450011	2.507612	3.75E-08	5.07E-07
LINC0138!	0.368772	1.541502	2.063535	7.53E-09	1.42E-07
AL512430.	0.103913	0.310792	1.580575	0.00011	0.000378
SCN8A	0.139218	0.488135	1.809933	0.000942	0.002393
RCAN2	22.16766	7.443847	-1.57434	2.78E-05	0.000118
AC008870	0.488903	1.935429	1.985033	5.50E-05	0.00021
AC125257	2.299135	6.583383	1.517738	1.85E-10	8.91E-09
MYOM3	0.140448	1.139566	3.020377	1.57E-06	1.06E-05
XRCC2	0.384139	2.058059	2.421586	2.00E-12	1.40E-09
VPS9D1-A	1.776068	6.991938	1.977005	2.40E-08	3.51E-07
TMEM100	7.550688	1.227916	-2.6204	9.45E-08	1.07E-06
COL11A1	0.590633	3.532648	2.580415	3.49E-10	1.37E-08
AL596247.	0.230635	0.671362	1.541478	0.000126	0.000422
SPOCD1	0.142534	0.513756	1.849773	5.06E-08	6.44E-07
AL121829.	0.066843	0.210418	1.654419	0.001515	0.003612
AC090695	0.088188	0.251126	1.509763	4.81E-06	2.71E-05
RNU6-403	0.10716	0.581847	2.440868	0.000302	0.000898
SNORD12	0.120001	0.53172	2.147617	0.000102	0.000353
AL356740.	0.084985	0.744782	3.131533	4.52E-06	2.58E-05
AL109615.	0.157143	1.387151	3.141973	8.67E-10	2.70E-08
AL355472.	0.056705	0.223192	1.976742	0.001145	0.002836
RNU6-19!	0.383661	1.512212	1.978758	7.64E-07	5.85E-06
SNRPCP1!	0.052495	0.293727	2.484222	0.000214	0.000668
MTHFD1L	1.703095	6.504808	1.933348	6.26E-13	1.31E-09
C4BPB	1.241685	3.805999	1.615975	0.00107	0.002672
PRSS30P	0.080444	0.367694	2.192456	2.58E-06	1.60E-05
DCAF13	2.074796	5.921706	1.513043	3.22E-12	1.40E-09
HAMP	0.092722	0.373319	2.009418	1.61E-06	1.08E-05
DDN	0.075685	0.260747	1.784577	0.02987	0.047551
COL12A1	7.932074	25.57914	1.689198	1.74E-07	1.75E-06
AC124944	0.143236	0.548697	1.93762	2.68E-08	3.85E-07
AC006480	0.068513	0.261519	1.932466	4.49E-07	3.75E-06
SYNGR4	0.104677	1.326981	3.664128	1.32E-05	6.30E-05
AC005993	0.025995	0.714241	4.780093	6.61E-06	3.53E-05
TCAP	0.258899	0.885119	1.773483	0.000741	0.001942
AL669831.	0.087641	0.258511	1.560553	1.90E-08	2.92E-07
ENC1	5.648648	24.20413	2.099276	1.95E-11	2.63E-09
AL035420.	0.113039	0.383257	1.761487	0.000254	0.000773
AC007038	0.394611	1.182227	1.583003	9.08E-08	1.03E-06
COL4A6	5.87453	0.90611	-2.69672	1.94E-06	1.26E-05
TENT5B	19.04316	3.734129	-2.35043	0.003604	0.007635
MIR4729	0.070972	0.366017	2.366584	0.002739	0.006017
SAPCD1	0.081899	0.403996	2.30243	2.27E-08	3.38E-07

AC068790	0.151356	0.435441	1.524537	7.10E-07	5.49E-06
LINC0105(	0.00711	0.233041	5.034567	1.18E-10	6.69E-09
AC073107	0.396296	1.284439	1.696489	1.21E-07	1.31E-06
FAM227A	0.075898	0.262714	1.79135	1.87E-08	2.88E-07
SYPL1P2	0.095184	0.358141	1.911734	2.24E-07	2.14E-06
PUS7	1.881061	6.471144	1.782474	2.62E-12	1.40E-09
FOXD1	0.640338	2.680415	2.06555	0.018548	0.03145
CDIPTOSF	0.109026	0.434656	1.995204	0.000586	0.001587
CRTAC1	1.478078	0.466478	-1.66384	1.65E-05	7.57E-05
LINC0206{	0.087813	0.266241	1.60023	0.000526	0.001443
MIR4284	0.142785	0.547606	1.939292	0.000711	0.001875
LEMD1	0.20735	1.183783	2.513267	1.48E-08	2.39E-07
PRAME	0.041922	4.00366	6.577468	2.01E-07	1.96E-06
AURKA	4.170273	14.56164	1.803959	1.96E-09	4.94E-08
AC010547	0.053179	1.188017	4.48156	8.90E-06	4.53E-05
MIR567	0.065474	0.343391	2.390865	0.000638	0.001706
KCNMB1	16.92111	4.468655	-1.92091	6.46E-06	3.47E-05
RNU6-562	0.050575	0.337703	2.739253	0.000638	0.001706
CABP4	0.162766	0.488922	1.586805	0.01843	0.031272
LINC0197!	0.113514	0.451611	1.992209	1.18E-07	1.28E-06
REP15	4.273598	0.894394	-2.25647	0.008294	0.015717
LYNX1	16.27182	4.711442	-1.78813	0.001017	0.002555
MIR3939	0.145593	0.58563	2.008051	7.14E-05	0.000261
XPO5	3.472317	9.837678	1.502419	6.86E-12	1.99E-09
CPA2	21.15958	2.40273	-3.13856	0.010578	0.019353
CSF2	0.058713	0.997896	4.087149	1.50E-08	2.42E-07
EPOP	0.991978	3.246855	1.710662	1.67E-06	1.11E-05
AC044840	0.073084	0.284466	1.960632	6.10E-06	3.32E-05
HIST1H3J	0.053947	0.257608	2.255568	7.42E-08	8.75E-07
RPS26P45	0.056742	0.288677	2.346971	8.05E-06	4.17E-05
AL136221.	0.245235	0.720203	1.554239	5.57E-06	3.08E-05
CNTNAP2	0.385791	1.987401	2.364992	0.001595	0.00377
CCKBR	4.145551	0.505729	-3.03513	2.87E-07	2.61E-06
AC005021	0.051587	0.348987	2.75808	4.71E-07	3.90E-06
RNU6-127	0.066205	0.246401	1.895987	0.000809	0.002099
AC069499	0.226473	0.946434	2.06316	2.06E-10	9.58E-09
TAS2R4	0.078442	0.227735	1.537655	2.80E-06	1.72E-05
AC092112	0.124174	1.157812	3.22097	6.29E-05	0.000234
TSLP	0.751837	0.191789	-1.9709	5.06E-07	4.14E-06
MIR4427	0.17586	1.029368	2.549259	5.39E-05	0.000206
SKAP1-AS	0.138243	0.403161	1.54415	0.000151	0.000495
AC131532	0.016865	0.489721	4.859817	0.00057	0.001547
AC015853	0.081826	0.284108	1.795806	5.44E-08	6.82E-07
SNORA80	0.211034	0.618595	1.551523	2.89E-05	0.000122
LBX2	0.232325	0.950405	2.032399	4.50E-09	9.38E-08
MATN3	0.323093	2.41404	2.90143	3.66E-08	4.96E-07
FGL2	65.06057	14.33433	-2.18231	0.000174	0.000558
EDN2	2.576088	0.701798	-1.87605	0.000391	0.001118
Z69720.2	0.090986	0.284614	1.645289	8.11E-05	0.000291
AGRN	11.79613	38.32951	1.700142	9.87E-11	6.05E-09
TCEAL2	11.44407	2.300866	-2.31435	3.25E-05	0.000135
AC245100	0.117394	0.373253	1.668794	4.70E-08	6.09E-07
NUDT19P!	0.071692	0.26199	1.869635	0.000903	0.002308
C2orf15	0.612502	1.755681	1.519243	1.80E-09	4.63E-08
SIX1	0.146921	0.841131	2.517288	9.98E-06	4.99E-05
ASB9P1	0.177961	0.509845	1.518498	2.35E-06	1.48E-05
GFRA1	6.38028	1.74128	-1.87347	3.00E-06	1.82E-05
C8orf31	0.430499	1.938184	2.170625	3.18E-07	2.84E-06

AC087277	0.042526	0.285298	2.746061	0.000588	0.001591
A2M-AS1	1.43037	0.497709	-1.52301	0.006391	0.01253
TERT	0.075316	0.503376	2.740602	8.08E-07	6.12E-06
SELENOO	0.040447	2.228605	5.783981	0.000204	0.000642
DNASE1L3	4.536189	0.998975	-2.18296	1.34E-06	9.32E-06
RNU6-133	0.072649	0.379502	2.38509	5.06E-05	0.000196
EVX1	0.023466	0.643071	4.776309	1.61E-07	1.65E-06
CKM	2.413892	0.447206	-2.43235	2.78E-05	0.000118
RF02133	0.047081	0.360315	2.936044	0.000279	0.000838
RIBC2	0.407185	1.291187	1.664944	0.000102	0.000354
USP2	3.522492	0.979743	-1.84612	1.36E-06	9.42E-06
RNF144A-	0.048139	0.222993	2.211706	1.03E-08	1.81E-07
AKR1C2	10.3114	3.338668	-1.6269	8.62E-08	9.90E-07
RNA5SP30	0.036735	0.245291	2.739277	0.00407	0.008479
TUBG1P	0.144203	0.435992	1.596205	2.16E-07	2.08E-06
CST4	0.019621	1.943703	6.630233	7.24E-12	2.00E-09
C17orf53	0.81497	2.528061	1.633212	4.66E-10	1.70E-08
ANKRD35	3.919009	0.818323	-2.25975	7.79E-07	5.93E-06
ASPG	1.036713	0.309083	-1.74595	0.007538	0.01445
UBE2Q1-A	0.105114	0.378174	1.847099	2.79E-07	2.55E-06
MUC21	190.9218	4.081185	-5.54785	0.005696	0.011348
AP000346	0.115872	0.37591	1.69785	8.50E-08	9.80E-07
COL7A1	1.204967	4.439776	1.881493	1.25E-08	2.10E-07
DNAH2	0.046481	0.326435	2.812068	1.22E-06	8.60E-06
RNA5SP24	0.076386	0.465707	2.608048	1.98E-07	1.95E-06
AC009407	0.321473	0.913446	1.506622	0.001013	0.002547
RNU6-840	0.111145	0.321123	1.530678	0.008393	0.015865
AC099811	0.077918	0.225582	1.533626	7.25E-06	3.82E-05
P4HA3	0.187521	0.980261	2.386117	5.06E-11	4.13E-09
AC004923	0.1996	0.715984	1.842813	2.79E-08	3.97E-07
NKAPL	0.706653	0.231432	-1.61041	0.000197	0.000622
KIF20B	1.782717	5.367274	1.590112	7.66E-11	5.26E-09
AC091887	0.075879	0.220775	1.540798	1.05E-05	5.20E-05
AL049539.	0.12858	0.513637	1.998079	2.16E-07	2.08E-06
AC008957	0.075384	0.256831	1.768499	0.000862	0.002219
SOSTDC1	14.59041	2.834807	-2.3637	5.20E-08	6.57E-07
RNU6-387	0.347359	1.391872	2.002529	1.89E-07	1.87E-06
OSM	0.580844	2.134351	1.877574	0.000137	0.000455
AACSP1	0.025141	0.299444	3.574147	0.002687	0.005915
AL136115.	0.197591	0.589364	1.576639	1.24E-06	8.69E-06
STON1	7.069824	2.219725	-1.67129	0.007171	0.013839
TOP2A	5.567495	35.16971	2.659233	9.90E-12	2.14E-09
RNASE3	0.041505	0.286695	2.788156	0.009209	0.017187
AL161431.	0.36487	3.336752	3.192989	1.17E-06	8.32E-06
AC105036	0.072847	0.250099	1.779561	8.76E-06	4.47E-05
AKAP6	1.924268	0.549185	-1.80895	0.002542	0.005639
HELB	0.510023	1.487523	1.544279	9.57E-11	5.99E-09
RNA5SP43	0.085219	0.318025	1.899904	0.000485	0.001345
MIR3690	0.072369	0.369854	2.353514	0.00294	0.006393
IGHV3-75	1.65634	0.189654	-3.12656	0.004629	0.009483
TBILA	0.272956	0.941587	1.786428	1.40E-06	9.64E-06
MYL9	961.4313	212.3496	-2.17874	1.76E-05	8.00E-05
AL591468.	0.075167	0.23418	1.639443	0.000177	0.000565
BUB1B	1.597979	6.355347	1.991722	2.94E-10	1.22E-08
PLK1	4.683686	13.83323	1.562422	1.17E-08	1.99E-07
CCKAR	2.576081	0.586849	-2.13412	6.82E-06	3.63E-05
AC007342	0.31847	1.305648	2.035535	1.31E-05	6.27E-05
AC116025	0.122171	0.690526	2.498794	2.36E-08	3.48E-07

AC083809	0.148902	2.337504	3.972538	0.004825	0.009833
MIR1203	0.075163	0.412603	2.456663	0.000397	0.001135
C2orf48	0.093871	0.582978	2.63469	1.73E-08	2.71E-07
SPDYC	0.154184	1.497717	3.280041	1.11E-07	1.22E-06
TRIM59	0.500838	1.49608	1.578771	1.35E-10	7.40E-09
HSPA8P3	0.067344	0.279881	2.055198	4.77E-07	3.94E-06
AL590006.	0.183089	0.527491	1.526601	3.05E-05	0.000128
AC015660	0.126739	0.362949	1.517902	0.000999	0.002519
CDKN2A	1.469378	8.261832	2.491256	0.00059	0.001592
CCDC69	30.11264	9.469735	-1.66897	6.52E-08	7.88E-07
Z99127.2	0.099231	0.42821	2.109458	3.18E-07	2.84E-06
RNA5SP32	0.07633	0.430657	2.496207	6.36E-06	3.43E-05
MSH5	0.251189	1.099135	2.129525	2.56E-10	1.11E-08
PCSK9	2.044024	6.272936	1.617729	0.000134	0.000448
Z98257.1	0.049849	0.454267	3.187899	5.64E-05	0.000214
FOXD2	0.711843	2.173667	1.6105	1.56E-08	2.49E-07
ZNF367	1.583941	4.714525	1.573594	1.42E-10	7.66E-09
MMP2-AS	0.059848	0.259839	2.118239	5.17E-06	2.88E-05
LGR5	0.794661	6.517745	3.035961	5.12E-05	0.000198
CKAP2L	0.857177	3.232917	1.915171	2.37E-10	1.05E-08
DDX12P	0.399257	1.388833	1.798483	1.02E-09	3.03E-08
PPP1R1B	12.87048	53.27717	2.049452	0.011291	0.020458
AC024451	0.105944	0.398797	1.912351	8.06E-08	9.37E-07
AC009704	0.130326	0.405986	1.639306	3.54E-07	3.09E-06
AC022022	0.061153	0.218362	1.836224	4.02E-05	0.000162
NPIPB11	0.166891	0.498443	1.57852	1.65E-08	2.61E-07
NCAPG2	1.706408	5.058026	1.567612	3.60E-10	1.40E-08
MKI67	4.882431	20.59101	2.076343	1.12E-10	6.54E-09
SRCIN1	0.331097	1.493205	2.173085	0.000101	0.000351
AC020661	0.102442	0.329202	1.684164	1.28E-05	6.16E-05
UTS2	0.072096	0.549633	2.930478	0.000286	0.000856
AC079336	0.130793	0.384544	1.555861	3.74E-05	0.000152
CFAP43	0.070632	0.238713	1.756883	2.59E-07	2.40E-06
MIR1249	0.085112	0.406294	2.255095	5.72E-06	3.15E-05
AL357134.	0.080551	0.251644	1.643416	0.002327	0.005227
MIR4525	0.01502	0.512778	5.093331	1.54E-07	1.58E-06
HOXA11	0.225183	2.067289	3.198571	6.38E-09	1.24E-07
FOXF2	28.14413	9.22843	-1.60868	0.001194	0.002939
AL117187.	0.18931	0.550493	1.539973	0.000113	0.000386
AC067930	0.160286	0.568741	1.827122	3.41E-06	2.02E-05
CAPN12	0.379113	1.119681	1.562387	0.000233	0.000719
AC009093	0.078152	0.280753	1.844949	1.56E-08	2.49E-07
DOC2A	0.057625	0.270901	2.232988	0.000121	0.00041
RNF223	6.640977	2.180475	-1.60675	0.011602	0.020945
RNU7-19F	0.074176	0.327902	2.144236	0.016799	0.028887
AL031009.	0.088247	0.32526	1.881981	1.32E-09	3.67E-08
TRMT2B-/	0.122419	0.409506	1.74206	1.37E-05	6.47E-05
DLGAP5	1.956386	6.920068	1.822595	1.09E-09	3.16E-08
AL391994.	0.126802	0.41253	1.701924	1.48E-05	6.95E-05
AL353804.	0.12435	0.376009	1.596356	7.70E-06	4.02E-05
LINC0257!	0.68473	3.158531	2.205646	0.000318	0.000939
ARHGAP8	0.216595	0.922575	2.090669	6.13E-11	4.53E-09
CWH43	3.688168	0.219911	-4.06791	8.11E-08	9.42E-07
AC105460	0.708585	13.90422	4.294437	0.002709	0.005958
SGO1	0.685339	3.116179	2.184889	8.25E-12	2.04E-09
AC007991	0.490635	4.331672	3.142203	2.39E-05	0.000104
PPIAP59	0.070224	0.214426	1.610444	3.60E-06	2.12E-05
SPAG5	1.9925	7.149978	1.843359	5.19E-10	1.83E-08

PNOC	1.999265	0.603903	-1.72708	0.000154	0.000503
ENPP7P8	0.113228	0.55409	2.290884	0.000144	0.000476
AC012073	0.366883	1.450418	1.983078	1.23E-11	2.34E-09
NACAD	2.448082	0.830199	-1.56012	0.00095	0.002411
PCOLCE2	3.279561	0.555307	-2.56215	1.88E-07	1.86E-06
IL36G	0.090636	0.273346	1.592579	0.006619	0.012919
LINC0224	0.124718	0.425912	1.771889	0.000481	0.001338
PRPH	0.77063	0.176836	-2.12363	0.000107	0.000369
AC013410	0.111692	0.324188	1.537308	0.000703	0.001855
AP000695	0.145444	0.669017	2.201582	6.32E-10	2.13E-08
AP000943	0.008941	0.21824	4.609389	6.14E-07	4.85E-06
TMEM132	0.008341	1.095391	7.037014	0.001858	0.004305
TNS4	7.789098	33.50686	2.104929	0.00386	0.008107
RPL12P25	0.105696	0.308521	1.545443	5.39E-05	0.000206
PTGES2-A	0.042278	0.338273	3.000212	1.26E-07	1.35E-06
IQANK1	4.454378	13.30689	1.578878	2.28E-07	2.16E-06
MIR770	0.524818	0.178346	-1.55714	0.013958	0.024598
BOLA2P3	0.054968	0.214836	1.966579	7.65E-05	0.000277
CADM3	5.800038	0.923949	-2.65018	6.22E-10	2.10E-08
GFRA2	0.951112	0.214433	-2.14909	1.66E-07	1.68E-06
AC010980	0.632415	0.174311	-1.85921	0.008035	0.015271
HSPE1P7	0.119253	0.410905	1.78478	1.06E-06	7.71E-06
ABALON	0.316826	1.097697	1.792717	3.78E-09	8.23E-08
BTG2	263.7523	54.75558	-2.26811	0.000295	0.000877
AC020661	0.147648	0.426423	1.530127	9.67E-06	4.87E-05
FAM72C	0.047651	0.326579	2.776855	6.81E-10	2.26E-08
IGFL4	0.028781	0.305719	3.409039	4.52E-05	0.000178
ASAH2	3.326968	0.301141	-3.4657	0.013455	0.023824
AL121839.	0.366856	1.062922	1.53475	5.94E-07	4.72E-06
IGLJ1	3.150749	0.808226	-1.96286	0.015851	0.027483
RNA5SP47	0.099146	0.374086	1.915742	0.013617	0.024094
HSPA6	1.019355	3.486177	1.773989	0.001954	0.004493
SCN7A	3.209701	0.545744	-2.55614	5.25E-07	4.28E-06
HS3ST6	3.42985	0.978551	-1.80943	3.84E-07	3.30E-06
MIR4326	0.088538	0.574862	2.698846	2.34E-05	0.000102
SNORA15	0.132207	0.444127	1.748172	0.00083	0.002146
AC098934	0.505242	1.718705	1.766275	9.82E-07	7.22E-06
AC007014	0.072426	0.30573	2.077668	4.44E-08	5.82E-07
RABGAP1I	0.21214	0.688551	1.698546	0.000173	0.000556
MIR326	0.102327	0.322667	1.656859	0.00798	0.015184
DNAH5	0.059854	0.308006	2.363441	1.83E-06	1.21E-05
TPM3P6	0.262868	0.782515	1.573779	2.09E-06	1.34E-05
CCL21	126.7683	38.40042	-1.723	1.15E-06	8.21E-06
NPSR1	0.877906	2.996746	1.771258	0.000115	0.000392
HIST1H2A	0.092329	0.592646	2.682312	0.000737	0.001936
AC099518	0.111133	0.399514	1.845956	2.67E-06	1.65E-05
AC244093	0.103599	0.300118	1.534518	1.27E-05	6.12E-05
PGA5	188.6147	16.13078	-3.54755	4.13E-05	0.000165
TMEM132	0.716031	0.18878	-1.92331	3.27E-09	7.32E-08
RPS20P33	0.297231	0.916272	1.62419	2.24E-08	3.34E-07
AC039056	0.135769	0.389377	1.520009	3.22E-06	1.92E-05
FEZF1-AS	0.183193	1.770998	3.273124	1.54E-09	4.13E-08
RNASE1	461.0775	126.7086	-1.86349	7.15E-05	0.000261
HMGB1P6	2.38614	8.851525	1.891248	9.98E-09	1.78E-07
SNORD88	0.179293	0.796361	2.151106	1.72E-06	1.14E-05
DEFB4A	5.460552	1.838637	-1.57041	0.009652	0.017885
AC080080	0.08058	0.280125	1.797577	4.63E-09	9.61E-08
ART3	0.86259	0.248287	-1.79667	0.011138	0.020228

GSTA1	148.0598	25.4761	-2.53896	0.011841	0.021302
KIAA1549	0.595713	2.103859	1.820348	7.63E-09	1.43E-07
AC018607	0.142608	0.538163	1.915986	2.76E-07	2.53E-06
GCNT7	0.081334	0.244358	1.587066	5.27E-08	6.64E-07
TAGLN3	0.866699	0.22623	-1.93774	0.000306	0.000907
HMGN2P1	0.06347	0.275269	2.1167	5.48E-07	4.42E-06
LINC0035!	0.010068	0.402885	5.322469	1.97E-05	8.79E-05
MIR548K	0.091978	0.360395	1.970213	0.001677	0.003933
MUC16	0.027321	0.507176	4.214386	9.04E-05	0.000319
FCGR3A	4.877209	17.61283	1.852499	1.34E-08	2.22E-07
LINC0134:	0.120867	0.4323	1.838616	1.36E-07	1.43E-06
LINC0259!	0.554846	1.784438	1.68531	9.70E-07	7.14E-06
RPS14P4	0.122576	0.72691	2.568095	9.57E-09	1.72E-07
AC096720	0.192392	0.571343	1.570308	9.15E-06	4.64E-05
LINC0241{	0.0211	0.527185	4.643003	0.000137	0.000456
PPP1R3C	14.60264	2.964743	-2.30025	2.01E-06	1.30E-05
HDC	1.390266	0.456051	-1.60809	1.61E-05	7.44E-05
AP000695	0.220443	0.73665	1.74057	2.37E-07	2.23E-06
RN7SL200	0.189083	0.60337	1.674023	4.03E-06	2.34E-05
INHBB	1.612886	6.107123	1.920849	0.007379	0.014189
CHIA	6.934228	2.227163	-1.63853	0.001371	0.003314
AC109992	0.067318	0.251643	1.902304	1.07E-07	1.19E-06
MIR3155A	0.101952	0.522836	2.358463	9.24E-05	0.000325
LOX	2.654112	8.319062	1.648192	4.13E-08	5.48E-07
CHRDL1	13.24683	3.673576	-1.85039	1.98E-07	1.95E-06
ADHFE1	1.917183	0.490638	-1.96626	5.58E-09	1.12E-07
AC080129	0.212762	0.884056	2.0549	0.000324	0.000952
AC114488	0.112889	0.329126	1.543734	0.000332	0.000972
PNPLA3	0.124565	0.524248	2.073355	0.005565	0.011121
MIR5695	0.201986	0.621196	1.620794	0.030025	0.047767
AIF1L	10.9444	2.360682	-2.21292	0.001506	0.003592
LINC0155{	0.132403	0.599206	2.178121	0.000112	0.000382
TROAP	1.709073	6.799173	1.992145	3.18E-10	1.29E-08
AL450992.	1.43079	4.104096	1.520252	0.013276	0.023545
AC011479	0.085949	0.348022	2.017619	2.88E-08	4.08E-07
FIBCD1	0.227263	2.515665	3.468502	0.000622	0.001667
Z99127.1	0.114482	0.381912	1.738117	2.56E-06	1.60E-05
POU5F1B	0.045143	0.273993	2.601562	8.64E-06	4.42E-05
AC007099	0.008304	0.40859	5.620694	3.23E-05	0.000134
IGLV7-35	1.321461	0.122565	-3.43052	0.014887	0.026015
MIR559	0.544009	2.048734	1.913032	7.50E-07	5.76E-06
RN7SKP2{	0.10831	0.351903	1.700004	1.97E-05	8.79E-05
IGKV2-18	1.804282	0.223471	-3.01326	0.008381	0.015849
C1QTNF2	2.313618	0.765595	-1.5955	0.000132	0.00044
MIR3189	0.223571	2.966157	3.729788	1.28E-09	3.60E-08
SNORD11	0.073203	0.36239	2.307569	0.000438	0.001233
SOX4	7.884534	37.08781	2.233848	8.56E-13	1.31E-09
AC026369	0.130065	0.776578	2.577904	8.81E-12	2.06E-09
SNX25P1	0.180082	0.657278	1.86785	3.61E-08	4.92E-07
DCUN1D2	0.174181	0.523289	1.587022	1.99E-06	1.29E-05
ZNF761	1.515271	4.82078	1.669691	1.11E-11	2.25E-09
AC026356	0.360408	1.149949	1.673869	6.13E-11	4.53E-09
AL445250.	0.066641	0.2782	2.061652	0.00818	0.015524
AL353693.	0.026652	0.314343	3.559998	2.04E-05	9.08E-05
AL136115.	0.138858	0.576743	2.054313	2.15E-08	3.23E-07
AC006330	0.134806	0.455944	1.757974	3.03E-08	4.27E-07
MYO1B	3.037639	8.738932	1.524507	4.17E-11	3.74E-09
FLJ22447	0.081158	0.351799	2.115953	0.000108	0.000371

ACTG2	876.7199	254.6278	-1.78373	2.40E-05	0.000104
AL034555.	0.073058	0.278898	1.932619	8.85E-05	0.000313
HOXA9	0.097298	1.632638	4.068648	5.82E-08	7.20E-07
KCNQ1OT	0.133301	0.530466	1.992568	8.48E-07	6.37E-06
ORC6	0.628686	2.385653	1.923973	3.28E-10	1.31E-08
RPL31P58	0.128191	0.419819	1.711469	8.38E-06	4.32E-05
CHST1	0.708756	2.152023	1.602333	4.56E-09	9.49E-08
AL137244.	0.077009	0.288224	1.90408	6.47E-07	5.08E-06
BCAR4	0.017642	0.570189	5.014335	0.0022	0.004985
MIR3131	1.585671	5.581353	1.815522	0.005495	0.011007
AL513327.	0.072299	0.243731	1.75325	9.63E-06	4.85E-05
MIR320B2	0.160091	0.62442	1.96362	3.47E-06	2.05E-05
RNU6-11	0.108095	0.311955	1.529041	0.010152	0.018689
AC011298	0.070532	0.347129	2.299121	0.019354	0.03263
BMP3	3.70352	1.013422	-1.86966	0.007751	0.014806
AC104958	0.712293	2.782324	1.965749	0.012248	0.02192
AC011676	0.080252	0.402913	2.327866	3.81E-09	8.28E-08
FAM183A	0.164079	0.477472	1.541025	0.021893	0.036363
AP002449	0.189364	0.619846	1.710746	1.61E-07	1.65E-06
AC073335	1.263181	3.986079	1.65791	1.48E-08	2.39E-07
AQP10	6.727235	0.283113	-4.57057	0.000213	0.000664
TRIM50	3.352649	0.394307	-3.08791	0.000272	0.000821
NKD2	0.627885	3.979653	2.664071	0.000223	0.00069
SUCNR1	0.718946	2.337185	1.700817	1.22E-06	8.60E-06
AC012467	0.080694	0.622782	2.948191	1.45E-07	1.50E-06
AC005324	0.07311	0.266452	1.865742	2.21E-07	2.11E-06
AC093001	0.161012	2.591255	4.00841	0.004729	0.009662
AC141557	0.098144	0.602794	2.618696	1.82E-08	2.81E-07
AC108676	0.049586	0.214594	2.113602	0.001087	0.00271
SLC6A4	1.441096	0.18069	-2.99558	0.000317	0.000935
CLDN6	0.05396	9.68277	7.487383	0.000251	0.000767
VSIG2	155.2795	35.36748	-2.13437	0.014766	0.025828
AC011290	0.271697	0.782243	1.525618	1.04E-06	7.60E-06
AP003467	0.075406	0.248933	1.72301	3.59E-05	0.000147
AC027237	0.089506	0.275258	1.620735	4.96E-10	1.79E-08
AL031709.	0.092573	0.264034	1.512066	2.26E-05	9.91E-05
AC129492	0.109386	0.408573	1.901166	0.000374	0.001075
AVIL	0.497498	1.613761	1.697665	0.000611	0.001642
MIR4802	0.227412	0.692196	1.60587	0.001426	0.00343
DAAM2	9.98563	3.509296	-1.50867	0.000205	0.000642
GRB7	5.844573	32.3588	2.468989	3.99E-07	3.40E-06
LAMC2	5.687372	35.41603	2.638568	2.26E-10	1.03E-08
LINC0159	0.0512	0.497055	3.279179	3.66E-05	0.00015
NPFFR1	0.056695	0.281089	2.309725	7.69E-07	5.88E-06
TFRC	9.810024	30.19539	1.622	2.50E-08	3.63E-07
AP002992	0.041815	0.285253	2.770132	0.020401	0.034203
TMPRSS5	0.132934	0.852051	2.680226	0.000111	0.000379
AUNIP	0.531548	1.914363	1.848591	1.56E-10	8.04E-09
ASGR1	0.252844	0.858547	1.763648	0.012925	0.022996
PM20D1	0.658038	0.223937	-1.55508	0.011291	0.020458
CKAP2	3.544858	12.1172	1.773256	1.16E-10	6.61E-09
ADAMTS1	0.022209	0.317189	3.836131	5.99E-10	2.04E-08
CGAS	1.633319	4.670196	1.515676	5.60E-10	1.94E-08
BIRC7	0.049717	1.066605	4.423129	6.27E-10	2.11E-08
AC092171	0.177404	0.606973	1.774593	3.61E-08	4.92E-07
TWNK	1.742737	4.935	1.501695	2.11E-11	2.71E-09
SFRP4	5.442466	28.3795	2.382517	3.33E-06	1.98E-05
AL138689.	0.148101	0.469262	1.663809	0.000121	0.00041

RN7SL535	0.109317	0.391079	1.838936	1.29E-06	8.99E-06
AC008109	0.003282	0.460544	7.132839	0.000111	0.000381
NOX4	0.210662	0.623433	1.565304	4.73E-08	6.11E-07
AL928654.	0.39677	1.244601	1.649309	1.19E-09	3.38E-08
LHFPL3	0.15326	0.857703	2.484501	0.007555	0.014482
KNL1	0.937057	3.183377	1.764349	3.23E-09	7.25E-08
ANKRD61	0.165242	0.584785	1.823326	1.56E-10	8.04E-09
NEURL3	0.408132	1.857686	2.186398	2.95E-05	0.000124
RNA5SP1:	0.654997	1.969753	1.588455	0.019636	0.033082
RNU6-759	0.403303	1.141952	1.501566	5.16E-05	0.000199
COL5A1	12.83991	38.63024	1.589095	9.02E-07	6.71E-06
CDH19	2.119863	0.365243	-2.53704	6.32E-07	4.97E-06
KLF8	1.626891	0.493595	-1.72072	0.000926	0.00236
PPM1H	1.37828	6.132763	2.153668	3.98E-11	3.62E-09
SCARNA8	0.111557	0.329895	1.564221	0.000435	0.001227
AC011462	0.239634	0.721315	1.589794	2.24E-07	2.14E-06
CDCA8	4.725649	13.53421	1.518026	7.74E-09	1.45E-07
TAC1	2.42615	0.673154	-1.84966	0.00221	0.005001
RF02140	0.034345	0.420611	3.614324	1.70E-06	1.13E-05
GTSE1	1.403877	5.236865	1.899286	5.03E-10	1.80E-08
HSPD1P6	0.058708	0.212594	1.856462	9.12E-11	5.85E-09
CYCSP34	0.212656	0.937311	2.140004	1.62E-08	2.56E-07
GDF7	0.544006	0.179531	-1.59939	1.96E-05	8.75E-05
AC011287	0.026954	0.476759	4.144703	0.000368	0.00106
KLK6	3.717638	31.46242	3.081171	2.67E-05	0.000114
LUCAT1	0.080467	0.360884	2.165071	7.35E-06	3.86E-05
AP005131	0.096982	0.277269	1.5155	0.001026	0.002575
AL606970.	0.056771	0.23438	2.045614	0.010357	0.019024
LRP8	0.4595	2.255559	2.295349	6.23E-11	4.54E-09
AC122108	0.047575	0.265757	2.481844	0.000446	0.001252
AC008121	0.072615	0.224558	1.628744	0.000565	0.001536
LMOD1	110.7755	26.66531	-2.0546	2.90E-06	1.77E-05
MELTF	0.75786	5.487575	2.856165	2.48E-10	1.09E-08
DUXAP10	0.018732	0.267166	3.83413	1.06E-10	6.36E-09
F5	1.358786	8.164867	2.587111	5.28E-05	0.000203
LINC0165!	0.090039	0.454788	2.336565	1.83E-05	8.26E-05
RNU6-78(	0.306157	0.898304	1.552932	3.91E-05	0.000158
AP001429	0.108594	0.363894	1.744571	3.51E-08	4.81E-07
MIR4641	0.136636	0.6885	2.333122	7.99E-05	0.000287
TRAJ5	0.094706	0.383594	2.018055	0.003422	0.007301
AC078883	0.075171	0.329755	2.133148	1.17E-05	5.71E-05
GPER1	4.705367	1.314855	-1.8394	1.03E-05	5.13E-05
INAFM2	46.01016	12.78135	-1.84791	0.021338	0.035558
LCN1	0.003408	0.569381	7.384423	4.37E-07	3.67E-06
PLA2G7	2.511976	7.975337	1.666723	9.70E-08	1.09E-06
MSLN	8.353992	50.11396	2.584675	7.56E-06	3.96E-05
CCL18	10.06574	29.0937	1.531253	0.002134	0.004851
MTATP6P:	0.060914	0.263511	2.113026	8.67E-07	6.49E-06
MAL2-AS:	0.096458	0.400296	2.053102	3.71E-08	5.02E-07
MIR3176	0.949863	3.711577	1.966241	5.23E-08	6.61E-07
BRCC3P1	0.103627	0.314258	1.600556	1.18E-08	2.01E-07
MUC5B	1.860292	15.20848	3.031276	0.015874	0.027505
MESP2	0.138195	0.575758	2.05876	2.62E-05	0.000112
AC132938	0.142346	0.415969	1.54707	5.41E-08	6.79E-07
NOP56P3	0.089704	0.390483	2.12201	6.59E-08	7.95E-07
MIR3192	0.149879	0.543894	1.859531	2.97E-05	0.000125
GAL3ST2	0.094929	2.389512	4.653725	1.46E-08	2.36E-07
PRR19	0.218303	0.925423	2.083779	1.06E-08	1.84E-07

NRG2	0.877363	0.272125	-1.6889	5.51E-06	3.05E-05
AC124798	0.58697	2.422081	2.044889	6.61E-08	7.96E-07
AL117329.	0.00674	0.305943	5.504355	2.85E-06	1.74E-05
IGKV7-3	3.415459	0.650095	-2.39336	0.017006	0.02918
WISP2	2.845376	0.413387	-2.78305	2.45E-12	1.40E-09
DNAAF3	0.160598	0.552846	1.78342	4.69E-07	3.89E-06
AC103563	1.794437	0.148398	-3.59598	4.32E-12	1.58E-09
AADACL2-	0.075044	0.220042	1.551976	0.003973	0.008311
PCP4L1	8.471043	1.486724	-2.5104	5.62E-05	0.000213
AL121782.	0.050329	0.241918	2.265064	1.12E-06	8.05E-06
TESMIN	0.339138	1.608603	2.245862	2.03E-10	9.51E-09
AC020907	0.255861	0.784847	1.617049	0.005857	0.011617
FRRS1L	1.644469	0.458571	-1.84241	7.60E-05	0.000275
AL356234.	0.066366	0.381255	2.522234	1.81E-05	8.17E-05
GUCY1B2	0.034263	0.314142	3.196697	3.42E-09	7.54E-08
NIPAL4	2.239323	0.525103	-2.09239	0.002367	0.005301
SAA2	1.107449	4.378332	1.983141	0.011721	0.021132
RNU6-379	0.181466	0.527129	1.538459	0.000352	0.001021
AC087465	0.061651	0.214263	1.797181	0.000585	0.001584
BNIP3P11	0.18675	0.975307	2.384748	7.31E-11	5.12E-09
NKX2-5	0.091511	0.769094	3.07114	0.019072	0.03223
MELK	2.414598	9.376897	1.957327	2.26E-10	1.03E-08
AC008379	0.11246	0.353657	1.652939	3.84E-06	2.25E-05
AP001207	0.070509	0.216757	1.620189	0.000379	0.00109
ITIH2	0.07061	1.602725	4.504519	0.000121	0.000408
APOE	45.807	144.7933	1.660355	2.80E-06	1.72E-05
TMEM238	24.48782	6.367398	-1.94329	0.00931	0.017345
MYOM1	4.052298	1.258008	-1.6876	0.002117	0.004816
SNORA51	0.084945	0.283377	1.738119	0.000432	0.001219
MIR548XH	0.011854	0.252522	4.41298	0.011788	0.021231
AC010435	0.106736	0.308628	1.53182	5.04E-06	2.82E-05
AC079305	0.56112	0.182291	-1.62207	0.000155	0.000506
NETO2	0.420318	1.728296	2.039798	8.38E-07	6.31E-06
AC012531	0.013215	0.308519	4.545102	3.91E-11	3.62E-09
AC011472	2.09658	0.661942	-1.66326	0.001457	0.003492
CTCFL	0.009976	0.35139	5.138412	0.01793	0.030561
ENHO	1.669295	0.560824	-1.57362	9.08E-05	0.00032
AGT	5.159137	26.56899	2.364542	3.45E-06	2.04E-05
AC022784	0.265085	3.012443	3.506409	8.08E-06	4.18E-05
SOX9	18.91488	70.06903	1.889255	4.90E-11	4.06E-09
Z99127.3	0.103679	0.364038	1.81197	0.000119	0.000403
AC006270	0.07561	0.24229	1.680077	0.001093	0.002724
AC010980	0.978911	0.236371	-2.05012	0.028015	0.045015
CMA1	1.758077	0.398143	-2.14264	1.40E-08	2.29E-07
AC127024	0.149008	0.471226	1.661027	1.79E-05	8.10E-05
LINC0258.	0.009253	1.26375	7.093539	0.000266	0.000805
HOTAIR	0.052907	1.303835	4.623161	1.15E-10	6.61E-09
MIR4755	0.281239	0.840338	1.579175	0.000442	0.001244
RF02250	0.030552	0.304225	3.315783	5.78E-05	0.000219
SNORD10	0.967446	3.334291	1.785126	4.14E-07	3.51E-06
KLHL35	0.251227	1.010952	2.008652	5.25E-07	4.28E-06
BLACAT1	0.180327	1.677623	3.217728	3.88E-10	1.48E-08
AC138150	0.111355	0.420813	1.918016	3.57E-05	0.000146
POLE2	1.052789	2.991283	1.506549	5.35E-10	1.87E-08
FADS6	1.38027	0.372554	-1.88943	0.002017	0.00462
HOTTIP	0.128856	1.267283	3.297911	3.58E-08	4.89E-07
FAM96AP.	0.145848	0.563381	1.949643	0.000168	0.000539
AC004383	0.103962	0.320533	1.624414	2.58E-05	0.000111

BNIP3P17	0.165606	0.790403	2.254836	6.27E-05	0.000234
PDILT	1.43091	0.197224	-2.85903	0.000304	0.000903
DWORF	0.056175	0.250611	2.157461	0.011382	0.02061
NAP1L2	1.688896	0.469973	-1.84543	2.23E-07	2.13E-06
CT83	0.303787	7.593888	4.643705	0.000164	0.000529
PI15	0.474555	1.805573	1.927811	0.027859	0.044774
FOXL1	0.536759	1.72039	1.680389	3.79E-07	3.27E-06
PLA1A	1.367908	4.106777	1.586036	7.15E-05	0.000261
ZNF29P	0.112605	0.360977	1.680633	0.000125	0.000421
STX8P1	0.035937	0.22587	2.651931	2.50E-05	0.000108
TRAJ37	0.082524	0.427492	2.373016	0.001591	0.003764
TM4SF1-A	0.171114	0.566577	1.727319	0.000555	0.001511
AC004898	0.094085	0.308348	1.71252	4.92E-08	6.29E-07
AC021945	0.0459	0.218523	2.25122	2.79E-08	3.97E-07
ZNF252P-	0.067381	0.223285	1.728476	1.25E-08	2.10E-07
EDN3	8.277222	2.255778	-1.87552	0.00085	0.002191
MIR5587	0.797668	3.37581	2.081373	8.53E-07	6.40E-06
BCAT1	0.852832	3.186749	1.901752	4.97E-05	0.000193
CBR1	50.66447	15.45918	-1.71251	1.65E-06	1.10E-05
TACR2	24.61692	7.207966	-1.77199	0.00404	0.008427
RNU6-748	0.053352	0.306157	2.520661	0.000234	0.000721
CDT1	3.790668	11.12253	1.552961	6.70E-08	8.05E-07
MIR6740	0.019774	0.473124	4.580557	1.41E-08	2.30E-07
RAET1K	0.03839	0.221341	2.527479	1.74E-08	2.73E-07
LINC0225	0.069602	0.25125	1.851933	1.44E-06	9.83E-06
AC105219	0.17351	0.507063	1.547146	5.80E-07	4.62E-06
ACBD7	0.183681	1.120213	2.608502	2.00E-08	3.05E-07
GDF15	7.563278	41.78331	2.465843	1.24E-09	3.50E-08
GPR84	0.264981	0.83929	1.663278	8.38E-07	6.31E-06
LRRC4	1.913333	0.671488	-1.51065	0.0001	0.000348
RNU6-343	0.198887	0.581136	1.546923	0.000701	0.001852
RNU6-115	0.047813	0.224783	2.233064	0.000943	0.002396
ATP5PFP1	0.788842	0.249989	-1.65787	0.023143	0.038157
RNU6-879	0.171026	0.586016	1.776722	0.000329	0.000964
SLC5A5	26.2553	3.205189	-3.03413	0.002749	0.006036
AKR1B15	2.829153	0.308149	-3.19867	9.23E-05	0.000325
AC245884	0.690803	2.041273	1.563123	1.75E-06	1.16E-05
COL4A5	8.036996	2.451404	-1.71305	0.000477	0.001326
PIP	0.021692	0.388138	4.161351	0.006425	0.012594
GLIS3	0.61235	1.741674	1.508047	7.01E-05	0.000257
HSPE1P6	0.223676	0.77963	1.801378	4.57E-07	3.81E-06
COL21A1	1.399754	0.372406	-1.91022	0.010048	0.018521
AL157838.	0.188313	0.709803	1.914283	9.21E-10	2.83E-08
AC048344	0.186872	0.569918	1.608706	5.65E-07	4.53E-06
TREML3P	0.070691	0.226844	1.682105	0.003302	0.00707
AC025280	1.280363	0.398417	-1.6842	0.004367	0.009013
LGALS9B	5.566091	1.128465	-2.3023	0.000357	0.001033
ZNF724	0.341497	1.079481	1.660393	1.07E-06	7.75E-06
RYR3	0.728513	0.218351	-1.7383	0.001518	0.003616
CNN1	434.9242	111.879	-1.95883	7.73E-06	4.03E-05
SNORD11	0.101732	0.457623	2.169386	3.07E-05	0.000128
MIR548L	0.161921	0.529347	1.708922	0.000312	0.000922
MIR4677	0.13454	0.47253	1.812368	0.000302	0.000896
CYP4B1	3.243323	0.540634	-2.58475	5.06E-07	4.14E-06
SPEG	5.708746	1.703384	-1.74477	0.002443	0.005447
MMP3	0.630901	19.14074	4.923089	1.53E-06	1.04E-05
PPP1R26-.	0.190181	0.609622	1.680542	2.33E-10	1.04E-08
TMEM220	1.362647	0.451395	-1.59395	6.97E-08	8.31E-07

HBA2	47.62543	15.37647	-1.63101	6.35E-08	7.74E-07
SGK494	0.306664	0.886035	1.530703	4.01E-09	8.63E-08
AC004080	0.167169	1.221801	2.869634	3.01E-07	2.71E-06
NPY	2.723065	0.921326	-1.56345	3.07E-05	0.000128
EPHA7	7.114151	1.301708	-2.45029	0.008035	0.015271
CARMN	3.546036	1.151216	-1.62305	0.016295	0.028133
LINC0234(	0.096546	0.429267	2.152584	0.000432	0.001219
AL359922.	0.092782	0.269618	1.538993	6.97E-07	5.41E-06
FAM107A	6.354434	1.598574	-1.99098	2.83E-09	6.55E-08
AP001372	0.04815	0.240033	2.31761	6.84E-08	8.19E-07
CYP2AB1F	0.639279	0.181616	-1.81555	0.001577	0.003737
DNMT3B	0.403726	2.638932	2.708506	5.31E-11	4.26E-09
KCNH8	0.156912	0.667148	2.088048	0.000858	0.002207
STRA6	0.100348	2.727799	4.764652	5.08E-12	1.80E-09
AC004593	0.075907	0.307034	2.016089	2.12E-05	9.37E-05
SMKR1	0.369754	1.317299	1.832945	8.26E-06	4.27E-05
AC079949	0.175521	0.90642	2.36854	0.007019	0.013583
LINC0198(	0.009857	0.909739	6.528158	1.91E-05	8.60E-05
CCDC198	0.030196	0.844507	4.805695	2.11E-05	9.34E-05
HSD3BP5	0.101975	0.539178	2.402546	0.010614	0.019397
CSAG2	0.006284	0.623203	6.631949	4.22E-06	2.43E-05
RNU7-123	0.165265	0.568603	1.782637	0.000331	0.00097
AC007285	0.079914	0.263057	1.718847	6.26E-07	4.93E-06
RBM12B-/	0.436474	1.261565	1.531248	2.34E-09	5.67E-08
UBE2C	13.47531	61.37355	2.187299	7.69E-10	2.48E-08
FOXCUT	0.035843	0.220398	2.620329	0.000154	0.000503
AP000424	0.04089	0.219508	2.424458	0.000315	0.000929
MAT1A	0.063195	0.755364	3.579278	0.008651	0.016281
LCN12	0.184019	1.040303	2.499079	4.63E-05	0.000182
AC002398	1.68002	0.401984	-2.06327	0.000157	0.00051
LRRC10B	3.07889	0.96884	-1.66808	0.027859	0.044774
AC099518	0.095869	0.319078	1.734774	2.35E-08	3.47E-07
MXRA5	9.242679	30.37779	1.716634	1.25E-08	2.10E-07
TRAJ2	0.193557	0.762493	1.977968	0.000927	0.002362
CLPSL1	0.050274	0.421986	3.069313	0.004075	0.008488
HOXC12	0.011557	2.189306	7.56555	1.20E-05	5.84E-05
KIF2C	2.755568	9.292789	1.753762	8.04E-10	2.55E-08
MMP11	2.204532	21.35944	3.276329	6.86E-12	1.99E-09
RNU7-70F	0.093349	0.322545	1.788799	0.023886	0.039217
PLPP4	0.381118	1.130896	1.569159	0.01341	0.023762
C12orf75	13.16611	38.55914	1.550243	2.53E-07	2.35E-06
AC090181	0.493205	1.571392	1.671783	2.17E-07	2.08E-06
AC099850	2.51814	9.278015	1.881458	7.31E-11	5.12E-09
RNU6-79F	0.053273	0.250769	2.23489	0.001138	0.002821
AL355796.	0.02604	0.256424	3.299704	4.06E-05	0.000163
RNU6-501	0.152447	0.449312	1.559409	0.012246	0.02192
PRUNE2	20.60168	7.072717	-1.54243	0.001703	0.003984
MIR3117	0.114827	0.337317	1.554639	0.013167	0.023381
FOXH1	0.0483	0.705885	3.869336	1.21E-08	2.06E-07
AL583810.	0.144735	0.483132	1.739	6.64E-07	5.18E-06
ACAN	0.115143	1.274846	3.468819	1.04E-12	1.31E-09
MNX1	0.775897	2.361488	1.605759	1.21E-07	1.31E-06
MIR4659A	0.049783	0.233132	2.227415	0.001955	0.004495
STRA8	0.010955	0.235457	4.425805	0.031135	0.049297
ARHGAP1	1.605964	6.419277	1.998971	5.75E-11	4.42E-09
AC027288	0.177671	0.710155	1.998924	0.00038	0.001093
FOXP2	2.15724	0.731829	-1.55961	0.000481	0.001337
AC007497	0.035796	0.212734	2.571183	1.22E-07	1.31E-06

RPL26P29	0.047992	0.247979	2.369355	8.87E-08	1.01E-06
SPINK7	43.74006	1.614241	-4.76003	0.00011	0.000377
AC027018	0.101741	0.305204	1.584867	3.20E-05	0.000133
CGB5	0.004942	1.193554	7.916087	9.92E-06	4.97E-05
TNFSF18	0.165601	0.519518	1.649464	0.002204	0.004987
NEK2	1.69626	7.699273	2.182365	9.87E-11	6.05E-09
STPG3	0.053931	0.218903	2.021097	4.10E-06	2.37E-05
AC008763	0.14926	0.44506	1.576174	0.000106	0.000366
PNMA5	0.016518	1.966951	6.89575	0.029928	0.047639
MIR7848	0.238865	0.733881	1.619354	0.012377	0.022127
AC012363	0.054187	0.750319	3.791491	1.00E-09	2.99E-08
TMEM211	1.104287	0.307139	-1.84615	0.00364	0.007707
IGF2BP2	3.736099	15.02838	2.008085	2.96E-09	6.79E-08
MAPK15	0.235365	2.020454	3.101705	1.31E-10	7.24E-09
AL138789.	0.033796	0.465757	3.784652	1.53E-08	2.44E-07
RNU4-53F	0.102475	0.343459	1.744862	0.002801	0.006136
AC078778	0.103966	0.300987	1.533596	2.33E-06	1.47E-05
MIR4296	0.062692	0.299947	2.258345	0.001233	0.003026
RPRM	2.913165	0.486733	-2.58139	5.34E-07	4.34E-06
MIR5192	0.044239	0.215022	2.281096	0.000581	0.001575
AC007342	0.361909	1.176783	1.701148	4.55E-05	0.000179
IGFBP1	0.043335	1.811591	5.385569	5.90E-06	3.22E-05
RNA5SP12	0.253553	0.730308	1.526217	0.000981	0.002484
AC021851	0.180288	0.51468	1.513375	1.30E-06	9.05E-06
PLN	70.17941	12.80396	-2.45446	5.58E-06	3.08E-05
SNORD14	0.639912	2.854085	2.157083	2.37E-05	0.000103
AP000753	0.099217	0.377543	1.927977	0.000112	0.000383
AC091153	0.055822	0.230642	2.046756	4.37E-06	2.51E-05
AP001052	0.522334	1.970049	1.915187	1.42E-07	1.48E-06
RPSAP52	0.023199	0.252823	3.445993	1.05E-12	1.31E-09
ST6GAL2	0.736186	0.259323	-1.50532	0.023298	0.038364
IGSF11	0.989894	0.205081	-2.27108	1.76E-05	8.00E-05
SOX21	13.35344	2.966468	-2.17039	0.000168	0.000541
AQP3	66.27134	15.35416	-2.10976	0.010258	0.018863
IGFBP6	62.05458	14.83475	-2.06456	3.56E-08	4.87E-07
CALHM3	0.031143	0.476372	3.935096	7.85E-05	0.000283
AC025575	0.126633	1.220675	3.26896	7.94E-06	4.12E-05
SGO2	0.738064	2.480964	1.749084	2.37E-10	1.05E-08
ADAMTS1	0.416774	2.837983	2.76753	3.68E-12	1.44E-09
NEGR1	4.256083	1.138391	-1.90253	1.07E-05	5.27E-05
TSPEAR-A	0.25783	1.124301	2.124534	1.30E-05	6.21E-05
FERMT1	7.122869	25.50295	1.840134	1.92E-08	2.95E-07
RHPN1	1.338257	4.581529	1.775474	2.24E-09	5.47E-08
ANGPTL1	14.10343	2.774065	-2.34597	1.59E-07	1.63E-06
RNU6-12(	0.117747	0.333691	1.502822	0.00034	0.000992
FOXJ1	0.656958	5.452907	3.053154	0.002312	0.005192
PHBP19	0.411873	1.256941	1.609644	1.27E-06	8.87E-06
LINC0173(	0.19976	0.568568	1.509068	0.000157	0.00051
AC073323	0.004929	0.219886	5.47922	6.24E-08	7.62E-07
BAMBI	1.292561	14.29984	3.467695	2.34E-07	2.21E-06
AP003086	0.081406	0.302016	1.891415	1.07E-06	7.75E-06
LINC0112:	0.270052	0.812503	1.589134	2.27E-08	3.38E-07
AP001574	0.068661	0.250678	1.868277	0.001572	0.003727
ARHGEF38	0.337348	1.582836	2.230204	1.69E-09	4.45E-08
RNY1P10	0.047524	0.308479	2.698459	8.44E-05	0.000301
NAP1L4P1	0.172549	0.551381	1.676045	2.78E-07	2.54E-06
DDIAS	0.539106	2.194159	2.025028	3.23E-10	1.30E-08
RBM24	1.888361	0.593866	-1.66892	0.00019	0.000602

COL10A1	0.801293	12.27075	3.93675	1.86E-12	1.40E-09
IL17C	0.251174	0.944127	1.910293	0.002582	0.005719
AC003965	0.374605	2.287455	2.610302	5.11E-07	4.19E-06
MAGEA10	0.006487	1.028896	7.309313	0.024855	0.040578
TNNC1	0.51391	3.135058	2.608905	9.74E-05	0.000339
MIR4479	0.189045	0.966421	2.353925	1.09E-06	7.86E-06
RCOR2	0.640539	3.045758	2.249443	1.84E-07	1.82E-06
FAM72D	0.112025	0.540524	2.270541	8.29E-10	2.63E-08
E2F7	0.38872	1.994159	2.358976	9.72E-11	6.00E-09
KIAA1549l	0.157122	0.611407	1.960245	0.001255	0.003072
C4BPA	0.091134	3.227459	5.146261	6.79E-08	8.14E-07
LYG2	0.130493	0.428028	1.713736	0.00011	0.000376
BMPER	1.290751	0.329931	-1.96797	9.09E-05	0.00032
HSPB7	34.90933	10.58993	-1.72092	0.000142	0.000471
LINC0191	0.020998	0.368129	4.131855	4.97E-06	2.79E-05
H19	6.093165	48.02937	2.978653	0.006674	0.013004
MMP7	3.11939	53.68137	4.105085	1.18E-06	8.36E-06
AC008395	0.100345	0.303333	1.595939	8.54E-05	0.000304
HOXC-AS	0.068303	0.987551	3.85384	5.90E-11	4.49E-09
AC135279	0.149138	0.48486	1.700921	2.73E-07	2.50E-06
TRARG1	1.024964	0.292782	-1.80768	3.70E-07	3.20E-06
RNU6-11	0.063054	0.326092	2.370622	0.004164	0.008656
SNORA71	0.465912	1.829601	1.9734	1.28E-09	3.59E-08
PNCK	5.972558	1.023307	-2.54511	1.39E-05	6.55E-05
AC087163	0.094339	0.298647	1.662517	2.96E-07	2.68E-06
SNORD37	0.096099	0.337992	1.814399	0.002563	0.005681
LINC0187	0.137799	0.520396	1.917047	0.00107	0.002672
AC104964	0.137668	0.438857	1.672557	0.000854	0.0022
GRIN2D	2.228471	10.87087	2.28634	2.45E-09	5.85E-08
PGM5	27.13256	5.56174	-2.28642	3.06E-07	2.75E-06
H2AFZP6	0.041934	0.219575	2.388523	8.29E-07	6.25E-06
TTC36	2.345762	0.559946	-2.0667	2.37E-05	0.000103
NUTF2P6	0.120596	0.345483	1.518426	4.15E-05	0.000166
AC139792	0.126078	0.364891	1.533149	0.003066	0.006634
AC009093	0.074153	0.211871	1.514619	1.44E-07	1.49E-06
AL391988.	0.555726	1.703881	1.616378	1.35E-07	1.42E-06
AC093583	0.601158	0.197716	-1.60431	0.025724	0.041812
RNU6ATA	0.161435	0.488055	1.59609	5.80E-05	0.000219
ULBP2	0.80537	2.388975	1.568669	3.78E-06	2.21E-05
PODNL1	0.681621	1.98055	1.53886	9.35E-07	6.92E-06
EPHX4	0.29961	1.205947	2.009008	8.91E-05	0.000315
PGA3	364.7984	28.01709	-3.70272	4.48E-05	0.000177
TMEM52B	0.094732	0.355438	1.907678	1.30E-08	2.18E-07
CTNNA3	1.953989	0.31788	-2.61987	0.006141	0.012113
HMMR	1.72481	6.692583	1.956126	4.25E-10	1.60E-08
