# Supplementary material for: An Immune-Related Gene Panel for Preoperative Lymph Node Status Evaluation in Advanced Gastric Cancer
Source: Biomed Res Int. 2020 Dec 7;2020:8450656. doi: 10.1155/2020/8450656 (PMC7789469; doi:10.1155/2020/8450656)
Supplement: Supplementary 2 — Table S2: the expression profile of differentially immune-related genes in gastric cancer. [file 8450656.f2.pdf]

| ID | conMean | treatMear | logFC | pValue | fdr |
|---|---|---|---|---|---|
| HSPA2 | 11.78369 | 3.067673 | -1.94158 | 0.00044 | 0.001238 |
| HSPA6 | 1.019355 | 3.486177 | 1.773989 | 0.001954 | 0.004493 |
| ULBP3 | 0.774111 | 2.2362 | 1.530437 | 6.15E-05 | 0.00023 |
| ULBP2 | 0.80537 | 2.388975 | 1.568669 | 3.78E-06 | 2.21E-05 |
| ULBP1 | 0.054206 | 0.391897 | 2.853961 | 5.13E-08 | 6.50E-07 |
| HAMP | 0.092722 | 0.373319 | 2.009418 | 1.61E-06 | 1.08E-05 |
| CXCL9 | 10.09992 | 47.04507 | 2.2197 | 2.66E-07 | 2.45E-06 |
| CXCL5 | 13.46146 | 71.81886 | 2.415528 | 0.006869 | 0.013331 |
| CXCL11 | 2.732417 | 10.67899 | 1.966526 | 4.29E-06 | 2.47E-05 |
| CXCL6 | 0.309888 | 4.176066 | 3.752326 | 1.11E-07 | 1.22E-06 |
| CXCL1 | 14.62557 | 61.3474 | 2.068509 | 6.72E-07 | 5.24E-06 |
| DEFA6 | 207.7274 | 11.42037 | -4.18501 | 0.017143 | 0.029393 |
| DEFA5 | 624.081 | 16.90695 | -5.20604 | 0.000805 | 0.00209 |
| LCN1 | 0.003408 | 0.569381 | 7.384423 | 4.37E-07 | 3.67E-06 |
| S100A8 | 795.2633 | 69.01211 | -3.52651 | 0.002424 | 0.005409 |
| PENK | 4.54293 | 0.643235 | -2.82021 | 0.006563 | 0.012827 |
| MMP12 | 10.51363 | 32.83861 | 1.643133 | 0.000215 | 0.000668 |
| TMSB15A | 0.173222 | 0.632444 | 1.868316 | 3.43E-07 | 3.01E-06 |
| S100B | 9.369454 | 2.209127 | -2.08449 | 2.64E-08 | 3.81E-07 |
| S100A7 | 3.811125 | 98.84484 | 4.696877 | 0.003483 | 0.007419 |
| LCN12 | 0.184019 | 1.040303 | 2.499079 | 4.63E-05 | 0.000182 |
| AZU1 | 0.033719 | 0.63675 | 4.239106 | 0.005281 | 0.010642 |
| PF4V1 | 0.095291 | 0.359416 | 1.915234 | 0.001862 | 0.004311 |
| FABP6 | 0.846414 | 2.635516 | 1.63865 | 0.000402 | 0.001144 |
| PLAU | 5.86371 | 27.41512 | 2.225086 | 1.29E-10 | 7.14E-09 |
| PAEP | 0.052277 | 1.333909 | 4.673347 | 6.13E-06 | 3.33E-05 |
| LBP | 0.076918 | 1.965733 | 4.675593 | 0.000773 | 0.002014 |
| NOX4 | 0.210662 | 0.623433 | 1.565304 | 4.73E-08 | 6.11E-07 |
| FABP4 | 15.87788 | 4.637632 | -1.77556 | 7.96E-09 | 1.48E-07 |
| R3HDML | 0.032939 | 0.333019 | 3.337752 | 2.58E-08 | 3.74E-07 |
| OBP2B | 0.011396 | 0.337705 | 4.889168 | 0.000156 | 0.00051 |
| PI15 | 0.474555 | 1.805573 | 1.927811 | 0.027859 | 0.044774 |
| NOX1 | 0.223876 | 4.702112 | 4.392538 | 1.38E-07 | 1.45E-06 |
| TFRC | 9.810024 | 30.19539 | 1.622 | 2.50E-08 | 3.63E-07 |
| GDF15 | 7.563278 | 41.78331 | 2.465843 | 1.24E-09 | 3.50E-08 |
| ADIPOQ | 1.615048 | 0.526981 | -1.61575 | 2.83E-08 | 4.02E-07 |
| CCL20 | 13.58526 | 49.59117 | 1.868041 | 0.003496 | 0.007441 |
| CHIT1 | 0.107316 | 1.361995 | 3.665783 | 0.000137 | 0.000455 |
| F2R | 6.261759 | 22.39161 | 1.838318 | 3.02E-11 | 3.13E-09 |
| CST4 | 0.019621 | 1.943703 | 6.630233 | 7.24E-12 | 2.00E-09 |
| CSRP1 | 118.0443 | 40.87557 | -1.53002 | 3.46E-05 | 0.000142 |
| TPM2 | 245.9354 | 73.90878 | -1.73446 | 0.002367 | 0.005301 |
| MSR1 | 1.050822 | 3.654397 | 1.798115 | 6.18E-08 | 7.56E-07 |
| SLC11A1 | 0.488257 | 1.584628 | 1.698431 | 7.55E-08 | 8.86E-07 |
| SEMG2 | 0.020346 | 0.291279 | 3.839565 | 0.00804 | 0.01528 |
| DES | 1352.684 | 426.9645 | -1.66364 | 5.97E-06 | 3.25E-05 |
| TNFSF11 | 0.162143 | 1.099128 | 2.761017 | 1.00E-10 | 6.06E-09 |
| RNASE3 | 0.041505 | 0.286695 | 2.788156 | 0.009209 | 0.017187 |
| PROC | 0.423036 | 2.01113 | 2.249153 | 0.000223 | 0.00069 |
| PCSK2 | 1.07589 | 0.298396 | -1.85023 | 1.22E-08 | 2.07E-07 |
| CCL18 | 10.06574 | 29.0937 | 1.531253 | 0.002134 | 0.004851 |
| CCR8 | 0.161647 | 0.597531 | 1.886166 | 1.61E-07 | 1.65E-06 |
| CCL21 | 126.7683 | 38.40042 | -1.723 | 1.15E-06 | 8.21E-06 |
| CCL7 | 0.154037 | 0.754276 | 2.291816 | 1.41E-09 | 3.88E-08 |
| FAM19A5 | 0.809813 | 2.514793 | 1.63478 | 3.09E-05 | 0.000129 |
| FAM19A4 | 6.570484 | 0.393004 | -4.06338 | 0.00013 | 0.000436 |
| CRP | 0.025347 | 1.169784 | 5.528257 | 0.005682 | 0.011331 |

| | | | | | |
|---|---|---|---|---|---|
| OLR1 | 0.531661 | 3.217443 | 2.597335 | 1.97E-10 | 9.38E-09 |
| RNASE2 | 0.5934 | 1.855076 | 1.644402 | 2.30E-05 | 0.0001 |
| IGHG4 | 45.35539 | 249.5981 | 2.460261 | 0.000219 | 0.000679 |
| IGHV3-16 | 1.889452 | 0.175984 | -3.42445 | 0.026853 | 0.043373 |
| IGKV1-37 | 1.19092 | 0.138424 | -3.10491 | 0.01293 | 0.023003 |
| IGKV1D-3 | 2.09835 | 0.164766 | -3.67077 | 0.003933 | 0.00824 |
| IGKV2-40 | 2.486435 | 0.449821 | -2.46666 | 0.019841 | 0.033378 |
| IGKV2D-3 | 29.15886 | 2.540701 | -3.52064 | 0.024282 | 0.039806 |
| IGKV3D-7 | 13.07182 | 1.149693 | -3.50714 | 0.009502 | 0.017657 |
| IGLJ1 | 3.150749 | 0.808226 | -1.96286 | 0.015851 | 0.027483 |
| IGLV1-50 | 13.77548 | 1.804362 | -2.93254 | 0.026284 | 0.042553 |
| IGLV3-32 | 1.260702 | 0.175022 | -2.84862 | 0.006288 | 0.012357 |
| CMA1 | 1.758077 | 0.398143 | -2.14264 | 1.40E-08 | 2.29E-07 |
| CXCL17 | 225.645 | 66.30906 | -1.76678 | 0.000638 | 0.001706 |
| EDN2 | 2.576088 | 0.701798 | -1.87605 | 0.000391 | 0.001118 |
| EDN3 | 8.277222 | 2.255778 | -1.87552 | 0.00085 | 0.002191 |
| SAA2 | 1.107449 | 4.378332 | 1.983141 | 0.011721 | 0.021132 |
| SEMA5B | 0.165185 | 0.562405 | 1.767529 | 8.43E-09 | 1.55E-07 |
| SEMA6D | 3.088962 | 1.056825 | -1.54739 | 0.002859 | 0.006241 |
| AGT | 5.159137 | 26.56899 | 2.364542 | 3.45E-06 | 2.04E-05 |
| AMH | 0.259317 | 1.867736 | 2.848503 | 2.46E-06 | 1.54E-05 |
| ANGPTL7 | 2.138054 | 0.414511 | -2.36682 | 6.54E-05 | 0.000242 |
| APLN | 0.466807 | 3.148749 | 2.75388 | 2.41E-09 | 5.81E-08 |
| BMP3 | 3.70352 | 1.013422 | -1.86966 | 0.007751 | 0.014806 |
| BMP8A | 0.60697 | 2.600769 | 2.09924 | 1.74E-10 | 8.46E-09 |
| CGB5 | 0.004942 | 1.193554 | 7.916087 | 9.92E-06 | 4.97E-05 |
| CSF2 | 0.058713 | 0.997896 | 4.087149 | 1.50E-08 | 2.42E-07 |
| ESM1 | 0.158903 | 2.937778 | 4.208508 | 1.36E-13 | 1.20E-09 |
| FAM3B | 21.75096 | 7.650915 | -1.50737 | 0.001 | 0.002519 |
| FGF19 | 0.009225 | 2.492638 | 8.077888 | 1.25E-07 | 1.34E-06 |
| FGF3 | 0.0215 | 2.255139 | 6.712726 | 0.000347 | 0.001009 |
| GDF7 | 0.544006 | 0.179531 | -1.59939 | 1.96E-05 | 8.75E-05 |
| GHRL | 16.46639 | 2.613006 | -2.65574 | 0.017166 | 0.029411 |
| GIP | 25.6534 | 0.605382 | -5.40516 | 0.000163 | 0.000526 |
| GKN1 | 5854.337 | 282.2414 | -4.3745 | 8.18E-05 | 0.000293 |
| IL11 | 0.266487 | 2.800497 | 3.393548 | 5.14E-11 | 4.19E-09 |
| IL17C | 0.251174 | 0.944127 | 1.910293 | 0.002582 | 0.005719 |
| INHBA | 1.020371 | 9.136558 | 3.162557 | 3.11E-12 | 1.40E-09 |
| INHBB | 1.612886 | 6.107123 | 1.920849 | 0.007379 | 0.014189 |
| LEFTY1 | 0.240377 | 6.431912 | 4.741878 | 0.00359 | 0.007613 |
| LIF | 1.508628 | 7.601252 | 2.333 | 2.23E-10 | 1.02E-08 |
| NPPC | 2.565739 | 0.404066 | -2.66671 | 2.28E-07 | 2.16E-06 |
| NPY | 2.723065 | 0.921326 | -1.56345 | 3.07E-05 | 0.000128 |
| NRG2 | 0.877363 | 0.272125 | -1.6889 | 5.51E-06 | 3.05E-05 |
| OSM | 0.580844 | 2.134351 | 1.877574 | 0.000137 | 0.000455 |
| PNOC | 1.999265 | 0.603903 | -1.72708 | 0.000154 | 0.000503 |
| PTN | 18.63078 | 5.014096 | -1.89363 | 2.11E-07 | 2.05E-06 |
| SPP1 | 11.78024 | 85.34403 | 2.856921 | 3.33E-08 | 4.61E-07 |
| SST | 112.1308 | 35.07082 | -1.67684 | 5.15E-05 | 0.000199 |
| STC2 | 0.477216 | 1.685847 | 1.820758 | 6.79E-08 | 8.14E-07 |
| TAC1 | 2.42615 | 0.673154 | -1.84966 | 0.00221 | 0.005001 |
| TNFRSF11 | 0.506766 | 5.18658 | 3.355391 | 1.06E-07 | 1.18E-06 |
| TNFSF15 | 1.0076 | 3.098271 | 1.62054 | 1.61E-07 | 1.65E-06 |
| TNFSF18 | 0.165601 | 0.519518 | 1.649464 | 0.002204 | 0.004987 |
| TSLP | 0.751837 | 0.191789 | -1.9709 | 5.06E-07 | 4.14E-06 |
| UCN2 | 0.079133 | 0.450011 | 2.507612 | 3.75E-08 | 5.07E-07 |
| UTS2 | 0.072096 | 0.549633 | 2.930478 | 0.000286 | 0.000856 |
| VIP | 16.97405 | 2.916114 | -2.54121 | 4.44E-05 | 0.000175 |

| | | | | | |
|---|---|---|---|---|---|
| ADCYAP1I | 1.084637 | 0.149072 | -2.86313 | 4.63E-07 | 3.85E-06 |
| ADRB2 | 2.161302 | 0.620559 | -1.80026 | 1.71E-10 | 8.37E-09 |
| AGTR1 | 2.460755 | 0.752889 | -1.70859 | 1.58E-05 | 7.31E-05 |
| ANGPTL1 | 14.10343 | 2.774065 | -2.34597 | 1.59E-07 | 1.63E-06 |
| ESRRG | 2.437658 | 0.345677 | -2.818 | 3.33E-08 | 4.61E-07 |
| FGFR4 | 4.578763 | 17.78795 | 1.957871 | 6.52E-08 | 7.88E-07 |
| GHR | 2.547057 | 0.746877 | -1.76989 | 7.35E-08 | 8.68E-07 |
| IL13RA2 | 0.098766 | 0.874795 | 3.146855 | 1.65E-06 | 1.10E-05 |
| IL17RB | 2.770569 | 8.772599 | 1.662822 | 1.12E-08 | 1.92E-07 |
| LGR5 | 0.794661 | 6.517745 | 3.035961 | 5.12E-05 | 0.000198 |
| MC1R | 0.214139 | 0.638226 | 1.57552 | 3.23E-09 | 7.25E-08 |
| MET | 4.709488 | 24.7771 | 2.395365 | 5.94E-11 | 4.49E-09 |
| MLNR | 2.258565 | 0.388486 | -2.53947 | 0.014604 | 0.025587 |
| NR4A3 | 11.70857 | 2.75722 | -2.08628 | 0.025886 | 0.041997 |
| NR6A1 | 0.360443 | 1.111653 | 1.624864 | 1.61E-07 | 1.65E-06 |
| OXTR | 0.179712 | 0.763262 | 2.08649 | 2.96E-09 | 6.79E-08 |
| PGR | 0.841656 | 0.259352 | -1.69832 | 0.000622 | 0.001667 |
| SSTR5 | 0.133319 | 0.880997 | 2.724251 | 0.007011 | 0.013576 |
| TUBB3 | 0.110974 | 0.54119 | 2.285914 | 6.61E-08 | 7.96E-07 |
| FCGR3A | 4.877209 | 17.61283 | 1.852499 | 1.34E-08 | 2.22E-07 |
| PRKCG | 0.409128 | 1.818149 | 2.151847 | 4.65E-06 | 2.63E-05 |
| TRAJ2 | 0.193557 | 0.762493 | 1.977968 | 0.000927 | 0.002362 |
| TRAJ5 | 0.094706 | 0.383594 | 2.018055 | 0.003422 | 0.007301 |
| TRAJ23 | 0.108654 | 0.351885 | 1.69536 | 0.005607 | 0.011193 |
| TRAJ36 | 0.062504 | 0.483457 | 2.951363 | 0.001055 | 0.002641 |
| TRAJ37 | 0.082524 | 0.427492 | 2.373016 | 0.001591 | 0.003764 |
